# Calcium-Impregnated Silica Gel as a Reducing Agent in Domino Reactions for Bond Formations

**DOI:** 10.1021/acs.joc.6c00118

**Published:** 2026-04-20

**Authors:** Khagendra Prasad Bohara, Animesh Roy, Jih Ru Hwu

**Affiliations:** Department of Chemistry and Frontier Research Center on Fundamental and Applied Sciences of Matters, 34881 National Tsing Hua University, Hsinchu 300, Taiwan

## Abstract

The reagent Ca@SiO_2_ was obtained by impregnation of silica gel with calcium metal. The presence of Ca@SiO_2_ allowed aldehydes to condense with ketones in 2-MeTHF, which resulted in α,β-unsaturated cyclohexenones in 73–90% yields through a radical domino process. Furthermore, Ca@SiO_2_ was used to induce a Darzens reaction for the efficient production of α-keto *trans*-epoxides from benzaldehydes and α-bromo ketones. Being a free-flowing powder, Ca@SiO_2_ was easy to handle and facilitated chemoselectivity among many functional groups.

## Graphical abstract

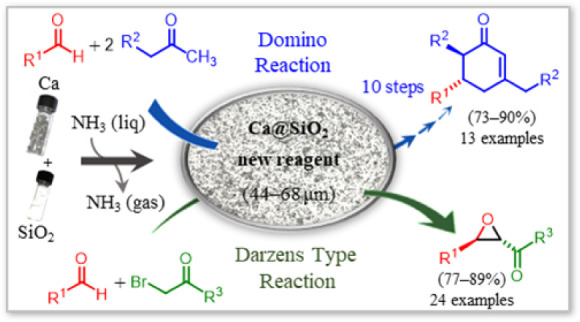

Metal reduction is among the most popular reactions in chemistry. The alkali metals, including lithium, sodium, and potassium, as well as the alkaline earth metal calcium, are used in reductions by dissolving the metals in liquid ammonia. Calcium metal can reduce aldehydes, ketones, enones, esters, indoles, arenes, buckminsterfullerenes, pyridine *N*-oxides, allyl ethers, benzyl ethers, benzyl alcohols, alkynes, nitriles, etc.^1–3^ Furthermore, it can cleave various chemical single bonds, such as the C–O in dihydropyrans, (O=C)­C–OAc, and R_2_N­(O=C)­C–O­(C=O)­R; the N–O in organic nitric oxides R^1^_2_N–OR^2^; the C–S in (R_2_NCO)­C–S; and the C–N in R^1^_2_PhC–NR^2^_2_. Calcium metal is also applied in *O*-debenzylation, conversion of epoxides to alcohols, desulfonylation, removal of thiophenyl and sulfonyl groups, dithiolane removal from an allylic position, hydrogenation, irreversible tandem aldol addition, dehydration, Michael addition, and so forth.^1–4^

The reducing strength of dissolved metals follows the order Li > K > Rb > Na > Ca. Thus, Ca exhibits a superior chemoselective reduction among different functional groups.^1^ For example, van Tamelen et al.^5^ developed a method for the selective removal of a benzyl group from an ether moiety in a polyene containing a nonterminal C–C triple bond during the total synthesis of sterols. In the total synthesis of (−)-solavetivone,^6,7^ a key step involves the selective removal of a 1,3-dithiolane moiety from an allylic position in an octalin containing a (Me_3_Si)­Me_2_Si– group. Calcium in liquid ammonia with ether as the cosolvent serves the purpose well, which cannot be accomplished by the use of Na in liquid ammonia.

Calcium metal reacts vigorously with water to liberate hydrogen gas and form Ca­(OH)_2_ at room temperature. Fine Ca metal spontaneously burns in air to produce the corresponding nitrides.^8^ Therefore, we planned to develop a new, mild, and easy-to-use Ca reagent for chemical reactions.

Alkali metals (including Na and K), as well as their alloys (including Na_2_K and K_2_Na), loaded on inert supports,^9–13^ have been applied for the reduction of various compounds. Silica gel (SiO_2_) is impregnated with various reagents such as activated carbon,^14^ Brønsted acids,^15^ ceric ammonium nitrate,^16,17^ potassium hydroxide,^18^ silver nitrate,^19^ tantalum­(V) oxide,^20^ transition metal salts,^21^ and others. Often, “reagents@SiO_2_” exhibit advantages over myriad other methods for the generation of the desired products with better yields under milder conditions.^10,16,17^

Commercially available Ca metal is in the form of turnings, granules, pieces (size < 1 cm), and dendritic pieces. Accurate measurement of these reagents is difficult for their applications in chemoselective reductions. To address this problem, we invented the Ca@SiO_2_ reagent as fine powders by impregnating Ca metal onto SiO_2_. Its new applications are the reductive annulation displayed in Scheme 1(A) and the epoxide formation with chemoselectivity shown in Scheme 1(B).

**Scheme 1:**
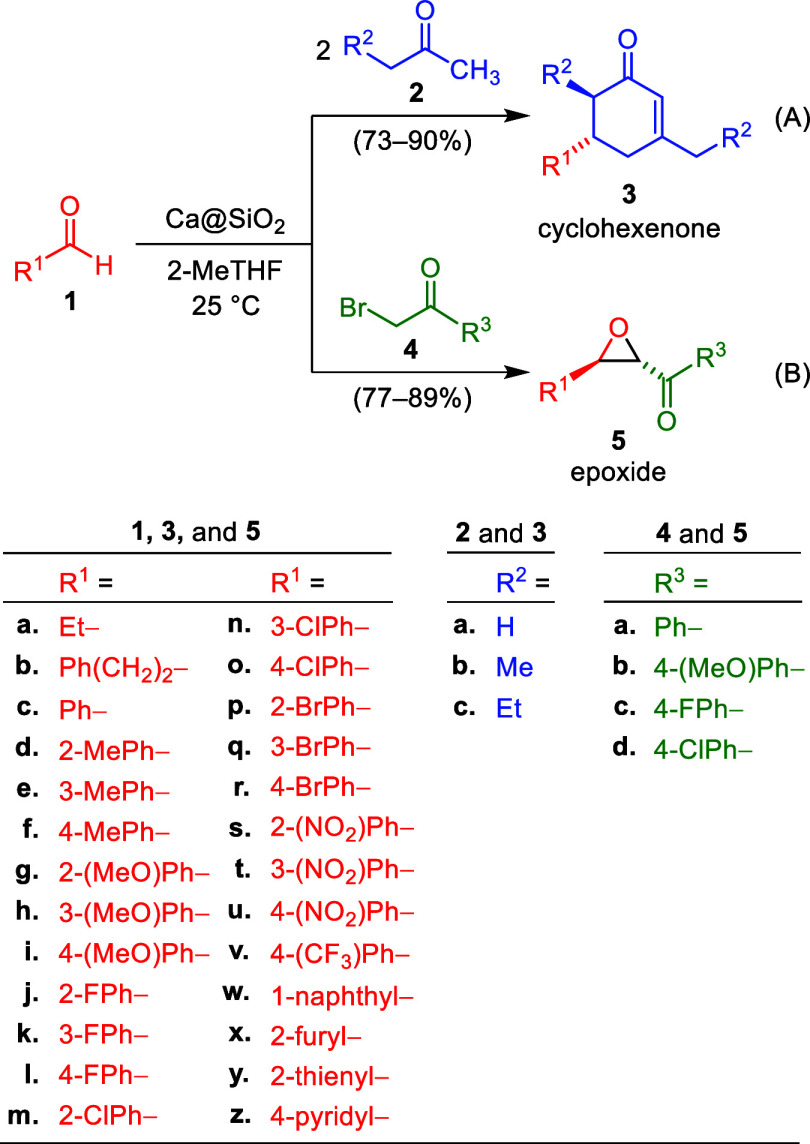
Ca@SiO_2_ Initiated Syntheses of α,β-Unsaturated Cyclohexenones 3 and α-Keto *trans*-Epoxides 5 from Aldehydes 1 and Ketones 2 or 4, Respectively

We placed the requisite amount of Ca granules (Figure 1(a)) and dried silica gel (40–63 μm, 230–400 mesh, Figure 1(b)) into a flask fitted with a Dewar condenser. The flask was cooled to −78 °C and purged with ammonia gas, where the gas was condensed into liquid to dissolve the Ca granules. After a blue slurry was formed, it was stirred for 30 min, and the liquid ammonia was then evaporated to give gray free-flowing powders, as displayed in Figure 1(c). The gray powders turned into white powders, as shown in Figure 1(d) after being exposed to air for 24 h. Confocal microscopy revealed the size of the Ca@SiO_2_ product^2b^ to be 44–68 μm, as shown in Figure 1(e).

**Figure 1:**
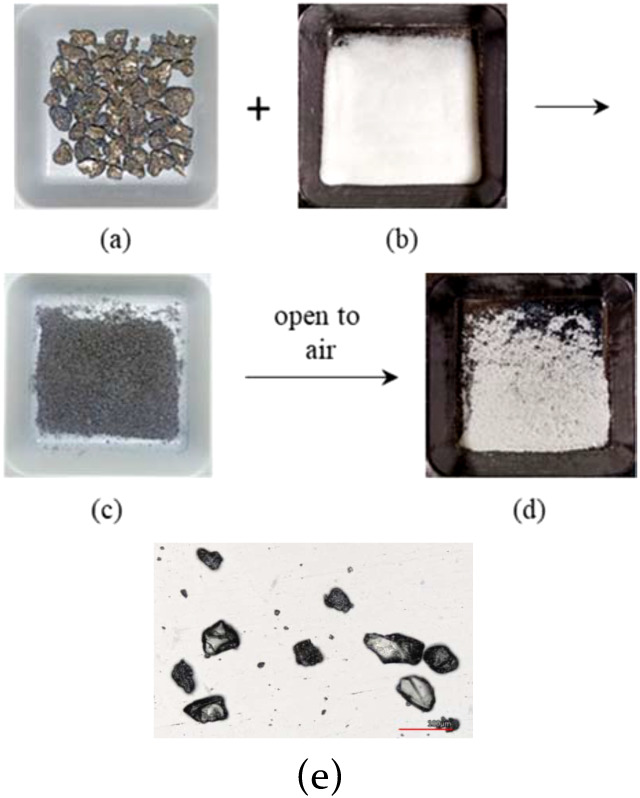
(a) Commercially available Ca granules, (b) dry silica gel (40–63 μm), (c) freshly prepared Ca@SiO_2_ powders, (d) white powders after Ca@SiO_2_ is exposed to air for 24 h, and (e) confocal microscopy image of Ca@SiO_2_ powders.

The Ca@SiO_2_ reagent exhibited identical reducing activity for up to 6 months when stored at room temperature in an airtight glass bottle. The same procedure was successfully followed for the preparation of the Ca@SiO_2_ powders containing various weight percentages (i.e., 20%, 30%, 40%, 50%, and 60%) of Ca in silica gel. The procedure should be carefully performed under anhydrous conditions inside a fume hood under an argon atmosphere to avoid fire hazards.

For performing reductive annulation, we initially generated an annulation product **3ca** through the reaction of benzaldehyde (**1c**, 1.0 equiv) with an excess of acetone (**2a**, 8.0–14 equiv) in the presence of Ca@SiO_2_ (40.0% of Ca by weight, 5.0–8.0 equiv) at 25 °C (Scheme 1(A)). THF, 2-MeTHF, and acetonitrile were employed individually as solvents. After the reaction mixture was stirred under dry nitrogen gas for 48 h, the inorganic residue was filtered off to produce α,β-unsaturated cyclohexenone **3ca** in 60–81% yields. The best result was obtained when the reaction was performed in the presence of 6.0 equiv of Ca@SiO_2_ in 2-MeTHF under anhydrous conditions.

We found that particle sizes in the range of 44–68 μm provided an optimal balance among surface area, reagent dispersion, and mass transfer. These Ca@SiO_2_ reagents enabled efficient interaction with substrates for electron transfer. In contrast, both larger and smaller particle sizes led to aggregation and poor handling, thereby adversely affecting reproducibility.

In a control experiment, we added 2,2,6,6-tetramethyl-1-piperidinyl*-N-*oxide^22^ (TEMPO, 6.0 equiv) as a radical scavenger in the annulation reaction. The desired cyclohexenone **3ca** was not detected. In the presence of TEMPO with 2.0 equiv, we isolated the acetone–TEMPO coupling product **8** (shown in Scheme 2) and **3ca** in 14% and 42% yields, respectively. These results indicated a radical pathway for the reductive annulation reaction by using Ca@SiO_2_ as the reagent.

**Scheme 2:**
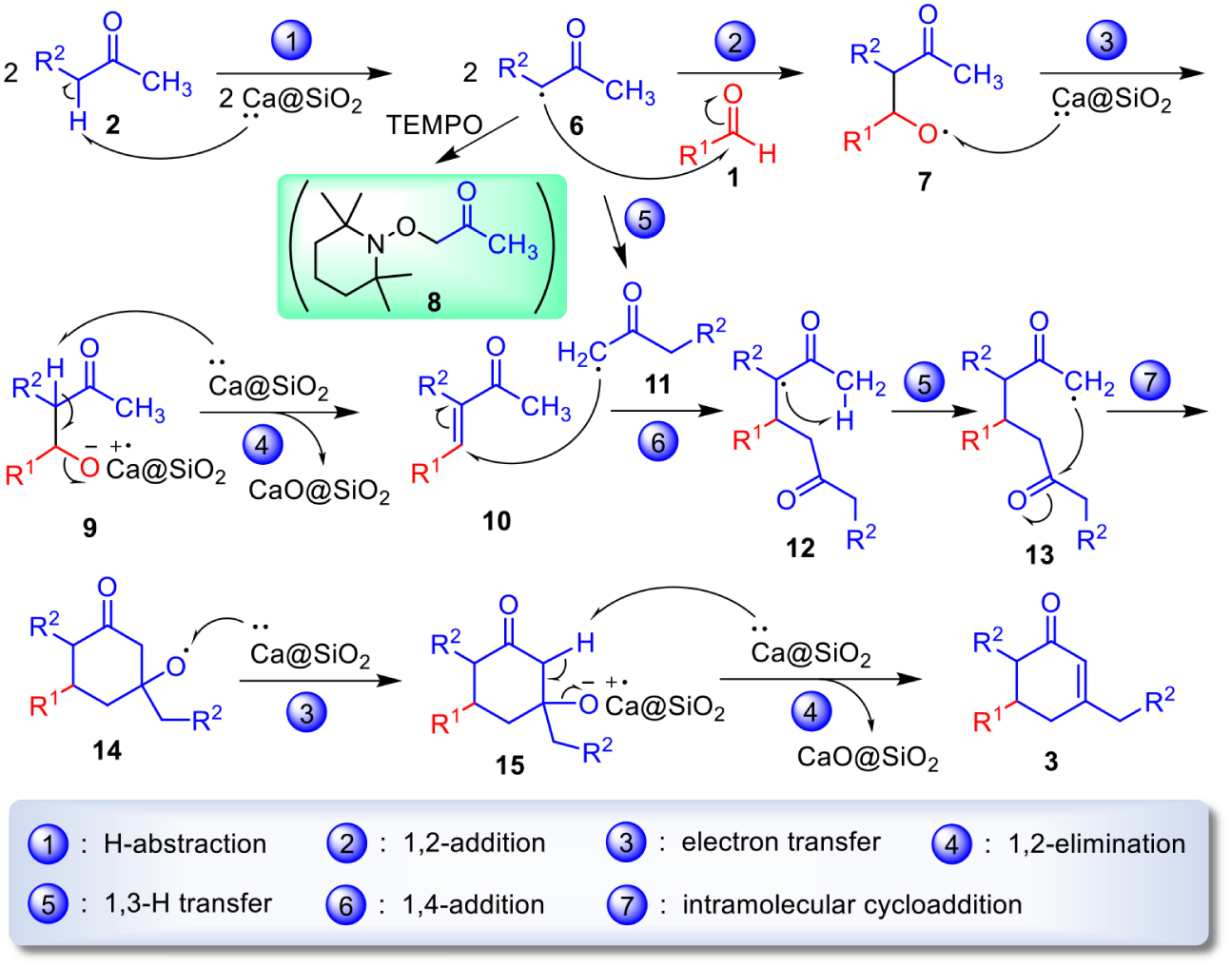
A Plausible Mechanism for the Formation of α,β-Unsaturated Cyclohexenones 3 from Aldehydes 1 and Ketones 2 in the Presence of Ca@SiO_2_

Optimized reaction conditions applied to aldehydes **1** produced 13 corresponding α,β-unsaturated cyclohexenones (see Figure 2(a)) in 73–90% yields. Aldehydes **1** included *n*-propionaldehyde (**1a**); 3-phenylpropionaldehyde (**1b**); benzaldehydes bearing substituents such as Me, OMe, F, Cl, and NO_2_ groups (in **1f**, **1i**, **1l**, **1o**, and **1u**, respectively); 2-furancarboxaldehyde (**1x**); 2-thiophenecarboxaldehyde (**1y**); and 4-pyridinecarboxaldehyde (**1z**).

**Figure 2:**
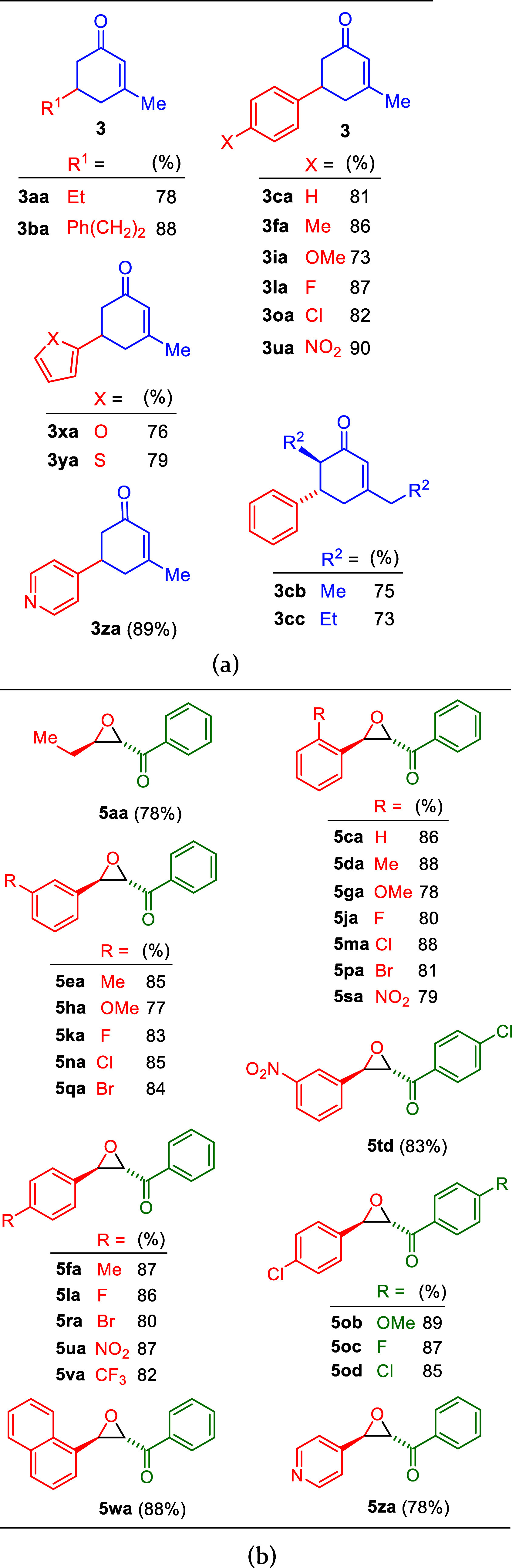
Chemical structures and the obtained yields of (a) α,β-unsaturated cyclohexenones and (b) α-keto epoxides.

To evaluate the reducing capability and chemoselectivity of the Ca@SiO_2_ reagent further, we performed the Darzens-type reaction^23^ displayed in Scheme 1(B). The first step was to determine the best weight% of Ca in Ca@SiO_2_. Thus, a mixture of benzaldehyde (**1c**, 1.0 equiv) and α-bromo ketone **4a** (1.1 equiv) was treated with Ca@SiO_2_ (2.0 equiv) in THF, 2-MeTHF, or acetonitrile at 25 °C for 4.0 h. The applied reducing reagents contained 20%, 30%, 40%, 50%, and 60% Ca metal by weight in Ca@SiO_2_. The use of 40.0% calcium in Ca@SiO_2_ resulted in the best yield (86%) for α-keto *trans*-epoxide **5ca**. This ratio was followed for its applications in all other related reactions.

In the second step, we determined the required equivalents of Ca@SiO_2_ for the generation of α-keto epoxide **5ca** with the highest yield in the Darzens-type reaction. Being an alkaline earth metal, Ca is capable of donating two electrons during a reduction reaction. Therefore, the use of an excess of Ca@SiO_2_ might reduce the two oxygen-containing functional groups therein. Our experiment involving the use of 2.0 equiv of Ca@SiO_2_ in 2-MeTHF led to the desired product **5ca** in 86% yield.

On the basis of the optimum conditions, 24 α-keto epoxides, shown in Figure 2(b), were generated in 77–89% yields from various aliphatic and aromatic aldehydes **1** (1.0 equiv), α-bromo ketones **4** (1.1 equiv), and Ca@SiO_2_ (40.0 weight% of Ca, 2.0 equiv). The phenyl ring in the starting materials may have various electron-withdrawing and -donating substituents, including Me, CF_3_, OMe, F, Cl, Br, and NO_2_ at the *ortho-*, *meta*-, or *para*-positions. In addition to benzaldehydes, the use of 1-naphthaldehyde and 4-pyridinecarboxaldehyde as the starting materials also provided good yields of the corresponding α-keto epoxides **5wa** (88%) and **5za** (78%), respectively.

Regarding the characteristics of the Ca@SiO_2_ reagent, these gray, free-flowing powders did not cling to one another to form aggregates. The noncohesive property made this reagent easy to handle and permitted accurate measurement under standard laboratory conditions. Its activity and appearance remained nearly unchanged after storage in a sealed glass bottle flushed with nitrogen gas and kept in a desiccator for ∼12 months.

We were able to obtain cyclohexenone **3ca** in 81% yield by adding 6.0 equiv of Ca@SiO_2_ to the reaction mixture and, meanwhile, noted Ca underwent two oxidative processes. These are Ca → Ca^+^ + e^–^ and Ca^+^ → Ca^2+^ + e^–^. The additional 6.0 equiv of electrons, potentially generated by the process of Ca^+^ → Ca^2+^ + e^–^, were not observed to reduce any species further in the reaction media.

Similarly, 2.0 equiv of Ca@SiO_2_ was used for the formation of the Darzens-type product **5ca**. Therefore, 2.0 equiv of electrons in excess existed in the reaction mixture. Under these conditions, we were still able to isolate keto epoxide **5ca** in good yield (86%), despite the presence of two reducible ketonic and epoxy functional groups in the final products. These results confirm the mild character and, thus, the high chemoselectivity of the Ca@SiO_2_ reagent. The F, Cl, Br, CF_3_, and NO_2_ groups in benzaldehydes **1** remained intact when Ca@SiO_2_ was applied. Consequently, these starting materials were successfully converted to enones **3** and keto epoxides **5**, as shown in Scheme 1(A) and Scheme 1(B).

The preparation of the Ca@SiO_2_ reagent was carried out in liquid ammonia at −78 °C. This procedure required a substantial quantity of liquid ammonia. Nevertheless, ammonia was used solely during reagent formation to dissolve calcium metal. Once Ca@SiO_2_ was generated, the ammonia was removed to afford gray, free-flowing powders. Notably, the two synthetic transformations illustrated in Scheme 1 were performed under ammonia-free conditions. Furthermore, calcium is an abundant, inexpensive, and relatively low-toxicity metal. Collectively, these features enhance the practical utility of the Ca@SiO_2_ reagent and highlight its potential for future green applications.

The mechanism presented in Scheme 2 accounts for our design regarding the use of Ca@SiO_2_ in the synthesis of α,β-unsaturated cyclohexenones **3** from aldehydes **1** and ketones **2**. In the initial step, the first two equivalents of Ca@SiO_2_ remove hydrogen atoms from two equivalents of ketones **2** to generate two equivalents of α-ketonic radicals **6**.^24^ After the 1,2-addition of one equivalent of radicals **6** to aldehydes **1**, the resultant aldol radicals **7** accept one electron^25^ from the third equivalent of Ca@SiO_2_ to form the salt species **9**. The fourth equivalent of Ca@SiO_2_ initiates a 1,2-elimination^26^ by donating an electron to species **9**, which leads to enone intermediates **10** and calcium oxide impregnated silica gel (i.e., CaO@SiO_2_).

The remaining second equivalent of α-ketonic radicals **6** was able to undergo an intramolecular 1,3-hydrogen-atom transfer^27^ to provide the terminal α-ketonic radicals **11**. The 1,4-addition of radicals **11** onto enones **10** resulted in another set of α-ketonic radicals **12**, in which a hydrogen atom of the acetyl group underwent an intramolecular 1,3-hydrogen-atom transfer again to afford the terminal carboradicals **13**. These intermediates held an ideal geometry for intramolecular radical cyclization to generate the tertiary alkoxy radicals **14**. By following a similar transfer procedure of **7** → **9** and the 1,2-elimination step of **9** → **10**, the presence of the fifth and sixth equivalents of Ca@SiO_2_ enabled the formation of the cyclohexenones **3** from alkoxy radicals **14** via the intermediates **15**.

The whole process, from the starting materials aldehydes **1** and ketones **2** to the final targets **3,** included 10 mechanistic steps. Therefore, this radical reaction was a domino process, which involved hydrogen atom abstraction, 1,2-addition, two-electron-transfer steps, two 1,2-elimination steps, two intramolecular 1,3-hydrogen-atom transfers, a 1,4-addition, and intramolecular radical cyclization.

The reaction mechanism shown in Scheme 3 can account for the Darzens reaction initiated by the Ca@SiO_2_ reagent for the condensation of aldehydes **1** with α-bromo ketones **4** to afford α-keto epoxides **5**.^10^ The first equivalent of Ca@SiO_2_ removes a bromo group from bromo ketones **4** to provide acetyl radicals **16**. Addition of radicals **16** to aldehydes **1** produces alkoxy radicals **17**. Finally, the second equivalent of Ca@SiO_2_ initiates an epoxide formation through a hydrogen atom abstraction to generate α-keto epoxides **5** with the trans configuration. The stereoselectivity has been illustrated previously.^10^

**Scheme 3:**
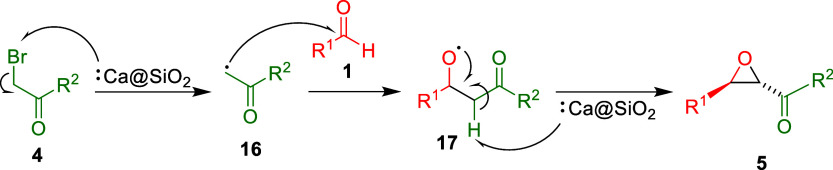
Ca@SiO_2_ Used in the Darzens-type Reaction for the Synthesis of α-Keto *trans*-Epoxides

In conclusion, calcium metal-impregnated silica gel is invented as a mild reducing reagent with high chemoselectivity. This Ca@SiO_2_ reagent, as free-flowing powders, efficiently promotes the reductive annulation of aldehydes **1** and ketones **2** in 2-MeTHF at 25 °C to produce α,β-unsaturated cyclohexenones **3** in 73–90% yields. It can also convert a mixture of benzaldehydes **1** and α-bromo ketones **4** to α-keto epoxides **5** in 77–89% yields.

The advantages of this Ca@SiO_2_ reagent applied in synthetic works include: (1) Its noncohesive property facilitates convenient handling and ensures accurate measurement in its powder state. (2) Ca@SiO_2_ is sufficiently active for the formation of C–C and C–O bonds at room temperature in a short period of time. (3) Manipulation of the heterogeneous reactions containing the Ca@SiO_2_ reagent is operationally simple. (4) The mild reducing capability of Ca@SiO_2_ enables high chemoselectivity among various functional groups. (5) The eco-friendly solvent 2-MeTHF can be used as the solvent along with the Ca@SiO_2_ reagent. Their utilization in combination aligns with contemporary green chemistry principles.

## Supplementary Material



## Data Availability

The data underlying this study are available in the published article and its Supporting Information.

## Notes

Experimental details, spectroscopic data of all new compounds, references, and spectra (PDF)
