# Supplementary material for: Calcium-Impregnated Silica Gel as a Reducing Agent in Domino Reactions for Bond Formations
Source: J Org Chem. 2026 Apr 20;91(17):6151–6. doi: 10.1021/acs.joc.6c00118 (PMC13140140; doi:10.1021/acs.joc.6c00118)
Supplement: Supplementary file 1 [file jo6c00118_si_001.pdf]

Supporting Information

Calcium-Impregnated Silica Gel as a Reducing Agent in Domino Reactions for Bond Formations

Khagendra Prasad Bohara, Animesh Roy, and Jih Ru Hwu*

Department of Chemistry and Frontier Research Center on Fundamental and Applied Sciences of Matters, National Tsing Hua University, Hsinchu 300, Taiwan

Email: jrhwu@mx.nthu.edu.tw

Contents

General Information **S2**

Preparation of Calcium Metal Impregnated Silica gel (Ca@SiO_2) **S3**

The Standard Procedure 1 for the Synthesis of Cyclohexenones 3 .... **S4**

The Standard Procedure 2 for the Synthesis of *α*-Keto Epoxides 5 .... **S12**

Table S1. Optimization of Reaction Conditions for 1c + 2a → 3ca in the Presence of Ca@SiO_2 .... **S29**

Table S2. Optimization of Reaction Conditions for 1c + 4a → 5ca in the Presence of Different wt % of Ca in Ca@SiO_2 .... **S30**

Table S3. Optimization of Reaction Conditions for 1c + 4a → 5ca with Different Equivalents of Ca Metal in Ca@SiO_2 .... **S31**

References **S31**

Spectra of Compounds **S34**

General Information

All reactions were carried out in oven-dried glassware (120 °C) under an atmosphere of nitrogen unless as indicated otherwise. Acetonitrile, ethyl acetate, and hexanes from Mallinckrodt Chemical Co. were dried and distilled from CaH_2. Diethyl ether (Et_2O) and tetrahydrofuran (THF) from Mallinckrodt Chemical Co. were dried by distillation from sodium and benzophenone under an atmosphere of nitrogen. 2-Methyltetrahydrofuran (2-MeTHF) from Sigma-Aldrich was dried by distillation from sodium and benzophenone under an atmosphere of nitrogen. The reagents purchased from Alfa Aesar included benzaldehyde, 2-chlorobenzaldehyde, 4-chlorobenzaldehyde, 4-fluorobenzaldehyde, 4-methoxybenzaldehyde, 2-nitrobenzaldehyde, 4-nitrobenzaldehyde, and (2,2,6,6-tetramethylpiperidin-1-yl)oxyl (TEMPO). The reagents purchased from Sigma-Aldrich included acetone, 2-bromobenzaldehyde, 3-bromobenzaldehyde, 2-bromo-4′-chloroacetophenone, 2-bromo-4′-fluoroacetophenone, 2-bromo-4′-methoxyacetophenone, 2-butanone, 3-chlorobenzaldehyde, 2-fluorobenzaldehyde, 3-fluorobenzaldehyde, 2-furancarboxaldehyde, 2-methoxybenzaldehyde, 3-methoxybenzaldehyde, 2-methylbenzaldehyde, 3-methylbenzaldehyde, 4-methylbenzaldehyde, 1-naphthaldehyde, 3-nitrobenzaldehyde, 2-pentanone, 3-phenylpropionaldehyde, 4-pyridinecarboxaldehyde, 2-thiophenecarboxaldehyde, and 4-(trifluoromethyl)benzaldehyde. The reagents purchased from Tokyo –emical Industry Co. included 2-bromoacetophenone and 4-bromobenzaldehyde. Calcium metal was purchased from Ferak Berlin.

Analytical thin-layer chromatography (TLC) was performed on precoated plates (silica gel 60 F-254). Purification by gravity column chromatography was carried out by use of Silicycle ultrapure silica gel (particle size 40–63 μm, 230–400 mesh).

Infrared (IR) spectra were recorded on a Fourier transform infrared (FT-IR) spectrometer. Absorption intensities are recorded by the following abbreviations: s, strong;

m, medium; and w, weak. Proton NMR spectra were obtained on 400 MHz spectrometer by use of chloroform-*d* ($CDCl_3$) as the solvent. Proton NMR chemical shifts were referenced to the residual protonated solvent (δ 7.24 ppm for chloroform). Carbon-13 NMR spectra were obtained on 100 MHz spectrometer by use of chloroform-*d* ($CDCl_3$) as the solvent. Carbon-13 chemical shifts were referenced to the center of the $CDCl_3$ triplet (δ 77.0 ppm). Multiplicities are recorded by the following abbreviations: s, singlet; d, doublet; dd, doublet of doublet; t, triplet; q, quartet; m, multiplet; and *J*, coupling constant (hertz). High-resolution mass spectra (HRMS) were measured on an instrument by use of a time-of-flight (TOF) mass analyzer with electrospray ionization (ESI).

Preparation of Calcium Metal Impregnated Silica gel (Ca@SiO_2). Calcium granules (8.21 g) and the oven-dried (110 °C for 4.0 h) silica gel (12.3 g, Geduran Si 60, particle size 40–63 μm, 230–400 mesh) were taken in a three-necked flask equipped with a magnetic stirring bar and rubber septa under argon atmosphere. To this flask was fitted a Dewar condenser equipped with a drying tube loaded with KOH pellets. The condenser and the flask were cooled to −78 °C by use of liquid nitrogen–acetone bath. Ammonia (g) was passed and condensed into the flask to dissolve calcium granules. After ammonia (~95 mL) was condensed, a blue slurry formed was stirred at −78 °C for 30 min. The slurry was allowed to warm to room temperature with the simultaneous evaporation of ammonia under argon atmosphere. The traces of ammonia were then removed under reduced pressure (overnight) to afford grey colored free-flowing powders, which contained 40.0 weight % of calcium. This reagent was stored at room temperature for up to six months with reducing activity in an air-tight glass bottle. The same procedure was followed for the preparation of similar reagents containing different weight % of calcium in silica gel.

Caution: The entire procedure should be carefully performed under anhydrous condition and argon atmosphere in hood; otherwise, fire might result.

The Standard Procedure 1 for the Synthesis of Cyclohexenones 3. A reaction flask was charged with Ca@SiO_2 (containing Ca 40.0 wt %, 6.0–6.2 equiv) under a nitrogen atmosphere. To this reaction mass was added 2-MeTHF (2.0–3.0 mL) via syringe at room temperature. Then a solution of an aldehyde **1** (1.0 equiv) and a ketone **2** (12 equiv) in 2-MeTHF (0.50–1.0 mL) was injected into the reaction flask. After the reaction mixture was stirred at 25 °C for 48–72 h, the inorganic residue was filtered and washed with Et_2O (3 × 3.0 mL). The combined filtrates were then directly purified by use of column chromatography on silica gel with diethyl ether in hexanes as the eluent to give the desired cyclohexenone **3**.

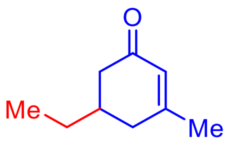

3aa

5-Ethyl-3-methyl-2-cyclohexen-1-one (3aa). The standard procedure 1 was followed by use of Ca@SiO_2 (271 mg, 40.0 wt %, for Ca: 108 mg, 6.1 equiv) in 2-MeTHF (2.5 mL), propionaldehyde (**1a**, 25.6 mg, 0.441 mmol, 1.0 equiv), and acetone (**2a**, 0.39 mL, 5.31 mmol, 12 equiv) in 2-MeTHF (1.0 mL). After the reaction mixture was stirred at 25 °C for 72 h, the crude product was purified by use of column chromatography (15% Et_2O in hexanes as the eluent) to give the desired cyclohexenone **3aa** (47.6 mg, 0.344 mmol) in 78% yield as a colorless oil: TLC R_f 0.42 (25% Et_2O in hexanes as the eluent); ^1H NMR ($CDCl_3$, 400 MHz) δ 5.84 (s, 1 H, =CHC=O), 2.44 (d, J = 14.8 Hz, 1 H, ½ × CH_2C=O), 2.29 (d, J = 14.8 Hz, 1 H, ½ × CH_2C=O), 2.03–1.95 (m, 3 H, CHEt + CH_2C=C), 1.93 (s, 3 H, CH_3), 1.42–1.35 (m, 2 H, CH_2CH_3), 0.90 (t, J = 7.6 Hz, 3 H, CH_2CH_3); HRMS (ESI-TOF) *m/z* $[M + Na]^+$ calcd for $C_9H_{14}O$ + Na 161.0942, found 161.0942. The spectroscopic data are in accordance with the literature data.[1]

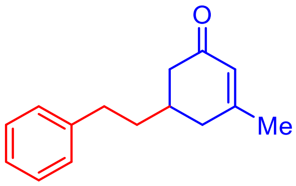

3ba

3-Methyl-5-phenethyl-2-cyclohexen-1-one (3ba). The standard procedure 1 was followed by use of Ca@SiO_2 (134 mg, 40.0 wt %, for Ca: 53.6 mg, 6.2 equiv) in 2-MeTHF (3.0 mL), 3-phenylpropionaldehyde (**1b**, 28.9 mg, 0.215 mmol, 1.0 equiv), and acetone (**2a**, 0.19 mL, 2.58 mmol, 12 equiv) in 2-MeTHF (1.0 mL). After the reaction mixture was stirred at 25 °C for 72 h, the crude product was purified by use of column chromatography (20% Et_2O in hexanes as the eluent) to give the desired cyclohexenone **3ba** (40.6 mg, 0.189 mmol) in 88% yield as a colorless oil: TLC R_f 0.34 (30% EtO_2 in hexanes as the eluent); ^{1}H NMR ($CDCl_3$, 400 MHz) δ 7.26 (t, J = 7.4 Hz, 2 H, 2 × ArH), 7.18–7.14 (m, 3 H, 3 × ArH), 5.85 (s, 1 H, =CHC=O), 2.66–2.61 (m, 2 H, CH_2Ph), 2.50 (d, J = 12.8 Hz, 1 H, ½ × CH_2C=O), 2.33 (d, J = 12.8 Hz, 1 H, ½ × CH_2C=O), 2.08–2.04 (m, 3 H, C*H*CH_2 + CH_2C=C), 1.93 (s, 3 H, CH_3), 1.71–1.66 (m, 2 H, C*H_2*CH_2Ph); HRMS (ESI-TOF) *m/z* $[M + Na]^+$ calcd for $C_{15}H_{18}O$ + Na 237.1255, found 237.1256. The spectroscopic data are in accordance with the literature data.[1]

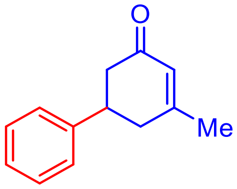

3ca

3-Methyl-5-phenyl-2-cyclohexen-1-one (3ca). The standard procedure 1 was followed by use of Ca@SiO_2 (144 mg, 40.0 wt %, for Ca: 57.6 mg, 6.0 equiv) in 2-MeTHF (2.0 mL), benzaldehyde (**1c**, 25.3 mg, 0.238 mmol, 1.0 equiv), and acetone (**2a**, 0.21 mL, 2.86 mmol, 12 equiv) in 2-MeTHF (0.50 mL). After the reaction mixture was stirred at 25 °C for 48 h, the crude product was purified by use of column chromatography (20% Et_2O in hexanes as the eluent) to give the desired cyclohexenone **3ca** (35.9 mg, 0.193 mmol) in 81% yield as colorless

solids: mp (recrystallized from ethanol) 36.1–38.2 °C; TLC R_f 0.43 (30% Et_2O in hexanes as the eluent); ^1H NMR ($CDCl_3$, 400 MHz) δ 7.34–7.21 (m, 5 H, 5 × ArH), 5.96 (s, 1 H, =CHC=O), 3.34–3.26 (m, 1 H, CHPh), 2.65–2.49 (m, 4 H, 2 × CH_2), 1.99 (s, 3 H, CH_3); HRMS (ESI-TOF) *m/z* $[M + Na]^+$ calcd for $C_{13}H_{14}O$ + Na 209.0942, found 209.0943. The spectroscopic data are in accordance with the literature data.[1]

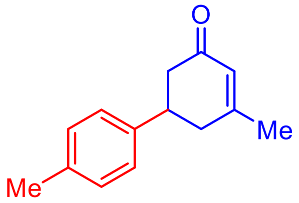

3fa

3-Methyl-5-(4-methylphenyl)-2-cyclohexen-1-one (3fa). The standard procedure 1 was followed by use of Ca@SiO_2 (153 mg, 40.0 wt %, for Ca: 61.2 mg, 6.1 equiv) in 2-MeTHF (2.0 mL), 4-methylbenzaldehyde (**1f**, 29.9 mg, 0.249 mmol, 1.0 equiv), and acetone (**2a**, 0.22 mL, 2.99 mmol, 12 equiv) in 2-MeTHF (0.50 mL). After the reaction mixture was stirred at 25 °C for 48 h, the crude product was purified by use of column chromatography (10% Et_2O in hexanes as the eluent) to give the desired cyclohexenone **3fa** (43.1 mg, 0.215 mmol) in 86% yield as a colorless oil: TLC R_f 0.44 (20% Et_2O in hexanes as the eluent); ^1H NMR ($CDCl_3$, 400 MHz) δ 7.14–7.10 (m, 4 H, 4 × ArH), 5.95 (s, 1 H, =CHC=O), 3.30–3.22 (m, 1 H, CHAr), 2.63–2.46 (m, 4 H, 2 × CH_2), 2.32 (s, 3 H, $PhCH_3$), 1.98 (s, 3 H, CH_3); HRMS (ESI-TOF) *m/z* $[M + H]^+$ calcd for $C_{14}H_{16}O$ + H 201.1279, found 201.1280. The spectroscopic data are in accordance with the literature data.[2]

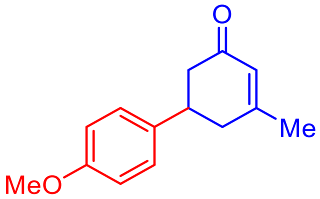

3ia

5-(4-Methoxyphenyl)-3-methyl-2-cyclohexen-1-one (3ia). The standard procedure 1 was followed by use of Ca@SiO_2 (132 mg, 40.0 wt %, for Ca: 52.8 mg, 6.2 equiv) in 2-MeTHF

(2.0 mL), 4-methoxybenzaldehyde (**1i**, 28.8 mg, 0.211 mmol, 1.0 equiv), and acetone (**2a**, 0.19 mL, 2.53 mmol, 12 equiv) in 2-MeTHF (1.0 mL). After the reaction mixture was stirred at 25 °C for 60 h, the crude product was purified by use of column chromatography (10% Et_2O in hexanes as the eluent) to give the desired cyclohexenone **3ia** (33.4 mg, 0.154 mmol) in 73% yield as a colorless oil: TLC R_f 0.39 (20% Et_2O in hexanes as the eluent); ^1H NMR ($CDCl_3$, 400 MHz) δ 7.13 (d, J = 8.6 Hz, 2 H, 2 × ArH), 6.85 (d, J = 8.6 Hz, 2 H, 2 × ArH), 5.94 (s, 1 H, =CHC=O), 3.77 (s, 3 H, OCH_3), 3.28–3.20 (m, 1 H, CHAr), 2.62–2.45 (m, 4 H, 2 × CH_2), 1.97 (s, 3 H, CH_3); HRMS (ESI-TOF) m/z $[M + H]^+$ calcd for $C_{14}H_{16}O_2$ + H 217.1228, found 217.1229. The spectroscopic data are in accordance with the literature data.[2]

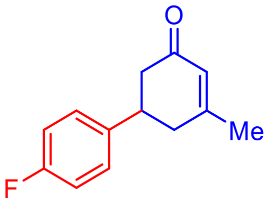

3la

5-(4-Fluorophenyl)-3-methyl-2-cyclohexen-1-one (3la). The standard procedure 1 was followed by use of Ca@SiO_2 (131 mg, 40.0 wt %, for Ca: 52.4 mg, 6.1 equiv) in 2-MeTHF (2.5 mL), 4-fluorobenzaldehyde (**1l**, 26.4 mg, 0.213 mmol, 1.0 equiv), and acetone (**2a**, 0.19 mL, 2.56 mmol, 12 equiv) in 2-MeTHF (0.50 mL). After the reaction mixture was stirred at 25 °C for 48 h, the crude product was purified by use of column chromatography (20% Et_2O in hexanes as the eluent) to give the desired cyclohexenone **3la** (37.8 mg, 0.185 mmol) in 87% yield as a colorless oil: TLC R_f 0.56 (30% Et_2O in hexanes as the eluent); ^1H NMR ($CDCl_3$, 400 MHz) δ 7.17 (dd, J = 8.6 Hz, 5.4 Hz, 2 H, 2 × ArH), 7.02–6.98 (m, 2 H, 2 × ArH), 5.95 (s, 1 H, =CHC=O), 3.32–3.24 (m, 1 H, CHAr), 2.62–2.46 (m, 4 H, 2 × CH_2), 1.98 (s, 3 H, CH_3); HRMS (ESI-TOF) m/z $[M + Na]^+$ calcd for $C_{13}H_{13}FO$ + Na 227.0848, found 227.0844. The spectroscopic data are in accordance with the literature data.[2]

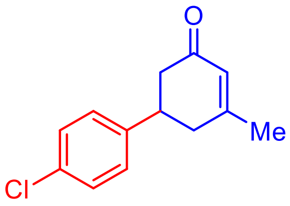

3oa

5-(4-Chlorophenyl)-3-methyl-2-cyclohexen-1-one (3oa). The standard procedure 1 was followed by use of Ca@SiO_2 (142 mg, 40.0 wt %, for Ca: 56.8 mg, 6.2 equiv) in 2-MeTHF (2.0 mL), 4-chlorobenzaldehyde (**1o**, 32.1 mg, 0.228 mmol, 1.0 equiv), and acetone (**2a**, 0.21 mL, 2.74 mmol, 12 equiv) in 2-MeTHF (1.0 mL). After the reaction mixture was stirred at 25 °C for 48 h, the crude product was purified by use of column chromatography (10% Et_2O in hexanes as the eluent) to give the desired cyclohexenone **3oa** (41.3 mg, 0.187 mmol) in 82% yield as a colorless oil: TLC R_f 0.49 (20% Et_2O in hexanes as the eluent); ^{1}H NMR ($CDCl_3$, 400 MHz) δ 7.28 (d, J = 8.4 Hz, 2 H, 2 × ArH), 7.14 (d, J = 8.4 Hz, 2 H, 2 × ArH), 5.95 (s, 1 H, =CHC=O), 3.32–3.24 (m, 1 H, CHAr), 2.62–2.44 (m, 4 H, 2 × CH_2), 1.98 (s, 3 H, CH_3); HRMS (ESI-TOF) m/z $[M + Na]^+$ calcd for $C_{13}H_{13}ClO$ + Na 243.0552, found 243.0550. The spectroscopic data are in accordance with the literature data.[1]

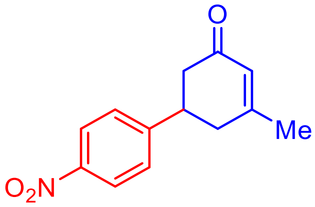

3ua

3-Methyl-5-(4-nitrophenyl)-2-cyclohexen-1-one (3ua). The standard procedure 1 was followed by use of Ca@SiO_2 (134 mg, 40.0 wt %, for Ca: 53.6 mg, 6.0 equiv) in 2-MeTHF (2.0 mL), 4-nitrobenzaldehyde (**1u**, 33.6 mg, 0.222 mmol, 1.0 equiv), and acetone (**2a**, 0.20 mL, 2.67 mmol, 12 equiv) in 2-MeTHF (1.0 mL). After the reaction mixture was stirred at 25 °C for 48 h, the crude product was purified by use of column chromatography (25% Et_2O in hexanes as the eluent) to give the desired cyclohexenone **3ua** (46.2 mg, 0.199 mmol) in 90%

yield as light yellow solids: mp (recrystallized from EtOH) 132.3–134.4 °C; TLC R_f 0.28 (40% Et_2O in hexanes as the eluent); ^1H NMR ($CDCl_3$, 400 MHz) δ 8.18 (d, J = 8.8 Hz, 2 H, 2 × ArH), 7.39 (d, J = 8.8 Hz, 2 H, 2 × ArH), 5.98 (s, 1 H, =CH–C=O), 3.47–3.39 (m, 1 H, CHAr), 2.66–2.50 (m, 4 H, 2 × CH_2), 2.00 (s, 3 H, CH_3); HRMS (ESI-TOF) m/z $[M + H]^+$ calcd for $C_{13}H_{13}NO_3$ + H 232.0973, found 232.0974. The spectroscopic data are in accordance with the literature data.[2]

Gram-Scale Synthesis of Compound 3ua.

The standard procedure 1 was followed by use of Ca@SiO_2 (4.1 g, 40.0 wt %, for Ca: 1.64 g, 6.0 equiv) in 2-MeTHF (65 mL), 4-nitrobenzaldehyde (**1u**, 1.03 g, 6.81 mmol, 1.0 equiv), and acetone (**2a**, 6.0 mL, 81.8 mmol, 12 equiv) in 2-MeTHF (35 mL). After the reaction mixture was stirred at 25 °C for 48 h, the crude product was purified by use of column chromatography (25% Et_2O in hexanes as the eluent) to give the desired cyclohexenone **3ua** (1.38 g, 5.96 mmol) in 87% isolated yield.

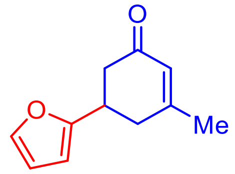

3xa

5-(2-Furanyl)-3-methyl-2-cyclohexen-1-one (3xa). The standard procedure 1 was followed by use of Ca@SiO_2 (146 mg, 40.0 wt %, for Ca: 58.4 mg, 6.2 equiv) in 2-MeTHF (3.0 mL), 2-furancarboxaldehyde (**1x**, 22.4 mg, 0.233 mmol, 1.0 equiv), and acetone (**2a**, 0.21 mL, 2.79 mmol, 12 equiv) in 2-MeTHF (0.50 mL). After the reaction mixture was stirred at 25 °C for 48 h, the crude product was purified by use of column chromatography (20% Et_2O in hexanes as the eluent) to give the desired cyclohexenone **3xa** (31.3 mg, 0.178 mmol) in 76% yield as a colorless oil: TLC R_f 0.34 (30% EtO_2 in hexanes as the eluent); ^1H NMR ($CDCl_3$, 400 MHz) δ 7.31 (d, J = 0.8 Hz, 1 H, furan), 6.27 (t, J = 2.4 Hz, 1 H, furan), 6.02 (d, J = 3.2 Hz, 1 H,

furan), 5.92 (s, 1 H, =CHC=O), 3.44–3.36 (m, 1 H, CH–furan), 2.72–2.47 (m, 4 H, 2 × CH_2), 1.98 (s, 3 H, CH_3); HRMS (ESI-TOF) *m/z* $[M + Na]^+$ calcd for $C_{11}H_{12}O_2$ + Na 199.0735, found 199.0735. The spectroscopic data are in accordance with the literature data.[1]

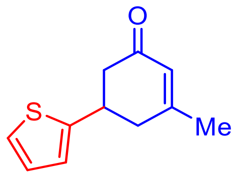

3ya

3-Methyl-5-(2-thienyl)-2-cyclohexen-1-one (3ya). The standard procedure 1 was followed by use of Ca@SiO_2 (139 mg, 40.0 wt %, for Ca: 55.6 mg, 6.1 equiv) in 2-MeTHF (2.5 mL), 2-thiophenecarboxaldehyde (**1y**, 25.5 mg, 0.227 mmol, 1.0 equiv), and acetone (**2a**, 0.20 mL, 2.72 mmol, 12 equiv) in 2-MeTHF (0.50 mL). After the reaction mixture was stirred at 25 °C for 48 h, the crude product was purified by use of column chromatography (25% Et_2O in hexanes as the eluent) to give the desired cyclohexenone **3ya** (34.5 mg, 0.179 mmol) in 79% yield as a colorless oil: TLC R_f 0.37 (35% Et_2O in hexanes as the eluent); ^1H NMR ($CDCl_3$, 400 MHz) δ 7.16 (d, J = 4.8 Hz, 1 H, thiophene), 6.93 (t, J = 4.0 Hz, 1 H, thiophene), 6.84 (d, J = 2.8 Hz, 1 H, thiophene), 5.95 (s, 1 H, =CHC=O), 3.63–3.56 (m, 1 H, CH–thiophene), 2.79–2.49 (m, 4 H, 2 × CH_2), 1.99 (s, 3 H, CH_3); HRMS (ESI-TOF) *m/z* $[M + Na]^+$ calcd for $C_{11}H_{12}OS$ + Na 215.0506, found 215.0511. The spectroscopic data are in accordance with the literature data.[2]

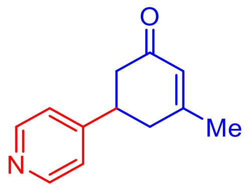

3za

3-Methyl-5-(4-pyridinyl)-2-cyclohexen-1-one (3za). The standard procedure 1 was followed by use of Ca@SiO_2 (147 mg, 40.0 wt %, for Ca: 58.8 mg, 6.2 equiv) in 2-MeTHF (2.5 mL), 4-pyridinecarboxaldehyde (**1z**, 25.3 mg, 0.236 mmol, 1.0 equiv), and acetone (**2a**, 0.21 mL,

2.83 mmol, 12 equiv) in 2-MeTHF (1.0 mL). After the reaction mixture was stirred at 25 °C for 48 h, the crude product was purified by use of column chromatography (40% EtOAc in hexanes as the eluent) to give the desired cyclohexenone **3za** (39.2 mg, 0.209 mmol) in 89% yield as a colorless oil: TLC R_f 0.39 (50% EtOAc in hexanes as the eluent); ^{1}H NMR ($CDCl_3$, 400 MHz) δ 8.50 (d, J = 5.6 Hz, 2 H, 2 × pyridine), 7.12 (d, J = 5.6 Hz, 2 H, 2 × pyridine), 5.93 (s, 1 H, =CHC=O), 3.32–3.24 (m, 1 H, CH–pyridine), 2.61–2.44 (m, 4 H, 2 × CH_2), 1.97 (s, 3 H, CH_3); HRMS (ESI-TOF) *m/z* $[M + H]^+$ calcd for $C_{12}H_{13}NO$ + H 188.1075, found 188.1070. The spectroscopic data are in accordance with the literature data.[3]

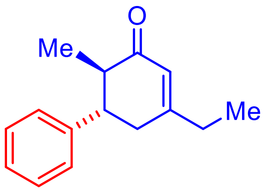

3cb

***trans*-3-Ethyl-6-methyl-5-phenyl-2-cyclohexen-1-one (3cb)**. The standard procedure 1 was followed by use of Ca@SiO_2 (226 mg, 40.0 wt %, for Ca: 90.4 mg, 6.0 equiv) in 2-MeTHF (2.0 mL), benzaldehyde (**1c**, 39.8 mg, 0.375 mmol, 1.0 equiv), and 2-butanone (**2b**, 75 µL, 0.825 mmol, 2.2 equiv) in 2-MeTHF (1.0 mL). After the reaction mixture was stirred at 25 °C for 72 h, the crude product was purified by use of column chromatography (20% Et_2O in hexanes as the eluent) to give the desired cyclohexenone **3cb** (60.3 mg, 0.281 mmol) in 75% yield as a colorless oil: TLC R_f 0.48 (40% Et_2O in hexanes as the eluent); ^{1}H NMR ($CDCl_3$, 400 MHz) δ 7.32 (t, J = 7.4 Hz, 2 H, 2 × ArH), 7.25–7.19 (m, 3 H, 3 × ArH), 5.94 (s, 1 H, =CHC=O), 2.93–2.86 (m, 1 H, CHPh), 2.63–2.52 (m, 2 H, CHMe + ½ × CH_2C=), 2.43 (dd, J = 18.2 Hz, 4.6 Hz, 1 H, ½ × CH_2C=), 2.22 (q, J = 7.2 Hz, 2 H, CH_2Me), 1.09 (t, J = 7.4 Hz, 3 H, CH_2CH_3), 0.91 (d, J = 6.8 Hz, 3 H, $CHCH_3$); HRMS (ESI-TOF) *m/z* $[M + Na]^+$ calcd for $C_{15}H_{18}O$ + Na 237.1255, found 237.1257. The spectroscopic data are in accordance with the literature data.[4]

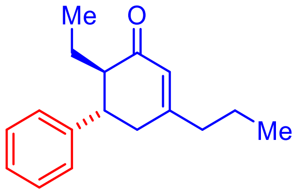

3cc

***trans*-6-Ethyl-5-phenyl-3-propyl-2-cyclohexen-1-one (3cc)**. The standard procedure 1 was followed by use of Ca@SiO_2 (206 mg, 40.0 wt %, for Ca: 82.4 mg, 6.1 equiv) in 2-MeTHF (2.0 mL), benzaldehyde (**1c**, 35.8 mg, 0.337 mmol, 1.0 equiv), and 2-pentanone (**2c**, 80.0 µL, 0.742 mmol, 2.2 equiv) in 2-MeTHF (1.0 mL). After the reaction mixture was stirred at 25 °C for 72 h, the residue was purified by use of column chromatography (20% Et_2O in hexanes as the eluent) to give the desired cyclohexenone **3cc** (59.7 mg, 0.246 mmol) in 73% yield as a colorless oil: TLC R_f 0.51 (40% Et_2O in hexanes as the eluent); ^{1}H NMR ($CDCl_3$, 400 MHz) δ 7.31 (t, J = 7.4 Hz, 2 H, 2 × ArH), 7.24–7.20 (m, 3 H, 3 × ArH), 5.92 (s, 1 H, =CHC=O), 3.18–3.11 (m, 1 H, CHPh), 2.58–2.46 (m, 3 H, CH_2C= + C*H*CH_2Me), 2.15 (t, J = 7.4 Hz, 2 H, =C$CH_2$$CH_2$Me), 1.81–1.73 (m, 1 H, ½ × COCHCH_2Me), 1.50 (sextet, J = 7.2 Hz, 2 H, CH_2CH_2Me), 1.35–1.25 (m, 1 H, ½ × COCHCH_2Me), 0.90 (t, J = 7.4 Hz, 3 H, COCHCH_2CH_3), 0.75 (t, J = 7.2 Hz, 3 H, CH_2CH_2CH_3); ^{13}C{^{1}H} NMR ($CDCl_3$, 100 MHz) δ 200.7 (C=O), 163.2, 143.1, 128.6, 127.4, 126.7, 125.8, 51.4, 44.5, 39.6, 38.5, 20.0, 19.5, 13.7 (CH_3), 10.1 (CH_3); IR (neat) 3028 (w), 2962 (m), 1667 (s, C=O), 1454 (m), 1377 (m), 1214 (m), 759 (m), 701 (m) cm^{-1}; HRMS (ESI-TOF) *m/z* [M + Na]$^+$ calcd for $C_{17}H_{22}O$ + Na 265.1568, found 265.1565.

The Standard Procedure 2 for the Synthesis of *α*-Keto Epoxides 5. A reaction flask was charged with Ca@SiO_2 (containing Ca 40.0 wt %, 2.0–2.2 equiv) under a nitrogen atmosphere. To this reaction mass was added 2-MeTHF (1.0–2.0 mL) via syringe at room temperature. Then a solution of aldehyde **1** (1.0 equiv) and α-bromoketone **4** (1.1 equiv) in 2-MeTHF (0.50–1.0 mL) was injected into the reaction flask. After the reaction mixture was stirred at 25 °C for 3.0–4.0 h, the inorganic residue was filtered and washed with EtOAc (3 × 3.0 mL). The

combined filtrates were concentrated under reduced pressure. The crude product was then purified by use of column chromatography on silica gel with EtOAc in hexanes as the eluent to give the desired *trans*-epoxide **5**.

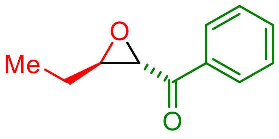

5aa

(3-Ethyloxiran-2-yl)phenylmethanone (5aa). The standard procedure 2 was followed by use of Ca@SiO_2 (81.3 mg, 40.0 wt %, for Ca: 32.5 mg, 2.2 equiv) in 2-MeTHF (1.5 mL), propionaldehyde (**1a**, 21.2 mg, 0.365 mmol, 1.0 equiv), and 2-bromoacetophenone (**4a**, 80.1 mg, 0.402 mmol, 1.1 equiv) in 2-MeTHF (1.0 mL). After the reaction mixture was stirred at 25 °C for 4.0 h, the crude product was purified by use of column chromatography (10% EtOAc in hexanes as the eluent) to give the desired *trans*-epoxide **5aa** (50.3 mg, 0.285 mmol) in 78% yield as a colorless oil: TLC R_f 0.33 (15% EtOAc in hexanes as the eluent); ^{1}H NMR ($CDCl_3$, 400 MHz) δ 7.99 (d, J = 7.6 Hz, 2 H, 2 × ArH), 7.60 (t, J = 7.4 Hz, 1 H, ArH), 7.48 (t, J = 7.6 Hz, 2 H, 2 × ArH), 4.01 (d, J = 1.6 Hz, 1 H, CHC=O), 3.14–3.11 (m, 1 H, CHEt), 1.82–1.73 (m, 2 H, CH_2), 1.07 (t, J = 7.6 Hz, 3 H, CH_3); HRMS (ESI-TOF) *m/z* $[M + Na]^+$ calcd for $C_{11}H_{12}O_2$ + Na 199.0735, found 199.0739. The spectroscopic data are in accordance with the literature data.[5]

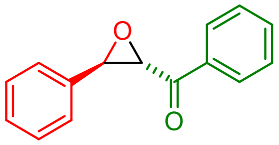

5ca

Phenyl(3-phenyl-2-oxiranyl)methanone (5ca). The standard procedure 2 was followed by use of Ca@SiO_2 (39.1 mg, 40.0 wt %, for Ca: 15.6 mg, 2.0 equiv) in 2-MeTHF (1.5 mL), benzaldehyde (**1c**, 20.3 mg, 0.191 mmol, 1.0 equiv), and 2-bromoacetophenone (**4a**, 42.1 mg,

0.211 mmol, 1.1 equiv) in 2-MeTHF (0.50 mL). After the reaction mixture was stirred at 25 °C for 4.0 h, the crude product was purified by use of column chromatography (7.0% EtOAc in hexanes as the eluent) to give the desired *trans*-epoxide **5ca** (37.1 mg, 0.165 mmol) in 86% yield as white solids: mp (recrystallized from ethanol) 86.7–88.8 °C; TLC R_f 0.31 (10% EtOAc in hexanes as the eluent); ^{1}H NMR ($CDCl_3$, 400 MHz) δ 7.99 (d, J = 7.6 Hz, 2 H, 2 × ArH), 7.60 (t, J = 7.6 Hz, 1 H, ArH), 7.47 (t, J = 7.8 Hz, 2 H, 2 × ArH), 7.41–7.35 (m, 5 H, 5 × ArH), 4.28 (d, J = 1.4 Hz, 1 H, CHC=O), 4.06 (d, J = 1.4 Hz, 1 H, CHPh); HRMS (ESI-TOF) *m/z* $[M + Na]^+$ calcd for $C_{15}H_{12}O_2$ + Na 247.0735, found 247.0733. The spectroscopic data are in accordance with the literature data.[6]

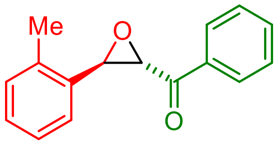

5da

[3-(2-Methylphenyl)oxiran-2-yl]phenylmethanone (5da). The standard procedure 2 was followed by use of Ca@SiO_2 (55.3 mg, 40.0 wt %, for Ca: 22.1 mg, 2.2 equiv) in 2-MeTHF (1.0 mL), 2-methylbenzaldehyde (**1d**, 29.8 mg, 0.248 mmol, 1.0 equiv), and 2-bromoacetophenone (**4a**, 54.4 mg, 0.273 mmol, 1.1 equiv) in 2-MeTHF (1.0 mL). After the reaction mixture was stirred at 25 °C for 4.0 h, the crude product was purified by use of column chromatography (5.0% EtOAc in hexanes as the eluent) to give the desired *trans*-epoxide **5da** (52.2 mg, 0.219 mmol) in 88% yield as pale yellow oil: TLC R_f 0.32 (10% EtOAc in hexanes as the eluent); ^{1}H NMR ($CDCl_3$, 400 MHz) δ 8.03 (d, J = 9.6 Hz, 2 H, 2 × ArH), 7.62 (t, J = 7.4 Hz, 1 H, ArH), 7.49 (t, J = 7.8 Hz, 2 H, 2 × ArH), 7.33–7.31 (m, 1 H, ArH), 7.26–7.23 (m, 2 H, 2 × ArH), 7.18–7.16 (m, 1 H, ArH), 4.21 (d, J = 1.6 Hz, 1 H, CHC=O), 4.20 (d, J = 1.6 Hz, 1 H, CHAr), 2.34 (s, 3 H, CH_3); HRMS (ESI-TOF) *m/z* $[M + Na]^+$ calcd for $C_{16}H_{14}O_2$ + Na 261.0891, found 261.0891. The spectroscopic data are in accordance with the literature data.[7]

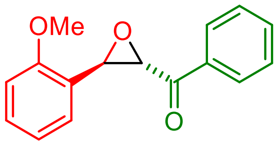

5ga

[3-(2-Methoxyphenyl)oxiran-2-yl]phenylmethanone (5ga). The standard procedure 2 was followed by use of Ca@SiO_2 (54.1 mg, 40.0 wt %, for Ca: 21.6 mg, 2.2 equiv) in 2-MeTHF (1.5 mL), 2-methoxybenzaldehyde (**1g**, 32.9 mg, 0.242 mmol, 1.0 equiv), and 2-bromoacetophenone (**4a**, 53.2 mg, 0.266 mmol, 1.1 equiv) in 2-MeTHF (1.0 mL). After the reaction mixture was stirred at 25 °C for 4.0 h, the crude product was purified by use of column chromatography (7.0% EtOAc in hexanes as the eluent) to give the desired *trans*-epoxide **5ga** (47.8 mg, 0.188 mmol) in 78% yield as white solids: mp (recrystallized from EtOAc/hexanes) 84.2–86.3 °C; TLC R_f 0.28 (10% EtOAc in hexanes as the eluent); ^{1}H NMR ($CDCl_3$, 400 MHz) δ 8.03 (d, J = 7.2 Hz, 2 H, 2 × ArH), 7.59 (t, J = 7.4 Hz, 1 H, ArH), 7.47 (t, J = 7.6 Hz, 2 H, 2 × ArH), 7.34–7.28 (m, 2 H, 2 × ArH), 6.98 (t, J = 7.4 Hz, 1 H, ArH), 6.90 (d, J = 8.4 Hz, 1 H, ArH), 4.37 (d, J = 1.6 Hz, 1 H, CHC=O), 4.17 (d, J = 1.6 Hz, 1 H, CHAr), 3.81 (s, 3 H, OCH_3); HRMS (ESI-TOF) *m/z* $[M + Na]^+$ calcd for $C_{16}H_{14}O_3$ + Na 277.0840, found 277.0833. The spectroscopic data are in accordance with the literature data.[8]

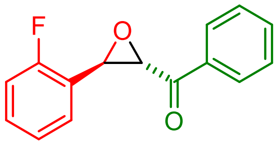

5ja

[3-(2-Fluorophenyl)oxiran-2-yl]phenylmethanone (5ja). The standard procedure 2 was followed by use of Ca@SiO_2 (54.2 mg, 40.0 wt %, for Ca: 21.7 mg, 2.2 equiv) in 2-MeTHF (2.0 mL), 2-fluorobenzaldehyde (**1j**, 30.2 mg, 0.243 mmol, 1.0 equiv), and 2-bromoacetophenone (**4a**, 53.3 mg, 0.267 mmol, 1.1 equiv) in 2-MeTHF (1.0 mL). After the reaction mixture was stirred at 25 °C for 3.0 h, the crude product was purified by use of column chromatography (10% EtOAc in hexanes as the eluent) to give the desired *trans*-epoxide **5ja**

(47.3 mg, 0.195 mmol) in 80% yield as a pale yellow oil: TLC R_f 0.53 (20% EtOAc in hexanes as the eluent); ^{1}H NMR ($CDCl_3$, 400 MHz) δ 8.01 (d, J = 7.2 Hz, 2 H, 2 × ArH), 7.61 (t, J = 7.6 Hz, 1 H, ArH), 7.48 (t, J = 7.6 Hz, 2 H, 2 × ArH), 7.36–7.30 (m, 2 H, 2 × ArH), 7.18 (t, J = 7.6 Hz, 1 H, ArH), 7.08 (t, J = 9.2 Hz, 1 H, ArH), 4.32 (d, J = 1.4 Hz, 1 H, CHC=O), 4.28 (d, J = 1.4 Hz, 1 H, CHAr); HRMS (ESI-TOF) m/z $[M + Na]^+$ calcd for $C_{15}H_{11}FO_2$ + Na 265.0640, found 265.0642. The spectroscopic data are in accordance with the literature data.[9]

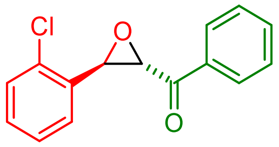

5ma

[3-(2-Chlorophenyl)oxiran-2-yl]phenylmethanone (5ma). The standard procedure 2 was followed by use of Ca@SiO_2 (51.6 mg, 40.0 wt %, for Ca: 20.6 mg, 2.1 equiv) in 2-MeTHF (1.5 mL), 2-chlorobenzaldehyde (**1m**, 34.2 mg, 0.243 mmol, 1.0 equiv), and 2-bromoacetophenone (**4a**, 53.2 mg, 0.267 mmol, 1.1 equiv) in 2-MeTHF (1.0 mL). After the reaction mixture was stirred at 25 °C for 4.0 h, the crude product was purified by use of column chromatography (5.0% EtOAc in hexanes as the eluent) to give the desired *trans*-epoxide **5ma** (55.4 mg, 0.214 mmol) in 88% yield as a pale yellow oil: TLC R_f 0.48 (10% EtOAc in hexanes as the eluent); ^{1}H NMR ($CDCl_3$, 400 MHz) δ 8.02 (d, J = 7.6 Hz, 2 H, 2 × ArH), 7.60 (t, J = 7.4 Hz, 1 H, ArH), 7.47 (t, J = 7.6 Hz, 2 H, 2 × ArH), 7.38–7.35 (m, 2 H, 2 × ArH), 7.31–7.28 (m, 2 H, 2 × ArH), 4.38 (d, J = 1.4 Hz, 1 H, CHC=O), 4.15 (d, J = 1.4 Hz, 1 H, CHAr); HRMS (ESI-TOF) m/z $[M + Na]^+$ calcd for $C_{15}H_{11}ClO_2$ + Na 281.0345, found 281.0339. The spectroscopic data are in accordance with the literature data.[7]

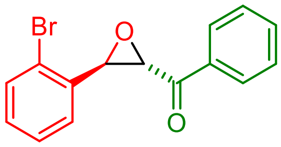

5pa

[3-(2-Bromophenyl)oxiran-2-yl]phenylmethanone (5pa). The standard procedure 2 was followed by use of Ca@SiO_2 (53.8 mg, 40.0 wt %, for Ca: 21.5 mg, 2.2 equiv) in 2-MeTHF (1.0 mL), 2-bromobenzaldehyde (**1p**, 44.8 mg, 0.242 mmol, 1.0 equiv), and 2-bromoacetophenone (**4a**, 53.1 mg, 0.266 mmol, 1.1 equiv) in 2-MeTHF (1.0 mL). After the reaction mixture was stirred at 25 °C for 4.0 h, the crude product was purified by use of column chromatography (5.0% EtOAc in hexanes as the eluent) to give the desired *trans*-epoxide **5pa** (59.8 mg, 0.197 mmol) in 81% yield as a pale yellow oil: TLC R_f 0.28 (10% EtOAc in hexanes as the eluent); ^{1}H NMR ($CDCl_3$, 400 MHz) δ 8.03 (d, J = 7.2 Hz, 2 H, 2 × ArH), 7.60 (t, J = 7.4 Hz, 1 H, ArH), 7.54 (d, J = 8.0 Hz, 1 H, ArH), 7.47 (t, J = 7.6 Hz, 2 H, 2 × ArH), 7.37–7.32 (m, 2 H, 2 × ArH), 7.23–7.19 (m, 1 H, ArH), 4.32 (d, J = 1.6 Hz, 1 H, CHC=O), 4.13 (d, J = 1.6 Hz, 1 H, CHAr); HRMS (ESI-TOF) *m/z* $[M + Na]^+$ calcd for $C_{15}H_{11}BrO_2$ + Na 326.9819, found 326.9805. The spectroscopic data are in accordance with the literature data.[7]

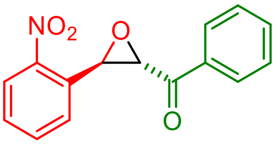

5sa

[3-(2-Nitrophenyl)oxiran-2-yl]phenylmethanone (5sa). The standard procedure 2 was followed by use of Ca@SiO_2 (51.8 mg, 40.0 wt %, for Ca: 20.7 mg, 2.1 equiv) in 2-MeTHF (1.0 mL), 2-nitrobenzaldehyde (**1s**, 36.6 mg, 0.242 mmol, 1.0 equiv), and 2-bromoacetophenone (**4a**, 52.9 mg, 0.266 mmol, 1.1 equiv) in 2-MeTHF (0.5 mL). After the reaction mixture was stirred at 25 °C for 3.5 h, the crude product was purified by use of column chromatography (15% EtOAc in hexanes as the eluent) to give *trans*-epoxide **5sa** (51.7 mg, 0.192 mmol) exclusively in 79% yield as white solids: mp (recrystallized from EtOAc/hexanes) 108.8–110.6 °C; TLC R_f 0.32 (30% EtOAc in hexanes as the eluent); ^{1}H NMR ($CDCl_3$, 400 MHz) δ 8.20 (d, J = 8.0 Hz, 1 H, ArH), 8.00 (d, J = 7.6 Hz, 2 H, 2 × ArH), 7.72 (d, J = 4.0 Hz, 2 H, 2 × ArH), 7.62–7.53 (m, 2 H, 2 × ArH), 7.47 (t, J = 7.8 Hz, 2 H, 2 × ArH), 4.62 (d, J = 1.8

Hz, 1 H, CHC=O), 4.20 (d, J = 1.8 Hz, 1 H, CHAr); HRMS (ESI-TOF) m/z [M + Na]$^+$ calcd for $C_{15}H_{11}NO_4$ + Na 292.0585, found 292.0582. The spectroscopic data are in accordance with the literature data.[10]

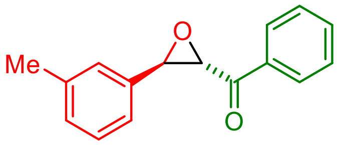

5ea

[3-(3-Methylphenyl)oxiran-2-yl]phenylmethanone (5ea). The standard procedure 2 was followed by use of Ca@SiO_2 (69.5 mg, 40.0 wt %, for Ca: 27.8 mg, 2.2 equiv) in 2-MeTHF (1.0 mL), 3-methylbenzaldehyde (**1e**, 37.4 mg, 0.311 mmol, 1.0 equiv), and 2-bromoacetophenone (**4a**, 68.1 mg, 0.342 mmol, 1.1 equiv) in 2-MeTHF (1.0 mL). After the reaction mixture was stirred at 25 °C for 4.0 h, the crude product was purified by use of column chromatography (5.0% EtOAc in hexanes as the eluent) to give the desired *trans*-epoxide **5ea** (63.2 mg, 0.265 mmol) in 85% yield as white solids: mp (recrystallized from EtOAc/hexanes) 42.3–44.4 °C; TLC R_f 0.35 (10% EtOAc in hexanes as the eluent); ^{1}H NMR ($CDCl_3$, 400 MHz) δ 7.99 (d, J = 7.6 Hz, 2 H, 2 × ArH), 7.60 (t, J = 7.4 Hz, 1 H, ArH), 7.47 (t, J = 7.8 Hz, 2 H, 2 × ArH), 7.27 (t, J = 7.8 Hz, 1 H, ArH), 7.17 (d, J = 7.6 Hz, 3 H, 3 × ArH), 4.28 (d, J = 1.6 Hz, 1 H, CHC=O), 4.02 (d, J = 1.6 Hz, 1 H, CHAr), 2.36 (s, 3 H, CH_3); HRMS (ESI-TOF) m/z [M + Na]$^+$ calcd for $C_{16}H_{14}O_2$ + Na 261.0891, found 261.0894. The spectroscopic data are in accordance with the literature data.[7]

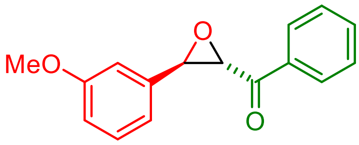

5ha

[3-(3-Methoxyphenyl)oxiran-2-yl]phenylmethanone (5ha). The standard procedure 2 was followed by use of Ca@SiO_2 (54.6 mg, 40.0 wt %, for Ca: 21.8 mg, 2.2 equiv) in 2-MeTHF (1.5 mL), 3-methoxybenzaldehyde (**1h**, 33.4 mg, 0.245 mmol, 1.0 equiv), and 2-

bromoacetophenone (**4a**, 53.7 mg, 0.269 mmol, 1.1 equiv) in 2-MeTHF (0.5 mL). After the reaction mixture was stirred at 25 °C for 3.5 h, the crude product was purified by use of column chromatography (10% EtOAc in hexanes as the eluent) to give the desired *trans*-epoxide **5ha** (47.9 mg, 0.188 mmol) in 77% yield as white solids: mp (recrystallized from EtOAc/hexanes) 79.2–81.3 °C; TLC R_f 0.31 (20% EtOAc in hexanes as the eluent); ^{1}H NMR ($CDCl_3$, 400 MHz) δ 7.98 (d, J = 7.2 Hz, 2 H, 2 × ArH), 7.60 (t, J = 7.4 Hz, 1 H, ArH), 7.47 (t, J = 7.8 Hz, 2 H, 2 × ArH), 7.29 (t, J = 7.8 Hz, 1 H, ArH), 6.95 (d, J = 7.6 Hz, 1 H, ArH), 6.91 (d, J = 2.0 Hz, 1 H, ArH), 6.88 (s, 1 H, ArH), 4.26 (d, J = 1.8 Hz, 1 H, CHC=O), 4.04 (d, J = 1.8 Hz, 1 H, CHAr), 3.81 (s, 3 H, OCH_3); HRMS (ESI-TOF) m/z $[M + Na]^+$ calcd for $C_{16}H_{14}O_3$ + Na 277.0840, found 277.0839. The spectroscopic data are in accordance with the literature data.[11]

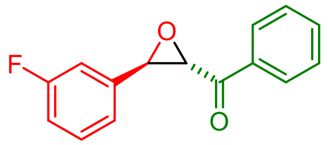

5ka

[3-(3-Fluorophenyl)oxiran-2-yl]phenylmethanone (5ka). The standard procedure 2 was followed by use of Ca@SiO_2 (52.2 mg, 40.0 wt %, for Ca: 20.9 mg, 2.1 equiv) in 2-MeTHF (1.0 mL), 3-fluorobenzaldehyde (**1k**, 30.3 mg, 0.244 mmol, 1.0 equiv), and 2-bromoacetophenone (**4a**, 53.4 mg, 0.268 mmol, 1.1 equiv) in 2-MeTHF (1.0 mL). After the reaction mixture was stirred at 25 °C for 4.0 h, the crude product was purified by use of column chromatography (5.0% EtOAc in hexanes as the eluent) to give the desired *trans*-epoxide **5ka** (49.2 mg, 0.203 mmol) in 83% yield as a pale yellow oil: TLC R_f 0.42 (10% EtOAc in hexanes as the eluent); ^{1}H NMR ($CDCl_3$, 400 MHz) δ 7.99 (d, J = 7.2 Hz, 2 H, 2 × ArH), 7.61 (t, J = 7.4 Hz, 1 H, ArH), 7.48 (t, J = 7.8 Hz, 2 H, 2 × ArH), 7.38–7.32 (m, 1 H, ArH), 7.15 (d, J = 7.6 Hz, 1 H, ArH), 7.07–7.03 (m, 2 H, 2 × ArH), 4.24 (d, J = 1.4 Hz, 1 H, CHC=O), 4.06 (d, J = 1.4 Hz, 1 H, CHAr); HRMS (ESI-TOF) m/z $[M + Na]^+$ calcd for $C_{15}H_{11}FO_2$ + Na 265.0640, found 265.0636. The spectroscopic data are in accordance with the literature data.[7]

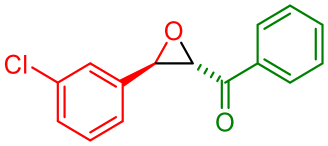

5na

[3-(3-Chlorophenyl)oxiran-2-yl]phenylmethanone (5na). The standard procedure 2 was followed by use of Ca@SiO_2 (53.8 mg, 40.0 wt %, for Ca: 21.5 mg, 2.2 equiv) in 2-MeTHF (1.0 mL), 3-chlorobenzaldehyde (**1n**, 33.9 mg, 0.241 mmol, 1.0 equiv), and 2-bromoacetophenone (**4a**, 52.8 mg, 0.265 mmol, 1.1 equiv) in 2-MeTHF (1.0 mL). After the reaction mixture was stirred at 25 °C for 4.0 h, the crude product was purified by use of column chromatography (15% EtOAc in hexanes as the eluent) to give the desired *trans*-epoxide **5na** (53.1 mg, 0.205 mmol) in 85% yield as a pale yellow oil: TLC R_f 0.51 (20% EtOAc in hexanes as the eluent); ^{1}H NMR ($CDCl_3$, 400 MHz) δ 7.98 (d, J = 7.2 Hz, 2 H, 2 × ArH), 7.61 (t, J = 7.4 Hz, 1 H, ArH), 7.48 (t, J = 7.8 Hz, 2 H, 2 × ArH), 7.33–7.29 (m, 3 H, 3 × ArH), 7.25–7.24 (m, 1 H, ArH), 4.24 (d, J = 1.2 Hz, 1 H, CHC=O), 4.04 (d, J = 1.2 Hz, 1 H, CHAr); HRMS (ESI-TOF) *m/z* $[M + Na]^+$ calcd for $C_{15}H_{11}ClO_2$ + Na 281.0345, found 281.0343. The spectroscopic data are in accordance with the literature data.[7]

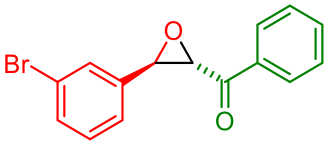

5qa

[3-(3-Bromophenyl)oxiran-2-yl]phenylmethanone (5qa). The standard procedure 2 was followed by use of Ca@SiO_2 (51.8 mg, 40.0 wt %, for Ca: 20.7 mg, 2.1 equiv) in 2-MeTHF (2.0 mL), 3-bromobenzaldehyde (**1q**, 45.2 mg, 0.244 mmol, 1.0 equiv), and 2-bromoacetophenone (**4a**, 53.4 mg, 0.268 mmol, 1.1 equiv) in 2-MeTHF (0.5 mL). After the reaction mixture was stirred at 25 °C for 4.0 h, the crude product was purified by use of column chromatography (5.0% EtOAc in hexanes as the eluent) to give *trans*-epoxide **5qa** (62.5 mg, 0.206 mmol) in 84% yield as a pale yellow oil: TLC R_f 0.41 (15% EtOAc in hexanes as the

eluent); ^{1}H NMR ($CDCl_3$, 400 MHz) δ 7.99–7.97 (m, 2 H, 2 × ArH), 7.61 (t, J = 7.2 Hz, 1 H, ArH), 7.50–7.46 (m, 4 H, 4 × ArH), 7.30–7.23 (m, 2 H, 2 × ArH), 4.24 (d, J = 1.8 Hz, 1 H, CHC=O), 4.03 (d, J = 1.8 Hz, 1 H, CHAr); HRMS (ESI-TOF) m/z $[M + Na]^+$ calcd for $C_{15}H_{11}BrO_2$ + Na 324.9840, found 324.9851. The spectroscopic data are in accordance with the literature data.[7]

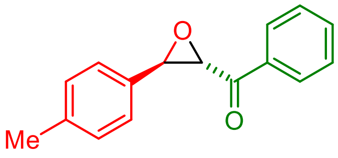

5fa

[3-(4-Methylphenyl)oxiran-2-yl]phenylmethanone (5fa). The standard procedure 2 was followed by use of Ca@SiO_2 (52.3 mg, 40.0 wt %, for Ca: 20.9 mg, 2.1 equiv) in 2-MeTHF (1.5 mL), 4-methylbenzaldehyde (**1f**, 29.2 mg, 0.243 mmol, 1.0 equiv), and 2-bromoacetophenone (**4a**, 53.2 mg, 0.267 mmol, 1.1 equiv) in 2-MeTHF (1.0 mL). After the reaction mixture was stirred at 25 °C for 3.0 h, the crude product was purified by use of column chromatography (10% EtOAc in hexanes as the eluent) to give the desired *trans*-epoxide **5fa** (50.6 mg, 0.212 mmol) in 87% yield as pale yellow oil: TLC R_f 0.60 (20% EtOAc in hexanes as the eluent); ^{1}H NMR ($CDCl_3$, 400 MHz) δ 7.98 (d, J = 8.0 Hz, 2 H, 2 × ArH), 7.60 (t, J = 7.6 Hz, 1 H, ArH), 7.48–7.44 (m, 2 H, 2 × ArH), 7.26–7.18 (m, 4 H, 4 × ArH), 4.28 (d, J = 1.8 Hz, 1 H, CHC=O), 4.02 (d, J = 1.8 Hz, 1 H, CHAr), 2.36 (s, 3 H, CH_3); HRMS (ESI-TOF) m/z $[M + H]^+$ calcd for $C_{16}H_{14}O_2$ + H 239.1072, found 239.1071. The spectroscopic data are in accordance with the literature data.[7]

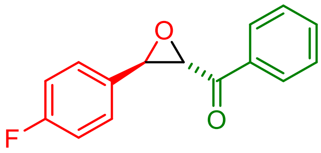

5la

[3-(4-Fluorophenyl)oxiran-2-yl]phenylmethanone (5la). The standard procedure 2 was followed by use of Ca@SiO_2 (54.3 mg, 40.0 wt %, for Ca: 21.7 mg, 2.2 equiv) in 2-MeTHF

(1.0 mL), 4-fluorobenzaldehyde (**1l**, 30.1 mg, 0.243 mmol, 1.0 equiv), and 2-bromoacetophenone (**4a**, 53.1 mg, 0.266 mmol, 1.1 equiv) in 2-MeTHF (1.0) mL. After the reaction mixture was stirred at 25 °C for 4.0 h, the crude product was purified by use of column chromatography (5.0% EtOAc in hexanes as the eluent) to give the desired *trans*-epoxide **5la** (50.7 mg, 0.209 mmol) in 86% yield as white solids: mp (recrystallized from EtOAc/hexanes) 92.2–94.2 °C; TLC R_f 0.28 (10% EtOAc in hexanes as the eluent); ^{1}H NMR ($CDCl_3$, 400 MHz) δ 7.98 (d, J = 7.2 Hz, 2 H, 2 × ArH), 7.60 (t, J = 7.4 Hz, 1 H, ArH), 7.47 (t, J = 7.6 Hz, 2 H, 2 × ArH), 7.34–7.31 (m, 2 H, 2 × ArH), 7.07 (t, J = 8.6 Hz, 2 H, 2 × ArH), 4.24 (d, J = 1.4 Hz, 1 H, CHC=O), 4.04 (d, J = 1.4 Hz, 1 H, CHAr); HRMS (ESI-TOF) m/z $[M + Na]^+$ calcd for $C_{15}H_{11}FO_2$ + Na 265.0640, found 265.0635. The spectroscopic data are in accordance with the literature data.[7]

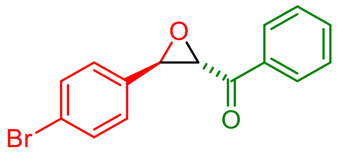

5ra

[3-(4-Bromophenyl)oxiran-2-yl]phenylmethanone (5ra). The standard procedure 2 was followed by use of Ca@SiO_2 (51.6 mg, 40.0 wt %, for Ca: 20.6 mg, 2.1 equiv) in 2-MeTHF (1.0 mL), 4-bromobenzaldehyde (**1r**, 44.6 mg, 0.241 mmol, 1.0 equiv), and 2-bromoacetophenone (**4a**, 52.8 mg, 0.265 mmol, 1.1 equiv) in 2-MeTHF (0.5 mL). After the reaction mixture was stirred at 25 °C for 3.5 h, the crude product was purified by use of column chromatography (10% EtOAc in hexanes as the eluent) to give *trans*-epoxide **5ra** (58.6 mg, 0.193 mmol) in 80% yield as a pale yellow oil: TLC R_f 0.37 (15% EtOAc in hexanes as the eluent); ^{1}H NMR ($CDCl_3$, 400 MHz) δ 7.97 (d, J = 7.2 Hz, 2 H, 2 × ArH), 7.61 (t, J = 7.6 Hz, 1 H, ArH), 7.52–7.46 (m, 4 H, 4 × ArH), 7.22 (d, J = 8.8 Hz, 2 H, 2 × ArH), 4.23 (d, J = 1.8 Hz, 1 H, CHC=O), 4.03 (d, J = 1.8 Hz, 1 H, CHAr); HRMS (ESI-TOF) m/z $[M + Na]^+$ calcd for $C_{15}H_{11}BrO_2$ + Na 324.9840, found 324.9836. The spectroscopic data are in accordance with the literature data.[7]

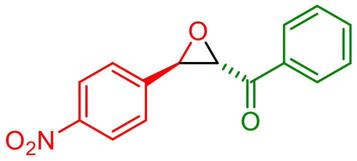

5ua

[3-(4-Nitrophenyl)oxiran-2-yl]phenylmethanone (5ua). The standard procedure 2 was followed by use of Ca@SiO_2 (51.8 mg, 40.0 wt %, for Ca: 20.7 mg, 2.1 equiv) in 2-MeTHF (1.0 mL), 4-nitrobenzaldehyde (**1u**, 36.9 mg, 0.244 mmol, 1.0 equiv), and 2-bromoacetophenone (**4a**, 53.5 mg, 0.268 mmol, 1.1 equiv) in 2-MeTHF (1.0 mL). After the reaction mixture was stirred at 25 °C for 4.0 h, the crude product was purified by use of column chromatography (25% EtOAc in hexanes as the eluent) to give the desired *trans*-epoxide **5ua** (57.4 mg, 0.213 mmol) in 87% yield as white solids: mp (recrystallized from EtOAc/hexanes) 148.8–150.9 °C; TLC R_f 0.30 (40% EtOAc in hexanes as the eluent); ^1H NMR ($CDCl_3$, 400 MHz) δ 8.25 (d, J = 8.8 Hz, 2 H, 2 × ArH), 7.99 (d, J = 7.6 Hz, 2 H, 2 × ArH), 7.63 (t, J = 7.4 Hz, 1 H, ArH), 7.55–7.47 (m, 4 H, 4 × ArH), 4.25 (d, J = 1.8 Hz, 1 H, CHC=O), 4.19 (d, J = 1.8 Hz, 1 H, CHAr); HRMS (ESI-TOF) *m/z* $[M + Na]^+$ calcd for $C_{15}H_{11}NO_4$ + Na 292.0585, found 292.0586. The spectroscopic data are in accordance with the literature data.[10]

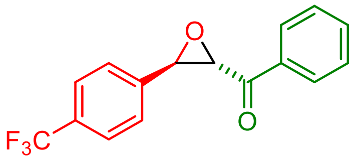

5va

Phenyl[3-[4-(trifluoromethyl)phenyl]oxiran-2-yl]methanone (5va). The standard procedure 2 was followed by use of Ca@SiO_2 (53.8 mg, 40.0 wt %, for Ca: 21.5 mg, 2.2 equiv) in 2-MeTHF (1.5 mL), 4-(trifluoromethyl)benzaldehyde (**1v**, 42.2 mg, 0.242 mmol, 1.0 equiv), and 2-bromoacetophenone (**4a**, 53.1 mg, 0.266 mmol, 1.1 equiv) in 2-MeTHF (1.0 mL). After the reaction mixture was stirred at 25 °C for 4.0 h, the crude product was purified by use of column chromatography (10% EtOAc in hexanes as the eluent) to give the desired *trans*-

epoxide **5va** (58.2 mg, 0.199 mmol) in 82% yield as a colorless oil: TLC R_f 0.42 (10% EtOAc in hexanes as the eluent); ^1H NMR ($CDCl_3$, 400 MHz) δ 7.98 (d, J = 6.8 Hz, 2 H, 2 × ArH), 7.66–7.60 (m, 3 H, 3 × ArH), 7.50–7.47 (m, 4 H, 4 × ArH), 4.25 (d, J = 1.6 Hz, 1 H, CHC=O), 4.13 (d, J = 1.6 Hz, 1 H, CHAr); HRMS (ESI-TOF) m/z $[M + Na]^+$ calcd for $C_{16}H_{11}F_3O_2$ + Na 315.0608, found 315.0605. The spectroscopic data are in accordance with the literature data.[8]

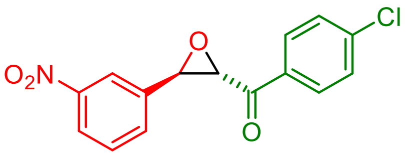

5td

(4-Chlorophenyl)[3-(3-nitrophenyl)oxiran-2-yl]methanone (5td). The standard procedure 2 was followed by use of Ca@SiO_2 (54.5 mg, 40.0 wt %, for Ca: 21.8 mg, 2.2 equiv) in 2-MeTHF (1.5 mL), 3-nitrobenzaldehyde (**1t**, 36.8 mg, 0.244 mmol, 1.0 equiv), and 2-bromo-4′-chloroacetophenone (**4d**, 62.7 mg, 0.268 mmol, 1.1 equiv). After the reaction mixture was stirred at 25 °C for 4.0 h, the crude product was purified by use of column chromatography (20% EtOAc in hexanes as the eluent) to give the desired *trans*-epoxide **5td** (61.4 mg, 0.202 mmol) in 83% yield as white solids: mp (recrystallized from EtOAc/hexanes) 127.2–128.8 °C; TLC R_f 0.42 (25% EtOAc in hexanes as the eluent); ^1H NMR ($CDCl_3$, 400 MHz) δ 8.22 (d, J = 8.4 Hz, 2 H, 2 × ArH), 7.95 (d, J = 8.4 Hz, 2 H, 2 × ArH), 7.69 (d, J = 7.6 Hz, 1 H, ArH), 7.58 (t, J = 7.6 Hz, 1 H, ArH), 7.47 (d, J = 8.4 Hz, 2 H, 2 × ArH), 4.22 (d, J = 1.4 Hz, 1 H, CHC=O), 4.19 (d, J = 1.4 Hz, 1 H, CHAr); HRMS (ESI-TOF) m/z $[M + Na]^+$ calcd for $C_{15}H_{10}ClNO_4$ + Na 326.0196, found 326.0188. The spectroscopic data are in accordance with the literature data.[12]

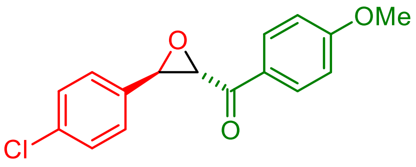

5ob

[3-(4-Chlorophenyl)oxiran-2-yl](4-methoxyphenyl)methanone (5ob). The standard procedure 2 was followed by use of Ca@SiO_2 (52.3 mg, 40.0 wt %, for Ca: 20.9 mg, 2.1 equiv) in 2-MeTHF (2.0 mL), 4-chlorobenzaldehyde (**1o**, 34.3 mg, 0.244 mmol, 1.0 equiv), and 2-bromo-4′-methoxyacetophenone (**4b**, 61.5 mg, 0.268 mmol, 1.1 equiv) in 2-MeTHF (1.0 mL). After the reaction mixture was stirred at 25 °C for 4.0 h, the crude product was purified by use of column chromatography (10% EtOAc in hexanes as the eluent) to give the desired *trans*-epoxide **5ob** (62.8 mg, 0.218 mmol) in 89% yield as a pale yellow oil: TLC R_f 0.31 (20% EtOAc in hexanes as the eluent); ^{1}H NMR ($CDCl_3$, 400 MHz) δ 7.98 (d, J = 8.8 Hz, 2 H, 2 × ArH), 7.35 (d, J = 8.4 Hz, 2 H, 2 × ArH), 7.27 (d, J = 8.4 Hz, 2 H, 2 × ArH) 6.93 (d, J = 9.2 Hz, 2 H, 2 × ArH), 4.18 (d, J = 1.8 Hz, 1 H, CHC=O), 4.03 (d, J = 1.8 Hz, 1 H, CHAr), 3.86 (s, 3 H, OCH_3); HRMS (ESI-TOF) *m/z* $[M + Na]^+$ calcd for $C_{16}H_{13}ClO_3$ + Na 311.0450, found 311.0448. The spectroscopic data are in accordance with the literature data.[7]

Gram-Scale Synthesis of 5ob.

The standard procedure 2 was followed by use of Ca@SiO_2 (1.63 g, 40.0 wt %, for Ca: 0.652 g, 2.1 equiv) in 2-MeTHF (45 mL), 4-chlorobenzaldehyde (**1o**, 1.06 g, 7.54 mmol, 1.0 equiv), and 2-bromo-4′-methoxyacetophenone (**4b**, 1.96 g, 8.52 mmol, 1.1 equiv) in 2-MeTHF (35 mL). After the reaction mixture was stirred at 25 °C for 4.0 h, the crude product was purified by use of column chromatography (10% EtOAc in hexanes as the eluent) to give the desired *trans*-epoxide **5ob** (1.88 g, 6.51 mmol) in 86% isolated yield.

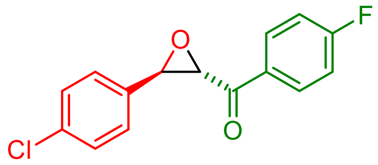

5oc

[3-(4-Chlorophenyl)oxiran-2-yl](4-fluorophenyl)methanone (5oc). The standard procedure 2 was followed by use of Ca@SiO_2 (54.2 mg, 40.0 wt %, for Ca: 21.7 mg, 2.2 equiv) in 2-MeTHF (1.5 mL), 4-chlorobenzaldehyde (**1o**, 34.2 mg, 0.243 mmol, 1.0 equiv), and 2-bromo-

4′-fluoroacetophenone (**4c**, 58.2 mg, 0.267 mmol, 1.1 equiv) in 2-MeTHF (1.0 mL). After the reaction mixture was stirred at 25 °C for 4.0 h, the crude product was purified by use of column chromatography (5.0% EtOAc in hexanes as the eluent) to give the desired *trans*-epoxide **5oc** (58.7 mg, 0.212 mmol) in 87% yield as a pale yellow oil: TLC R_f 0.40 (10% EtOAc in hexanes as the eluent); ^{1}H NMR ($CDCl_3$, 400 MHz) δ 8.05–8.01 (m, 2 H, 2 × ArH), 7.36 (d, J = 8.8 Hz, 2 H, 2 × ArH), 7.27 (d, J = 8.8 Hz, 2 H, 2 × ArH), 7.15 (t, J = 8.6 Hz, 2 H, 2 × ArH), 4.16 (d, J = 1.8 Hz, 1 H, CHC=O), 4.03 (d, J = 1.8 Hz, 1 H, CHAr); HRMS (ESI-TOF) *m/z* $[M + Na]^+$ calcd for $C_{15}H_{10}ClFO_2$ + Na 299.0251, found 299.0247. The spectroscopic data are in accordance with the literature data.[7]

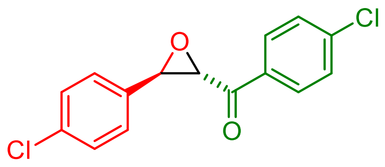

5od

(4-Chlorophenyl)[3-(4-chlorophenyl)oxiran-2-yl]methanone (5od). The standard procedure 2 was followed by use of Ca@SiO_2 (52.3 mg, 40.0 wt %, for Ca: 20.9 mg, 2.1 equiv) in 2-MeTHF (1.0 mL), 4-chlorobenzaldehyde (**1o**, 34.3 mg, 0.244 mmol, 1.0 equiv), and 2-bromo-4′-chloroacetophenone (**4d**, 62.7 mg, 0.268 mmol, 1.1 equiv) in 2-MeTHF (1.0 mL). After the reaction mixture was stirred at 25 °C for 4.0 h, the crude product was purified by use of column chromatography (10% EtOAc in hexanes as the eluent) to give the desired *trans*-epoxide **5od** (61.1 mg, 0.208 mmol) in 85% yield as white solids: mp (recrystallized from EtOH) 116.8–118.8 °C; TLC R_f 0.52 (20% EtOAc in hexanes as the eluent); ^{1}H NMR ($CDCl_3$, 400 MHz) δ 7.93 (d, J = 8.8 Hz, 2 H, 2 × ArH), 7.45 (d, J = 8.8 Hz, 2 H, 2 × ArH), 7.36 (d, J = 8.4 Hz, 2 H, 2 × ArH), 7.27 (d, J = 8.4 Hz, 2 H, 2 × ArH), 4.16 (d, J = 1.6 Hz, 1 H, CHC=O), 4.03 (d, J = 1.6 Hz, 1 H, CHAr); HRMS (ESI-TOF) *m/z* $[M + Na]^+$ calcd for $C_{15}H_{10}Cl_2O_2$ + Na 314.9955, found 314.9956. The spectroscopic data are in accordance with the literature data.[7]

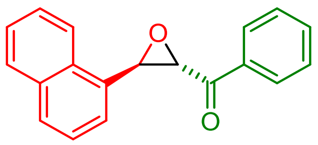

5wa

[3-(1-Naphthyl)oxiran-2-yl]phenylmethanone (5wa). The standard procedure 2 was followed by use of Ca@SiO_2 (54.2 mg, 40.0 wt %, for Ca: 21.7 mg, 2.2 equiv) in 2-MeTHF (2.0 mL), 1-naphthaldehyde (**1w**, 37.8 mg, 0.242 mmol, 1.0 equiv), and 2-bromoacetophenone (**4a**, 53.1 mg, 0.266 mmol, 1.1 equiv) in 2-MeTHF (1.0 mL). After the reaction mixture was stirred at 25 °C for 4.0 h, the crude product was purified by use of column chromatography (10% EtOAc in hexanes as the eluent) to give the desired *trans*-epoxide **5wa** (58.5 mg, 0.213 mmol) in 88% yield as a pale yellow oil: TLC R_f 0.47 (25% EtOAc in hexanes as the eluent); ^{1}H NMR ($CDCl_3$, 400 MHz) δ 8.06 (d, J = 6.8 Hz, 2 H, 2 × ArH), 7.97 (d, J = 7.6 Hz, 1 H, ArH) 7.90 (d, J = 7.2 Hz, 1 H, ArH), 7.86 (d, J = 8.4 Hz, 1 H, ArH), 7.64–7.59 (m, 2 H, 2 × ArH), 7.54–7.46 (m, 5 H, 5 × ArH), 4.71 (d, J = 1.6 Hz, 1 H, CHC=O), 4.30 (d, J = 1.6 Hz, 1 H, CHAr); HRMS (ESI-TOF) m/z $[M + H]^+$ calcd for $C_{19}H_{14}O_2$ + H 275.1072, found 275.1070. The spectroscopic data are in accordance with the literature data.[9]

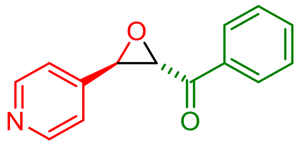

5za

Phenyl[3-(4-pyridinyl)oxiran-2-yl]methanone (5za). The standard procedure 2 was followed by use of Ca@SiO_2 (54.8 mg, 40.0 wt %, for Ca: 21.9 mg, 2.2 equiv) in 2-MeTHF (1.5 mL), 4-pyridinecarboxaldehyde (**1z**, 26.4 mg, 0.246 mmol, 1.0 equiv), and 2-bromoacetophenone (**4a**, 53.9 mg, 0.271 mmol, 1.1 equiv) in 2-MeTHF (1.0 mL). After the reaction mixture was stirred at 25 °C for 4.0 h, the crude product was purified by use of column chromatography (30% EtOAc in hexanes as the eluent) to give the desired *trans*-epoxide **5za** (43.3 mg, 0.192 mmol) in 78% yield as a colorless oil: TLC R_f 0.32 (50% EtOAc in hexanes

as the eluent); ^{1}H NMR ($CDCl_3$, 400 MHz) δ 8.63 (d, J = 6.0 Hz, 2 H, 2 × pyridine), 7.98 (d, J = 7.6 Hz, 2 H, 2 × ArH), 7.63 (t, J = 7.4 Hz, 1 H, ArH), 7.49 (t, J = 7.8 Hz, 2 H, 2 × ArH), 7.28 (d, J = 5.6 Hz, 2 H, 2 × pyridine), 4.24 (d, J = 1.4 Hz, 1 H, CHC=O), 4.07 (d, J = 1.4 Hz, 1 H, CH–pyridine); HRMS (ESI-TOF) m/z $[M + H]^+$ calcd for $C_{14}H_{11}NO_2$ + H 226.0868, found 226.0865. The spectroscopic data are in accordance with the literature data.[7]

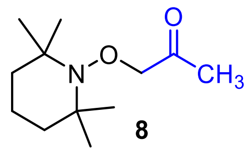

8

1-[(2,2,6,6-Tetramethylpiperidin-1-yl)oxy]propan-2-one (8). A reaction flask was charged with Ca@SiO_2 (145 mg, 40.0 wt %, for Ca: 58.1 mg, 6.1 equiv) under a nitrogen atmosphere. To this reaction mass was added 2-MeTHF (2.0 mL) via syringe at room temperature. Then a solution of benzaldehyde (**1c**, 25.2 mg, 0.237 mmol, 1.0 equiv), and acetone (**2a**, 0.21 mL, 2.85 mmol, 12 equiv) in 2-MeTHF (0.50 mL) was injected into the reaction flask. After the reaction mixture was stirred at 25 °C for 1.0 h, (2,2,6,6-tetramethylpiperidin-1-yl)oxyl (TEMPO) (74.1 mg, 0.474 mmol, 2.0 equiv) in 2-MeTHF (1.0 mL) was injected into the reaction flask. Then the reaction mixture was stirred at 25 °C for 48 h, the inorganic residue was filtered and washed with Et_2O (3 × 3.0 mL). The crude product was purified by use of column chromatography (5.0% Et_2O in pentane as the eluent) to give the desired TEMPO-acetone adduct **8** (14.2 mg, 66.6 µmol,) in 14% yield as a colorless oil: TLC R_f 0.68 (10% Et_2O in pentane as the eluent); ^{1}H NMR ($CDCl_3$, 400 MHz) δ 4.35 (s, 2 H, OCH_2), 2.18 (s, 3 H, CH_3C=O), 1.52–1.28 (m, 6 H, 3 × CH_2), 1.11 (d, J = 8.0 Hz, 12 H, 4 × CH_3C); HRMS (ESI-TOF) m/z $[M + Na]^+$ calcd for $C_{12}H_{23}NO_2$ + Na 236.1626, found 236.1628. The spectroscopic data are in accordance with the literature data.[13]

Table S1. Optimization of Reaction Conditions for 1c + 2a → 3ca in the Presence of Ca@SiO_2

entry	solvent	**2a** (equiv)	Ca (equiv)	yield of **3ca** (%)
1	2-MeTHF	8.0	5.0	60
2	2-MeTHF	9.0	5.0	64
3	2-MeTHF	10	5.0	68
4	2-MeTHF	11	5.0	68
5	2-MeTHF	12	5.0	72
6	2-MeTHF	12	6.0	81
7	2-MeTHF	13	6.0	81
8	2-MeTHF	14	6.0	81
9	2-MeTHF	12	7.0	80
10	2-MeTHF	13	7.0	78
11	2-MeTHF	14	7.0	78
12	2-MeTHF	12	8.0	76
13	2-MeTHF	13	8.0	75
14	2-MeTHF	14	8.0	75
15	THF	12	5.0	70
16	THF	12	6.0	79
17	THF	12	7.0	77
18	CH_3CN	12	5.0	68
19	CH_3CN	12	6.0	76
20	CH_3CN	12	7.0	76
21	Et_2O	12	6.0	0.0
22	Dioxane	12	6.0	0.0

1c (1.0 equiv), Ca@SiO_2 (40.0 wt %), solvent (3.0 mL), 48 h

Table S2. Optimization of Reaction Conditions for 1c + 4a → 5ca in the Presence of Different wt% of Ca in Ca@SiO_2

entry	solvent (2.0 mL)	Ca@SiO_2 (wt %)	yield (%)
1	THF	20.0	46
2	THF	30.2	59
3	THF	40.0	85
4	THF	50.1	61
5	2-MeTHF	20.0	48
6	2-MeTHF	30.2	60
7	2-MeTHF	40.0	86
8	2-MeTHF	50.1	63
9	2-MeTHF	60.0	42
10	CH_3CN	20.0	45
11	CH_3CN	30.2	58
12	CH_3CN	40.0	84
13	Et2O	40.0	0.0
14	Dioxane	40.0	0.0

1c (1.0 equiv), **4a** (1.1 equiv), Ca@SiO_2 (Ca 2.0 equiv), 4.0 h

Table S3. Optimization of Reaction Conditions for 1c + 4a → 5ca with Different Equivalents of Ca Metal in Ca@SiO_2

entry	solvent (2.0 mL)	Ca in Ca@SiO_2 (equiv)	yield (%)
1	THF	1.0	41
2	THF	1.5	62
3	THF	2.0	85
4	THF	2.5	84
5	2-MeTHF	1.0	42
6	2-MeTHF	1.5	64
7	2-MeTHF	2.0	86
8	2-MeTHF	2.5	85
9	CH_3CN	1.0	39
10	CH_3CN	1.5	61
11	CH_3CN	2.0	84
12	CH_3CN	2.5	84

1c (1.0 equiv), **4a** (1.1 equiv), Ca@SiO_2 (40.0 wt %), 4.0 h

References

(1) Zhou, J.; Wakchaure, V.; Kraft, P.; List, B. Primary-Amine-Catalyzed Enantioselective Intramolecular Aldolizations. *Angew. Chem., Int. Ed.* **2008**, *47*, 7656–7658.

(2) Wang, F.; Liu, Y.; Qi, Z.; Dai, W.; Li, X. Rhodium-Catalysed Tandem Aldol Condensation-Robinson Annulation Between Aldehydes and Acetone: Synthesis of 3-Methylcyclohexenones. *Tetrahedron Lett.* **2014**, *55*, 6399–6402.

(3) Cong, Z.; Wang, J.; Luo, B.; Li, X.; Cao, X.; Pan, Y.; Gu, H. The Synthesis of Cyclohexenone Using L-Proline Immobilized on a Silica Gel Catalyst by a Continuous-Flow Approach. *RSC Adv.* **2014**, *4*, 15036–15039.

(4) Okano, T.; Satou, Y.; Tamura, M.; Kiji, J. Aldol Reaction and Robinson-Type Annelation Catalyzed by Lanthanoid Triisopropoxides. *Bull. Chem. Soc. Jpn.* **1997**, *70*, 1879–1885.

(5) Li, B.; Li, C. Darzens Reaction Rate Enhancement Using Aqueous Media Leading to a High Level of Kinetically Controlled Diastereoselective Synthesis of Steroidal Epoxyketones. *J. Org. Chem.* **2014**, *79*, 8271–8277.

(6) Cussó, O.; Garcia-Bosch, I.; Ribas, X.; Lloret-Fillol, J.; Costas, M. Asymmetric Epoxidation with H_2O_2 by Manipulating the Electronic Properties of Non-heme Iron Catalysts. *J. Am. Chem. Soc.* **2013**, *135*, 14871–14878.

(7) Li, J.; Wang, D. Z. Visible-Light-Promoted Photoredox Syntheses of α,β-Epoxy Ketones from Styrenes and Benzaldehydes under Alkaline Conditions. *Org. Lett.* **2015**, *17*, 5260–5263.

(8) Qian, Q.; Tan, Y.; Zhao, B.; Feng, T.; Shen, Q.; Yao, Y. Asymmetric Epoxidation of Unsaturated Ketones Catalyzed by Heterobimetallic Rare Earth-Lithium Complexes Bearing Phenoxy-Functionalised Chiral Diphenylprolinolate Ligand. *Org. Lett.* **2014**, *16*, 4516–4519.

(9) Kapoor, M.; Hwu, J. R. Na@SiO_2-Mediated Addition of Organohalides to Carbonyl Compounds for the Formation of Alcohols and Epoxides. *Sci. Rep.* **2016**, *6*, 36225.

(10) Reddi, R. N.; Prasad, P. K.; Sudalai, A. N-Heterocyclic Carbene Catalysed Oxidative Coupling of Alkenes/α-Bromoacetophenones with Aldehydes: A Facile Entry to α,β-Epoxy Ketones. *Angew. Chem., Int. Ed.* **2015**, *54*, 14150–14153.

(11) Wu, Y.; Zhou, G.; Meng, Q.; Tang, X.; Liu, G.; Yin, H.; Zhao, J.; Yang, F.; Yu, Z.; Luo, Y. Visible Light-Induced Aerobic Epoxidation of α,β-Unsaturated Ketones Mediated by Amidines. *J. Org. Chem.* **2018**, *83*, 13051–13062.

(12) Ahmed, N.; Babu, B. V.; Kumar, H. Intramolecular Friedel-Crafts Alkylation of Chalcone Epoxides Using Indium(III) Chloride as an Efficient Catalyst. *Synthesis* **2011**, *2011*, 2471–2477.

(13) Li, Y.; Pouliot, M.; Vogler, T.; Renaud, P.; Studer, A. α-Aminoxylation of Ketones and β-Chloro-α-aminoxylation of Enones with TEMPO and Chlorocatecholborane. *Org. Lett.* **2012**, *14*, 4474–4477.

Spectra of Compounds

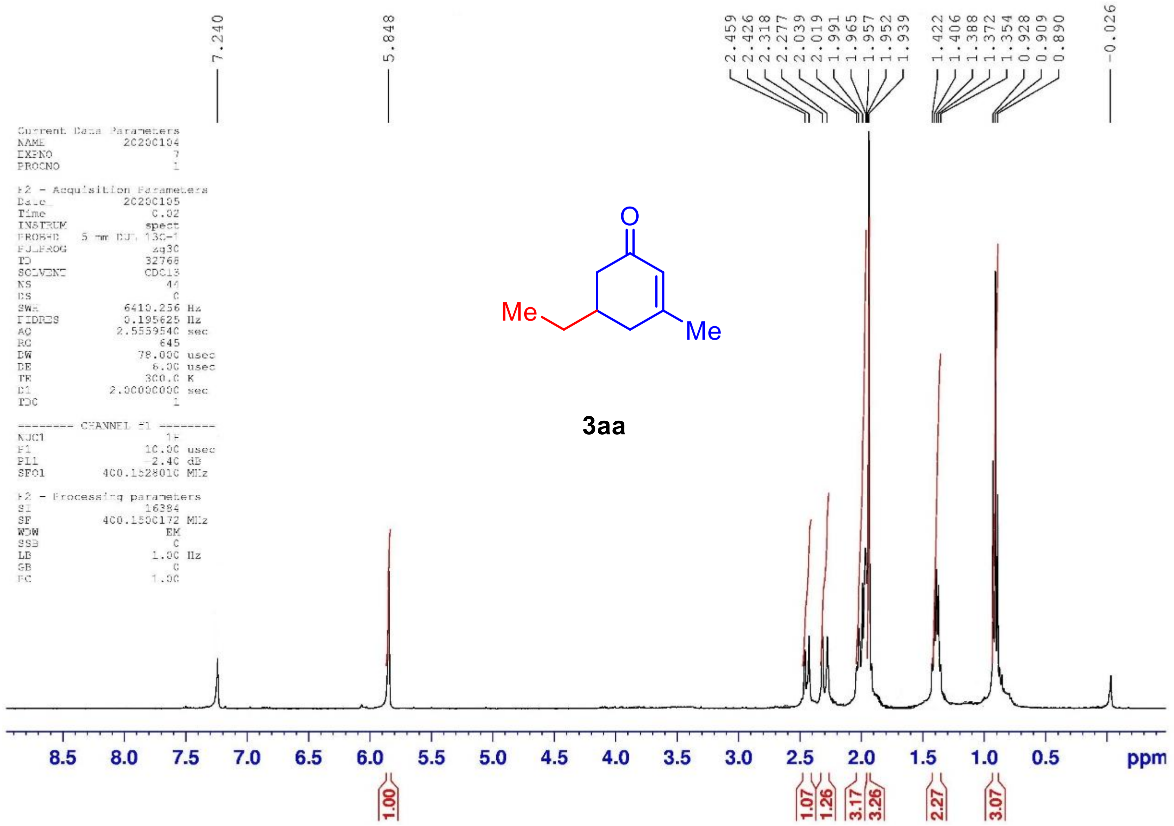

^{1}H NMR (400 MHz, $CDCl_3$) spectrum of compound **3aa**

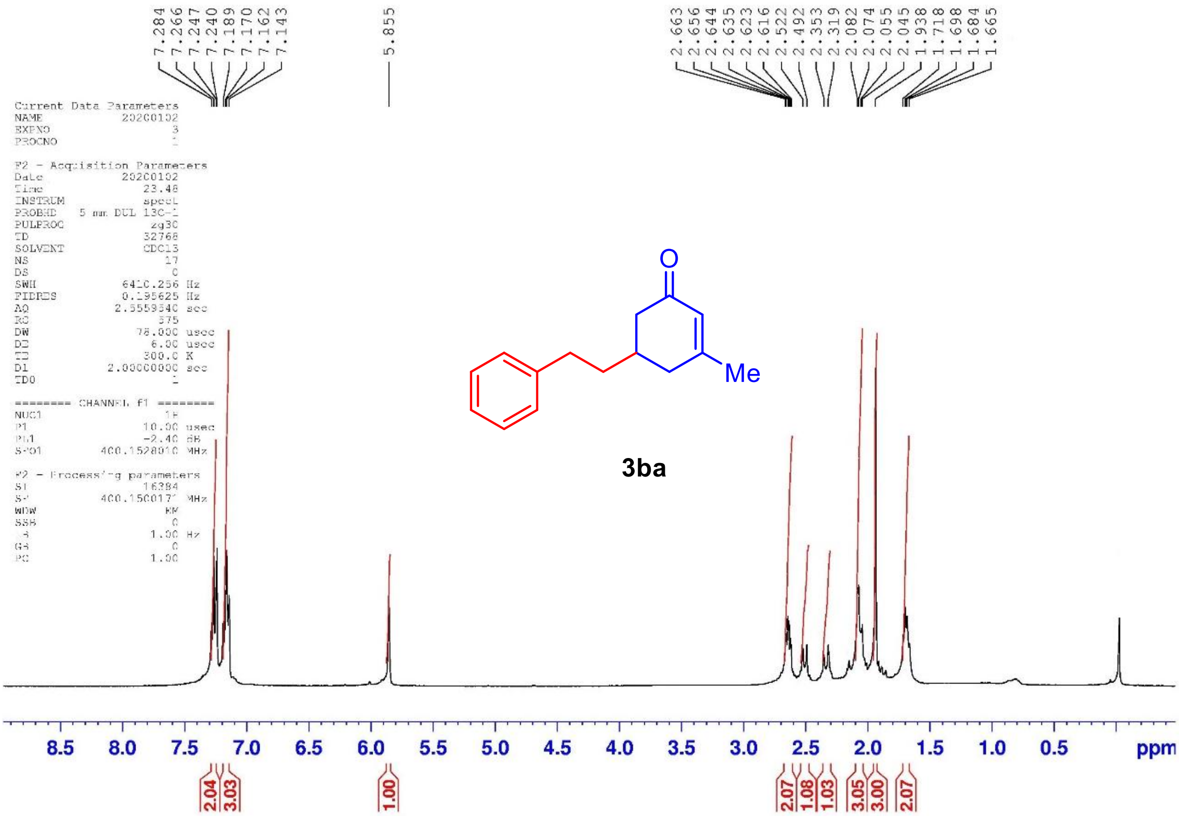

^{1}H NMR (400 MHz, $CDCl_3$) spectrum of compound **3ba**

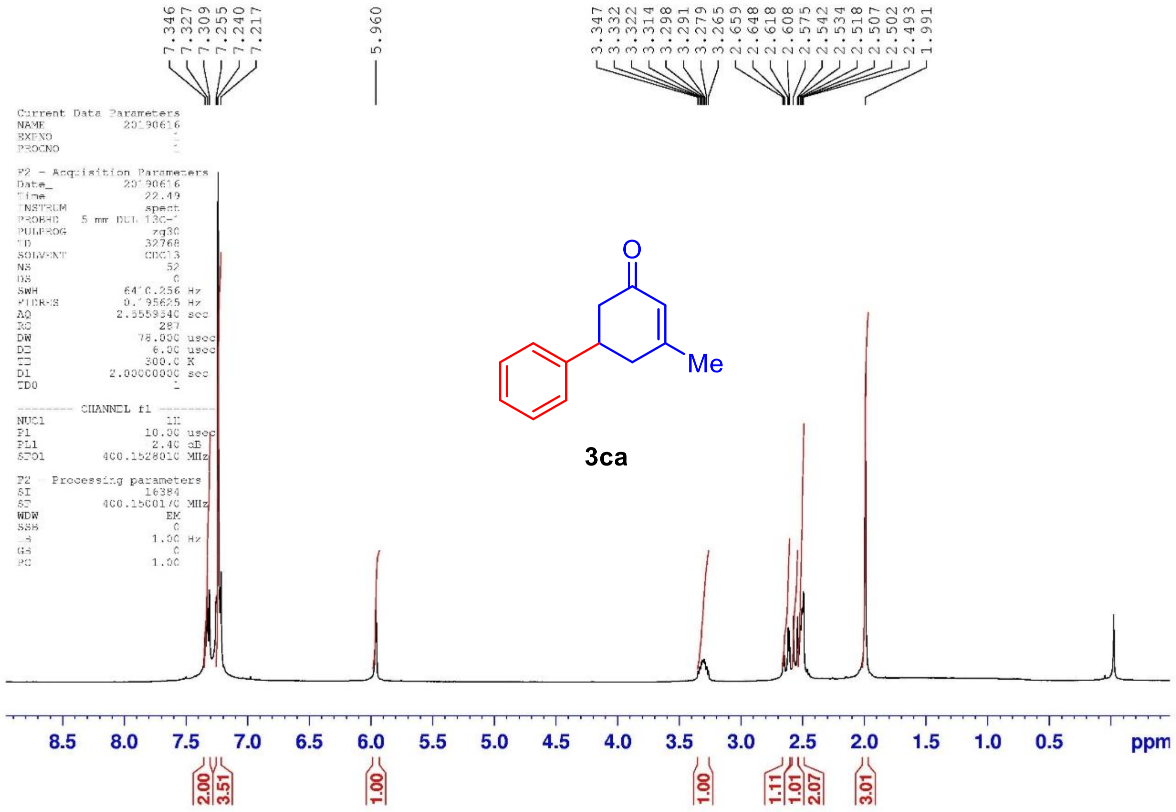

^1H NMR (400 MHz, $CDCl_3$) spectrum of compound **3ca**

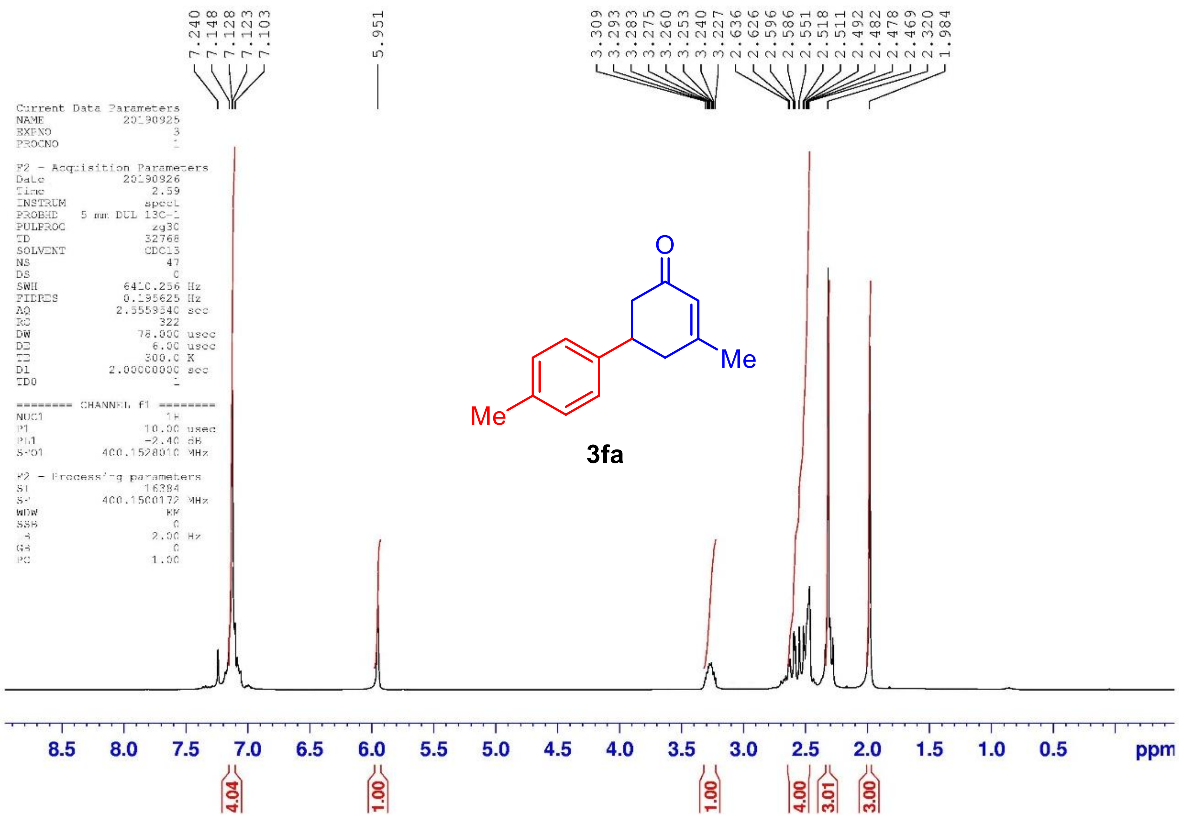

^1H NMR (400 MHz, $CDCl_3$) spectrum of compound **3fa**

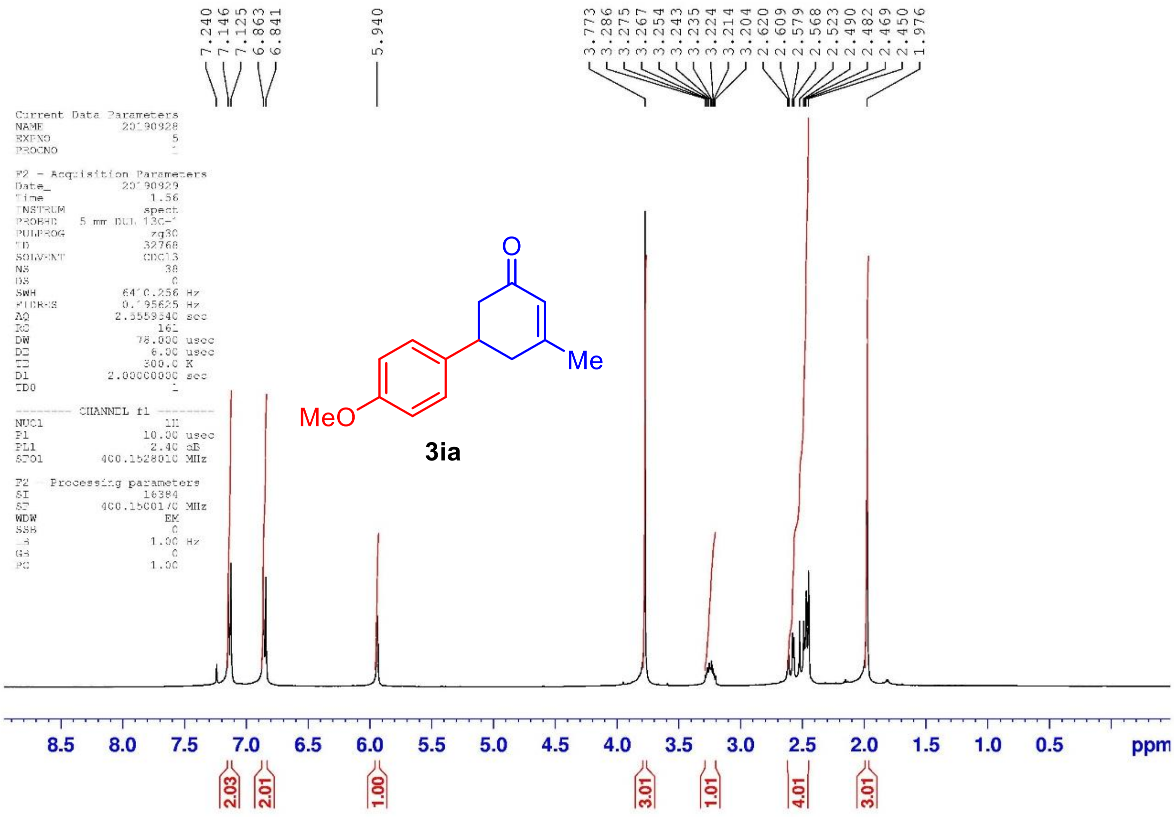

^{1}H NMR (400 MHz, CDCl$_3$) spectrum of compound **3ia**

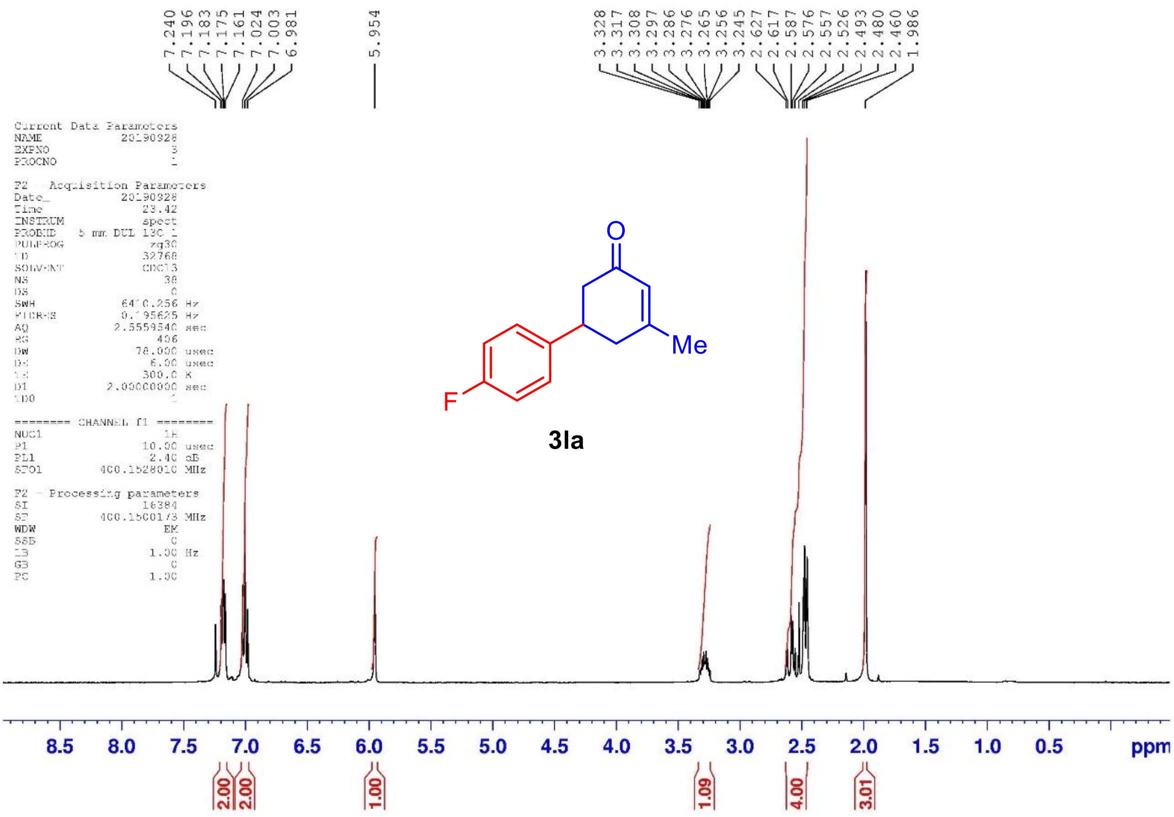

^{1}H NMR (400 MHz, CDCl$_3$) spectrum of compound **3la**

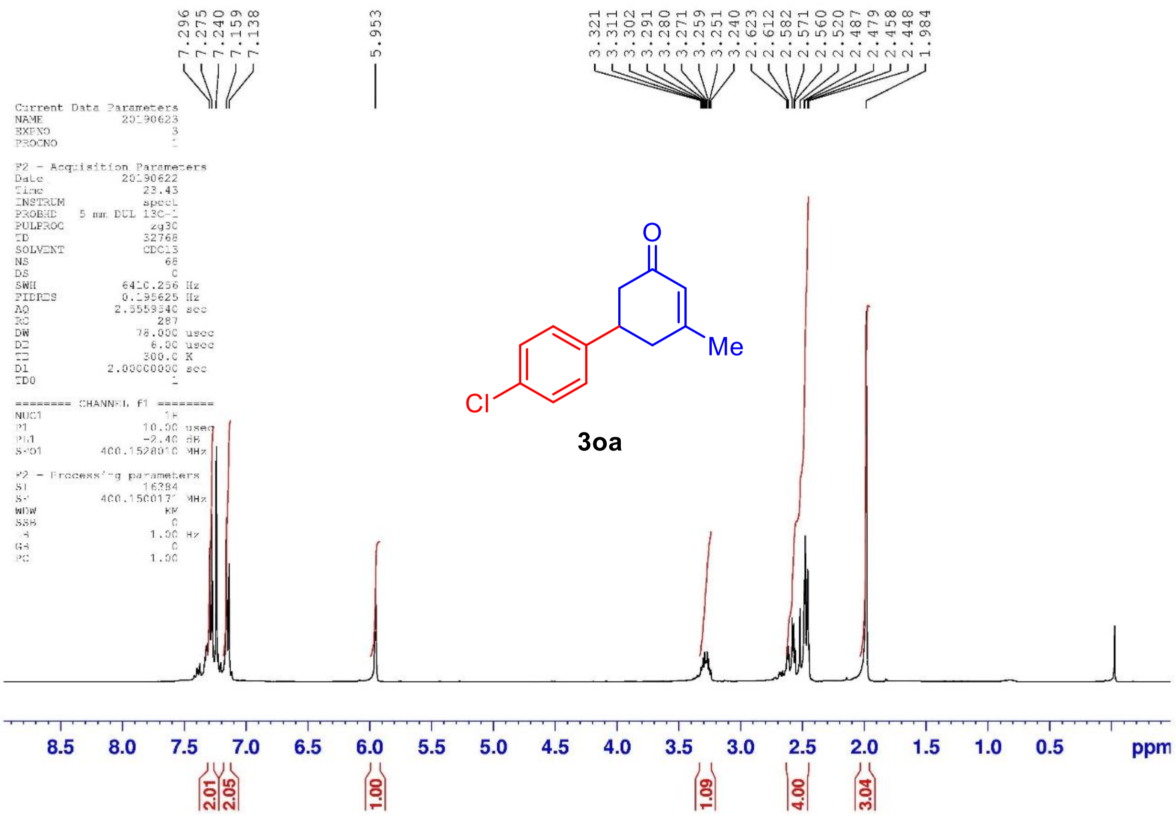

^{1}H NMR (400 MHz, $CDCl_3$) spectrum of compound **3oa**

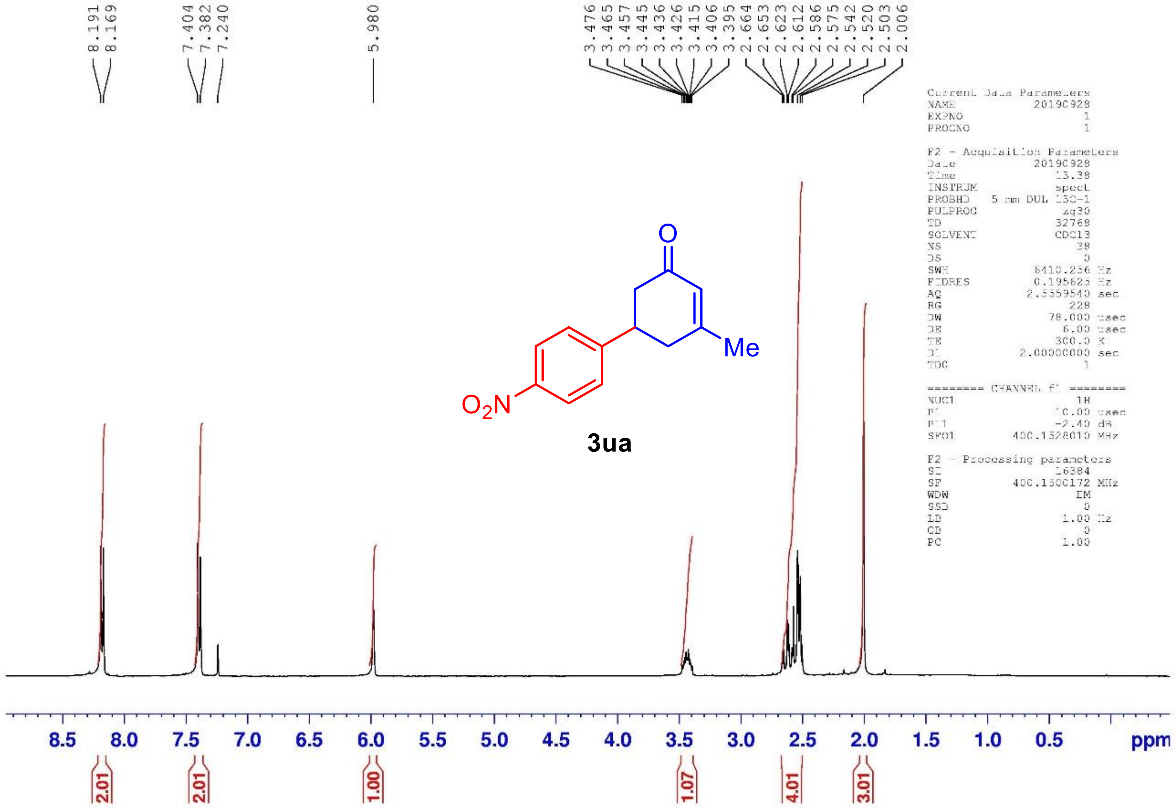

^{1}H NMR (400 MHz, $CDCl_3$) spectrum of compound **3ua**

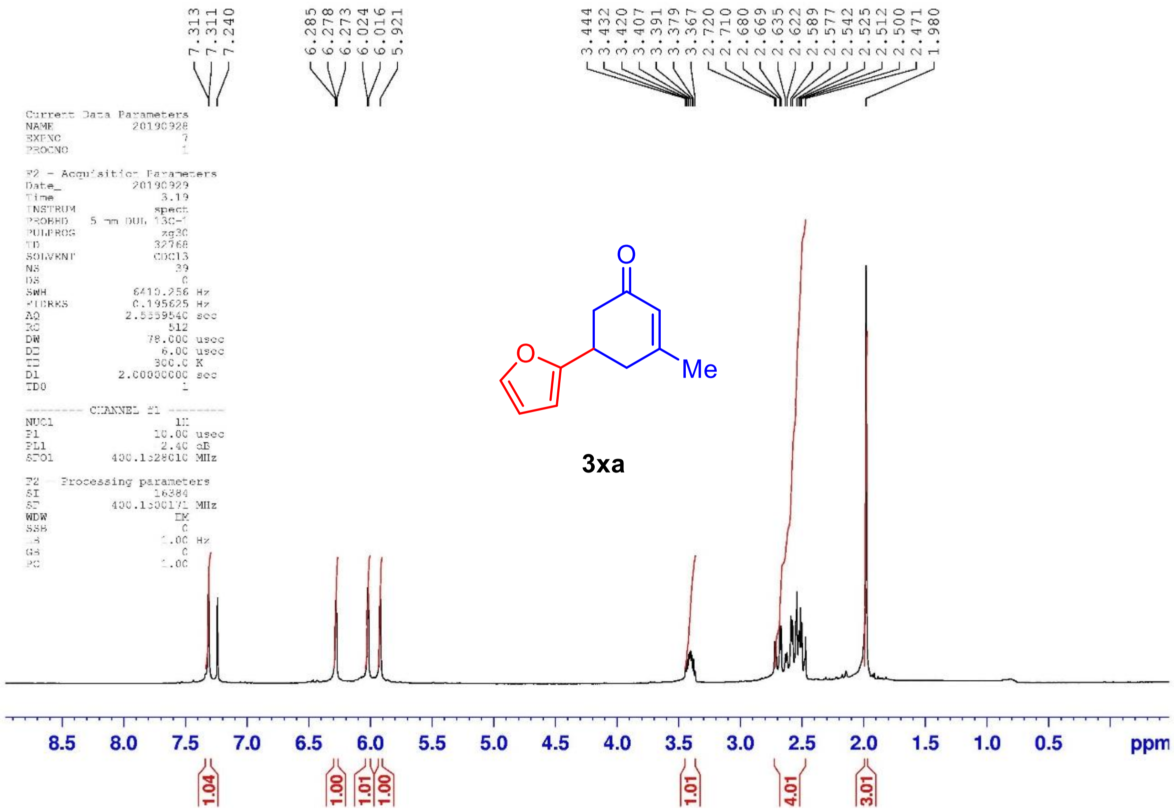

^{1}H NMR (400 MHz, CDCl$_3$) spectrum of compound **3xa**

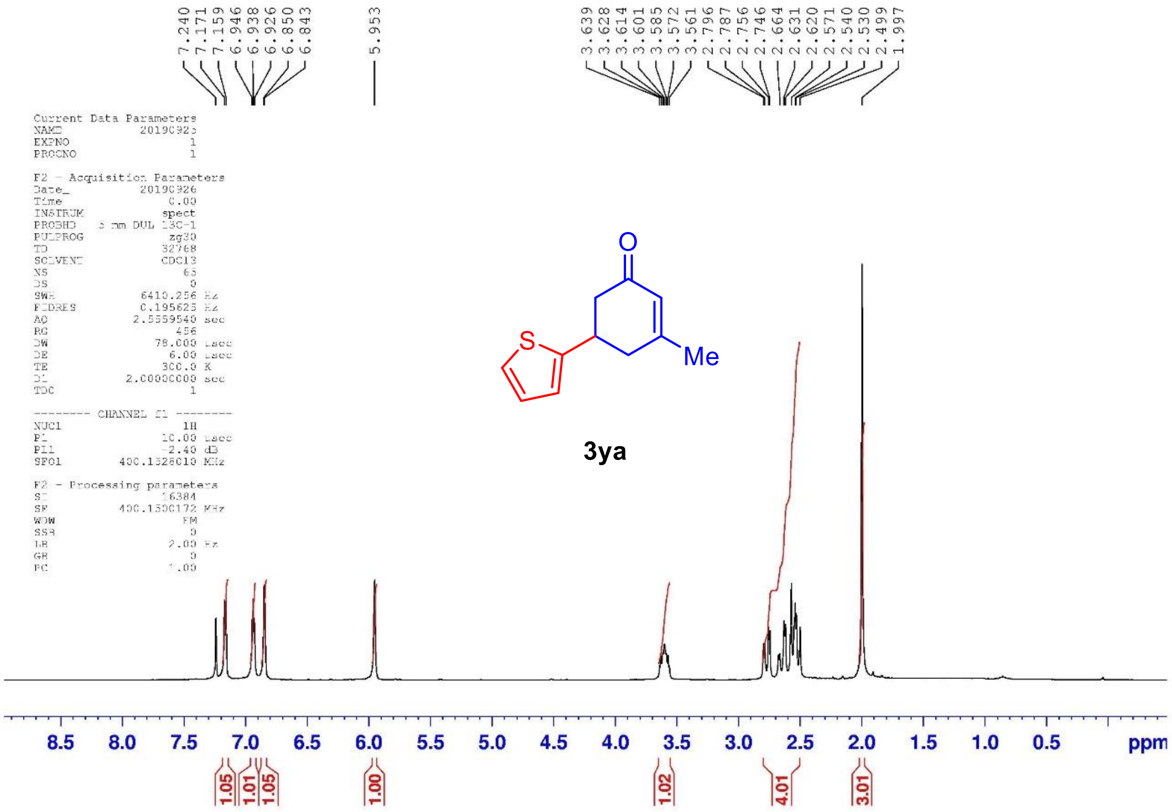

^{1}H NMR (400 MHz, CDCl$_3$) spectrum of compound **3ya**

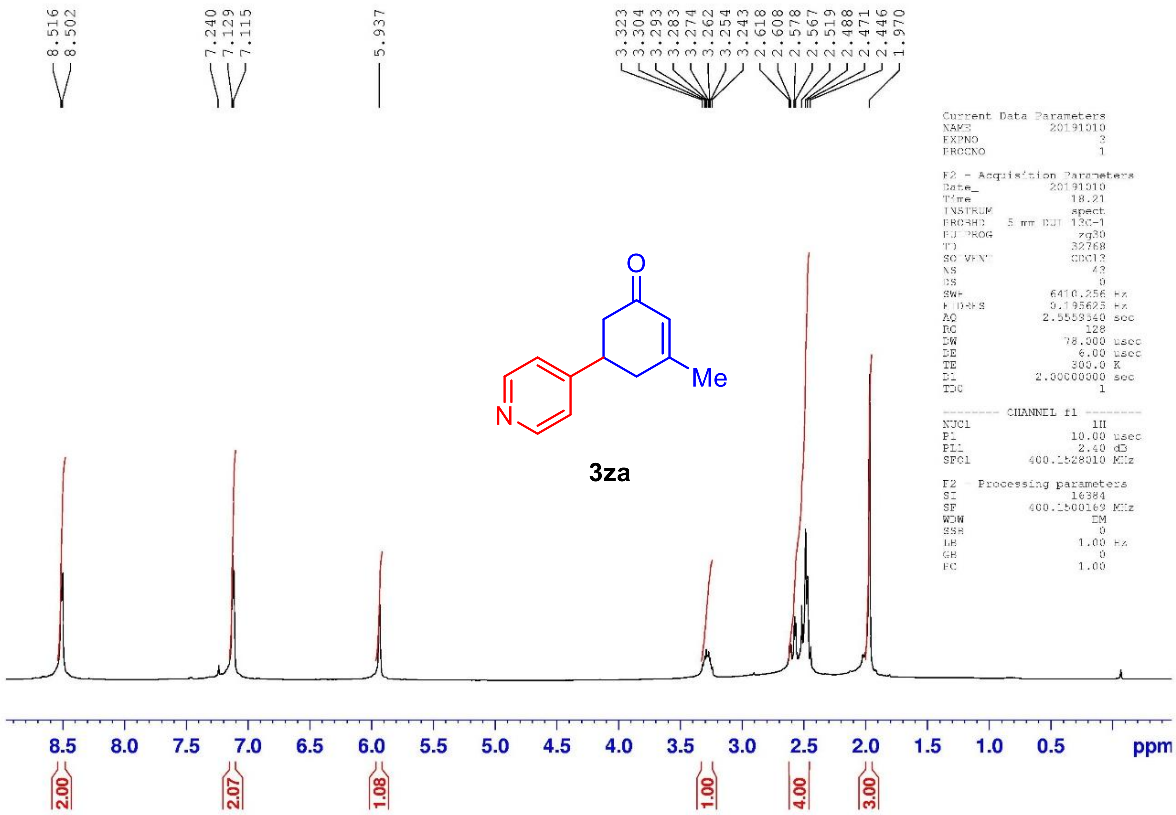

^{1}H NMR (400 MHz, $CDCl_3$) spectrum of compound **3za**

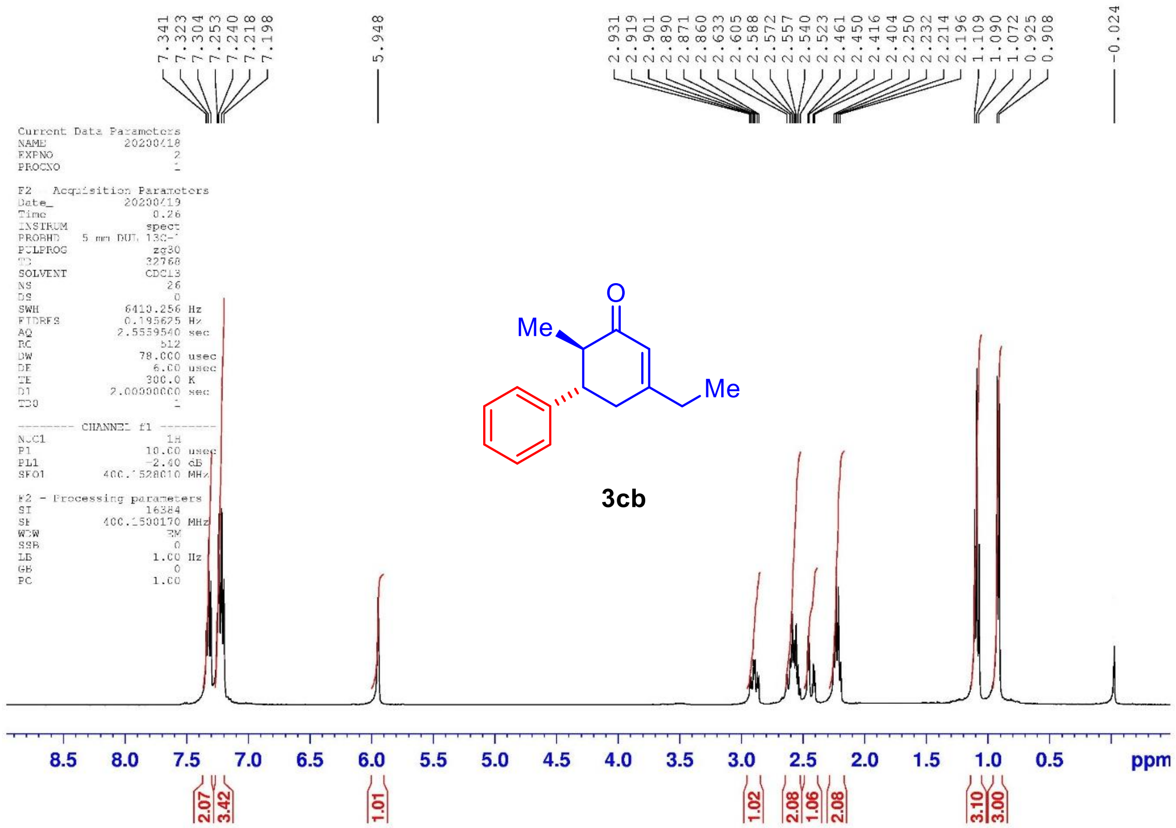

^{1}H NMR (400 MHz, $CDCl_3$) spectrum of compound **3cb**

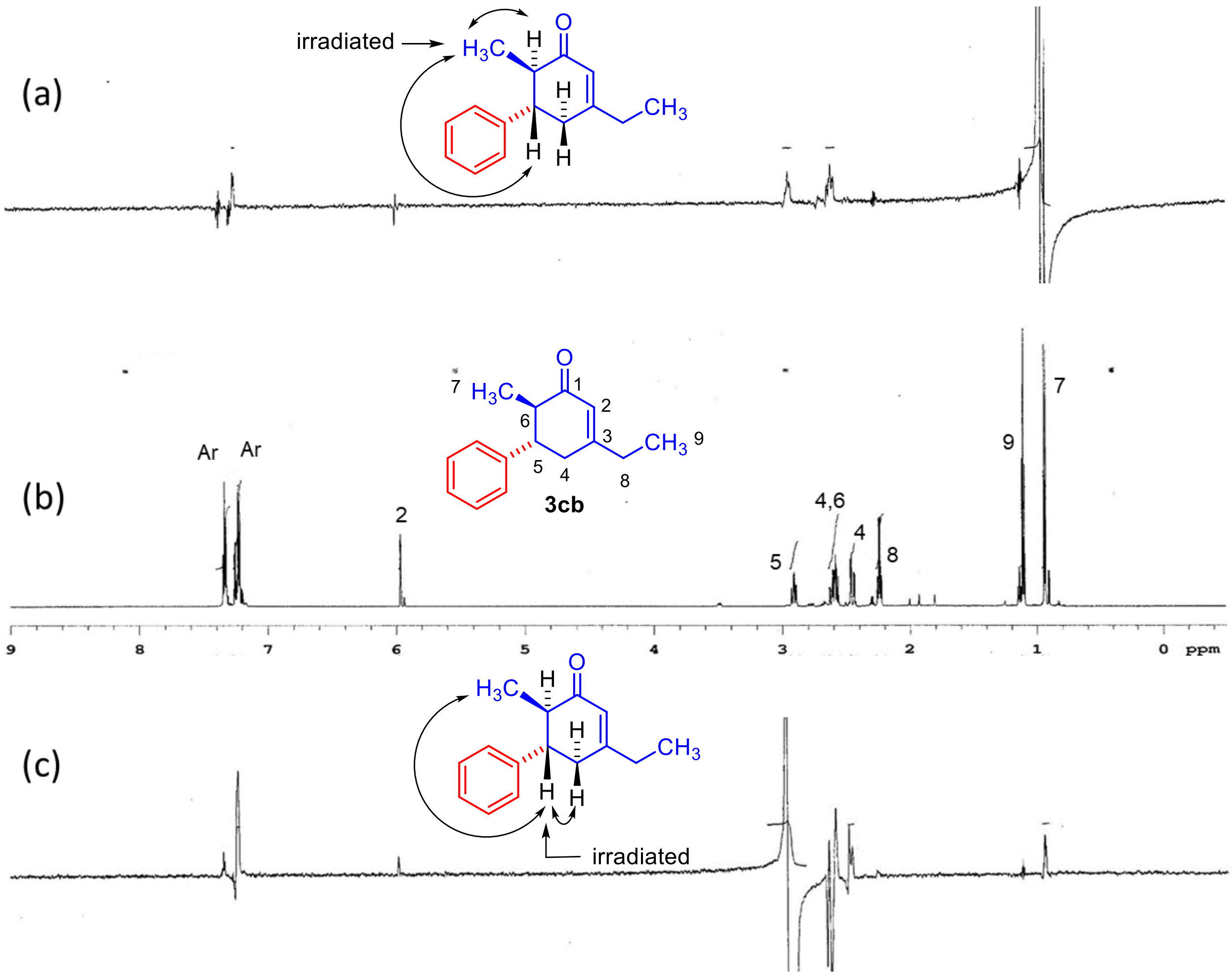

1D NOE (700 MHz, $CDCl_3$) Difference spectrum of compound **3cb** (a) 1D NOE with selective excitation at 0.93 ppm, (b) ^{1}H NMR spectrum, and (c) 1D NOE with selective excitation at 2.91 ppm.

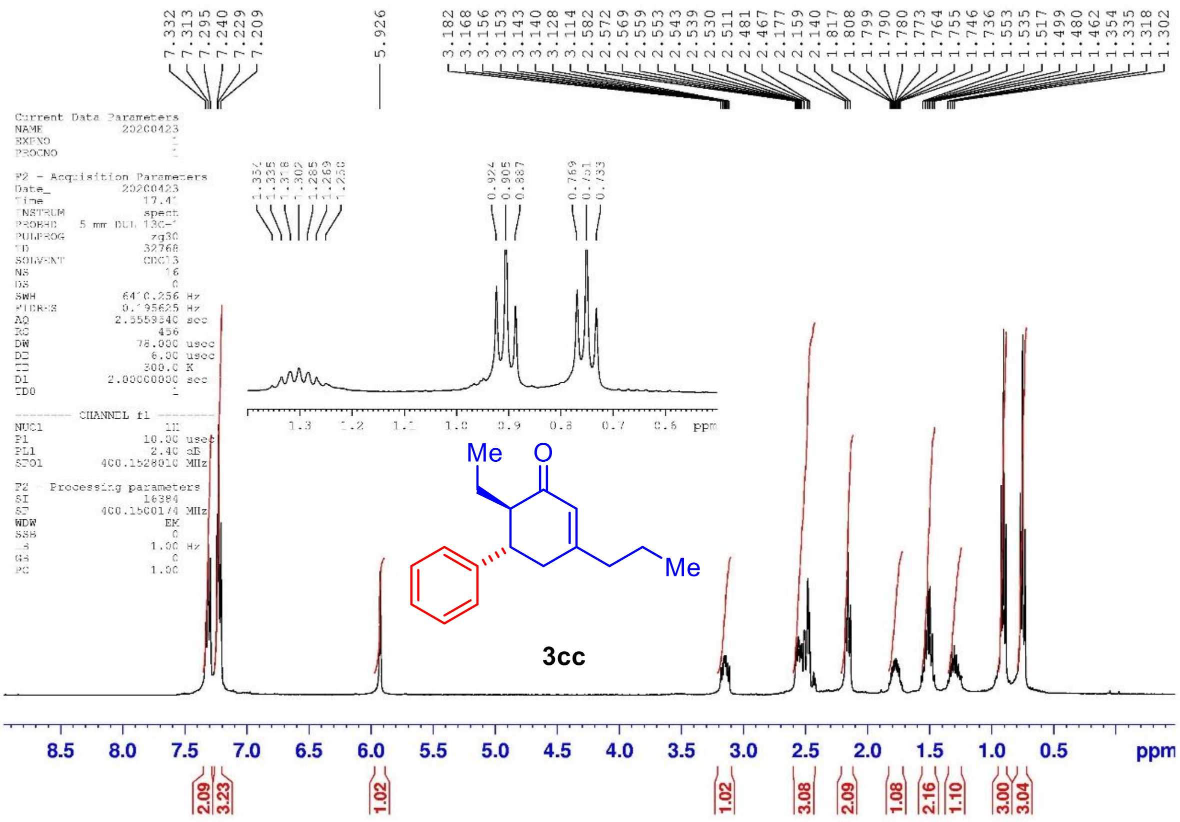

^{1}H NMR (400 MHz, $CDCl_3$) spectrum of compound **3cc**

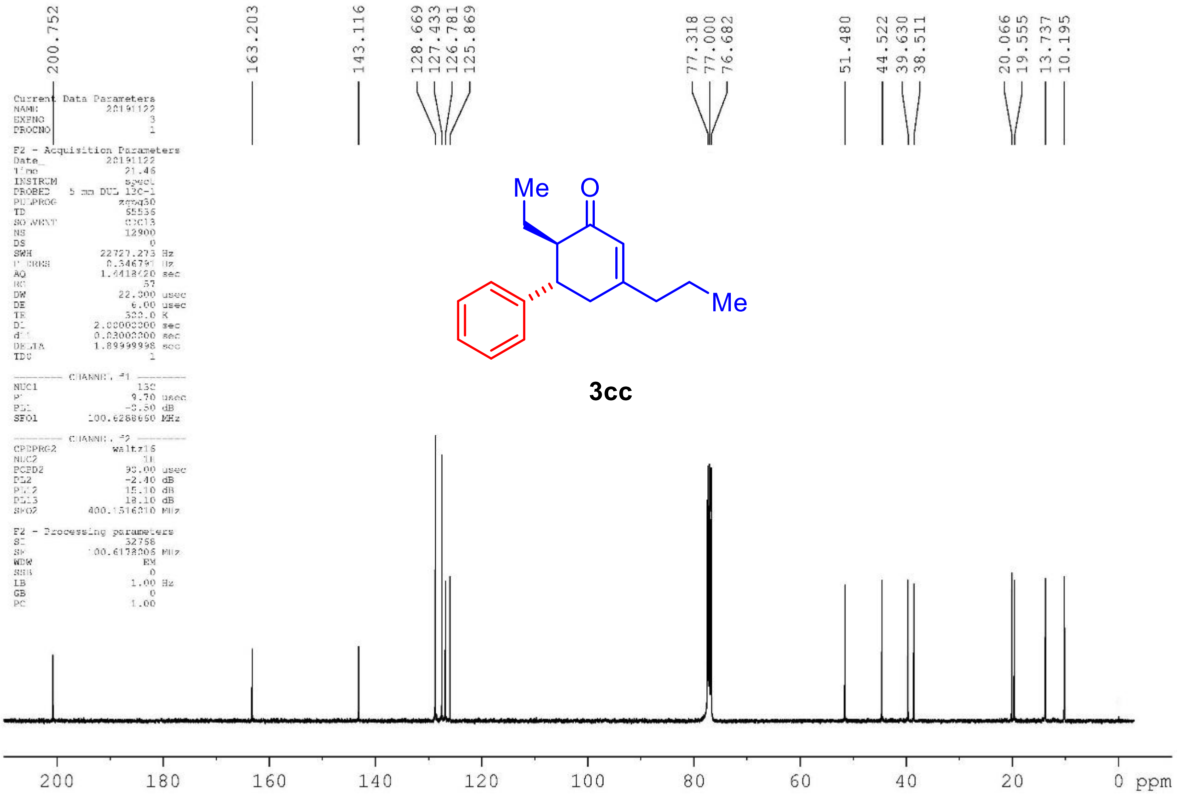

^{13}C{^{1}H} NMR (100 MHz, $CDCl_3$) spectrum of compound **3cc**

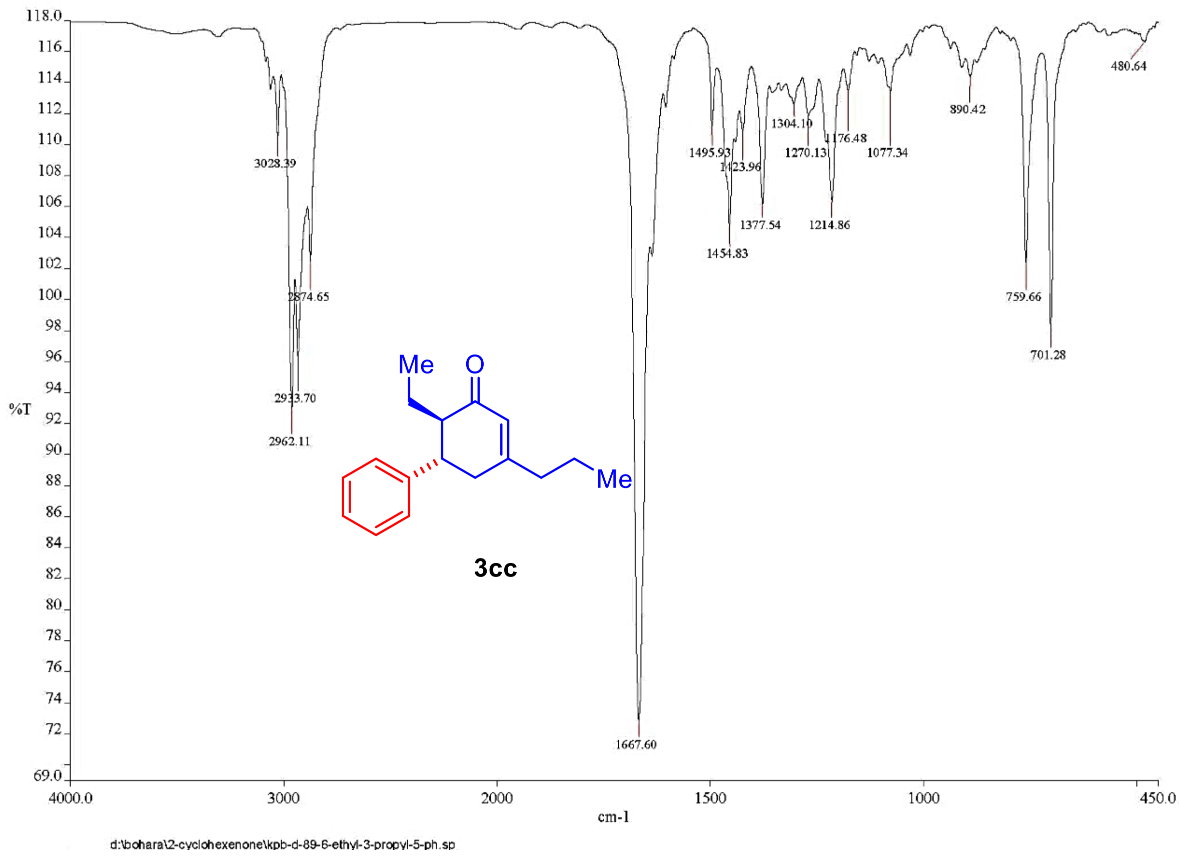

IR spectrum of compound **3cc**

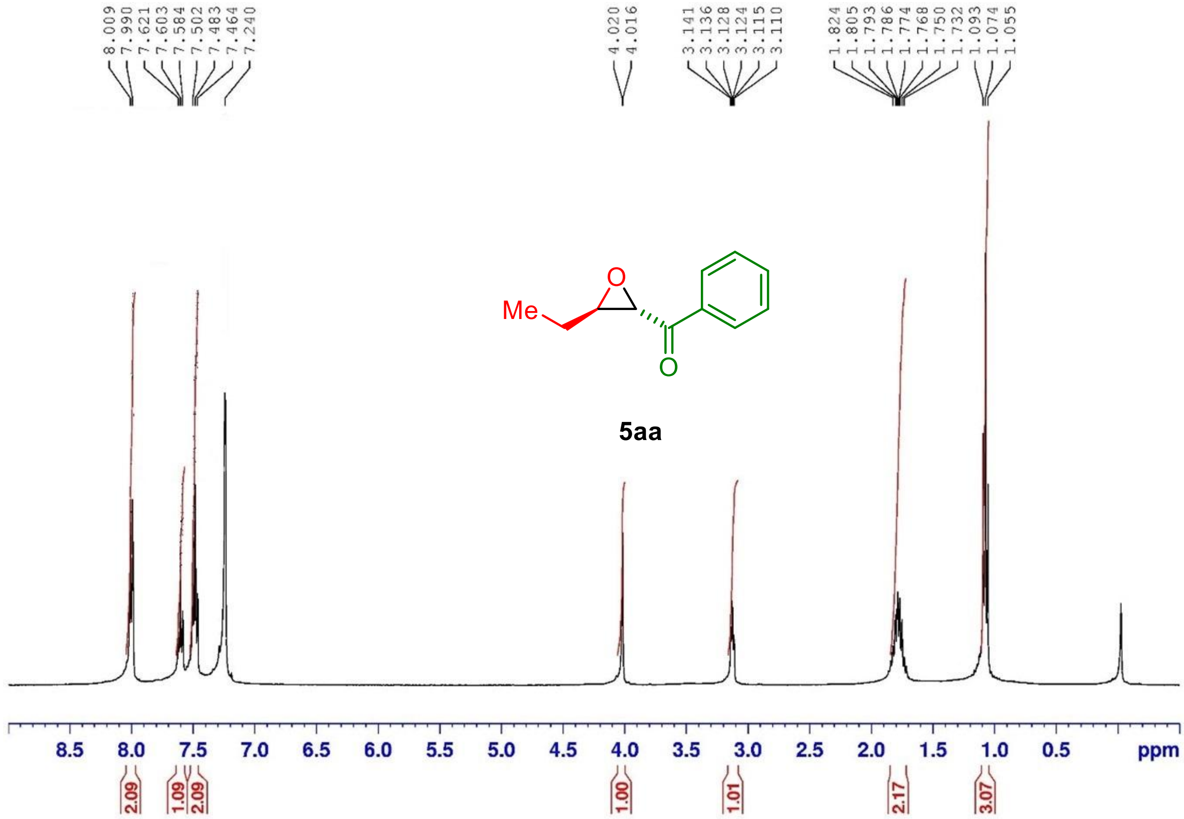

^{1}H NMR (400 MHz, $CDCl_3$) spectrum of compound **5aa**

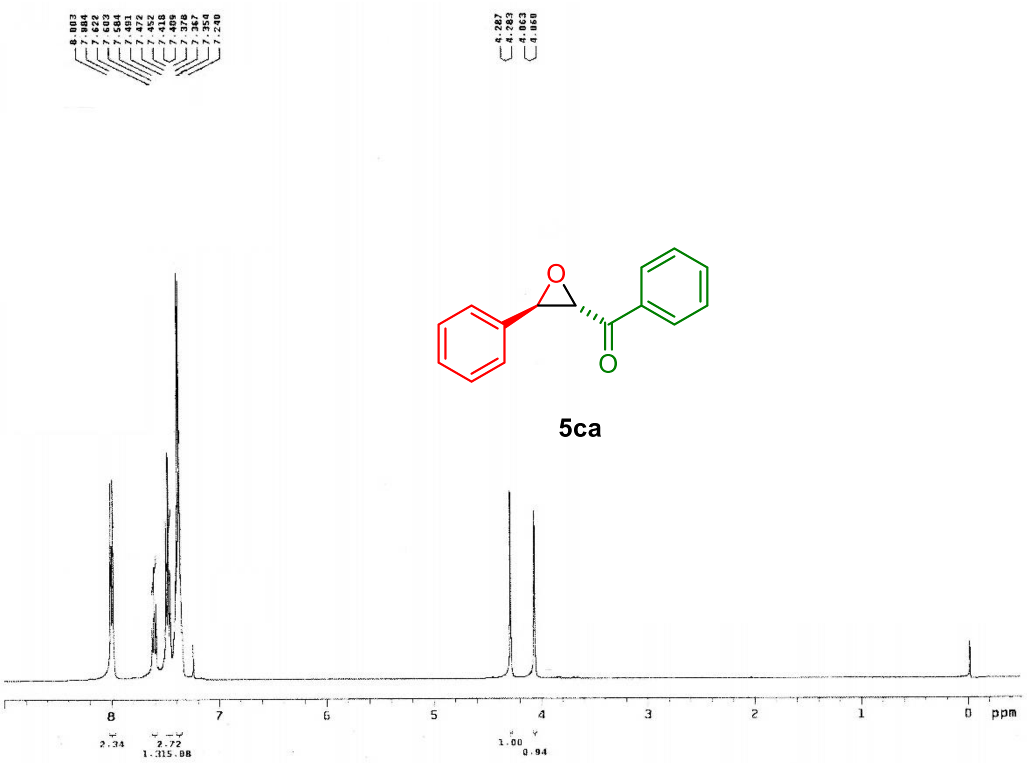

^{1}H NMR (400 MHz, $CDCl_3$) spectrum of compound **5ca**

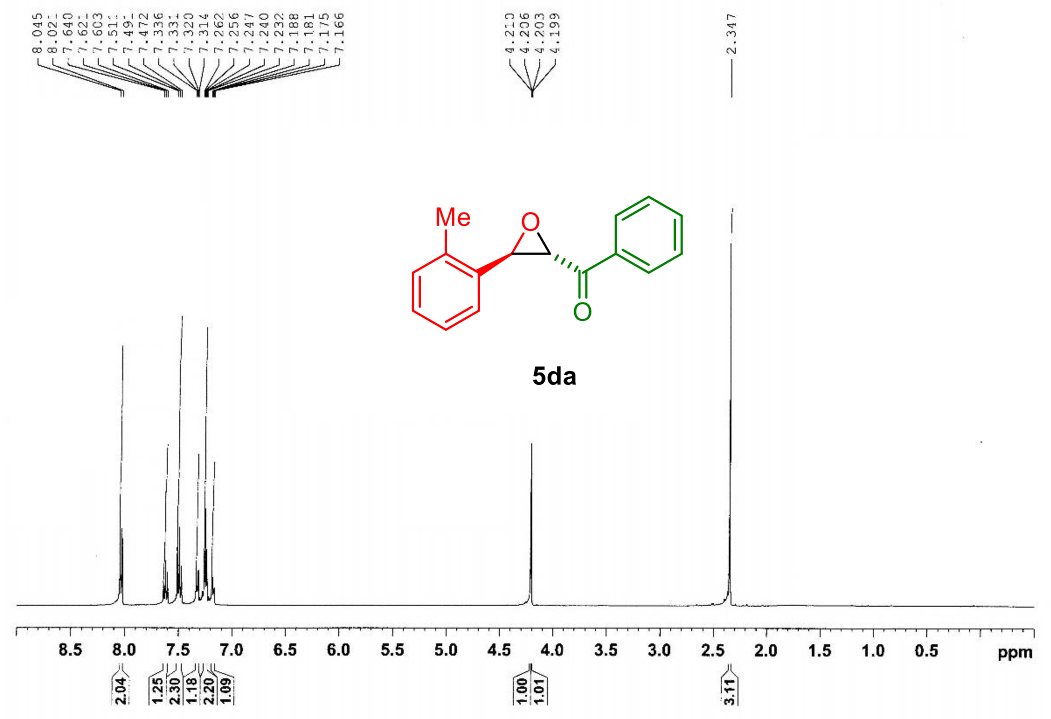

^{1}H NMR (400 MHz, $CDCl_3$) spectrum of compound **5da**

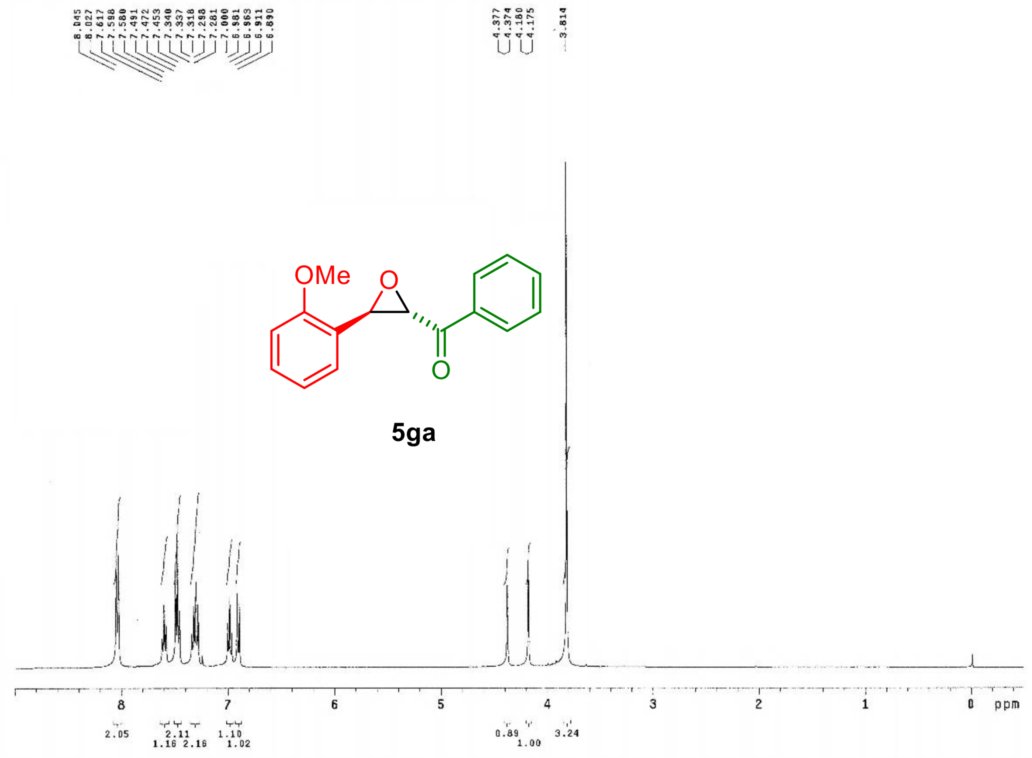

^{1}H NMR (400 MHz, $CDCl_3$) spectrum of compound **5ga**

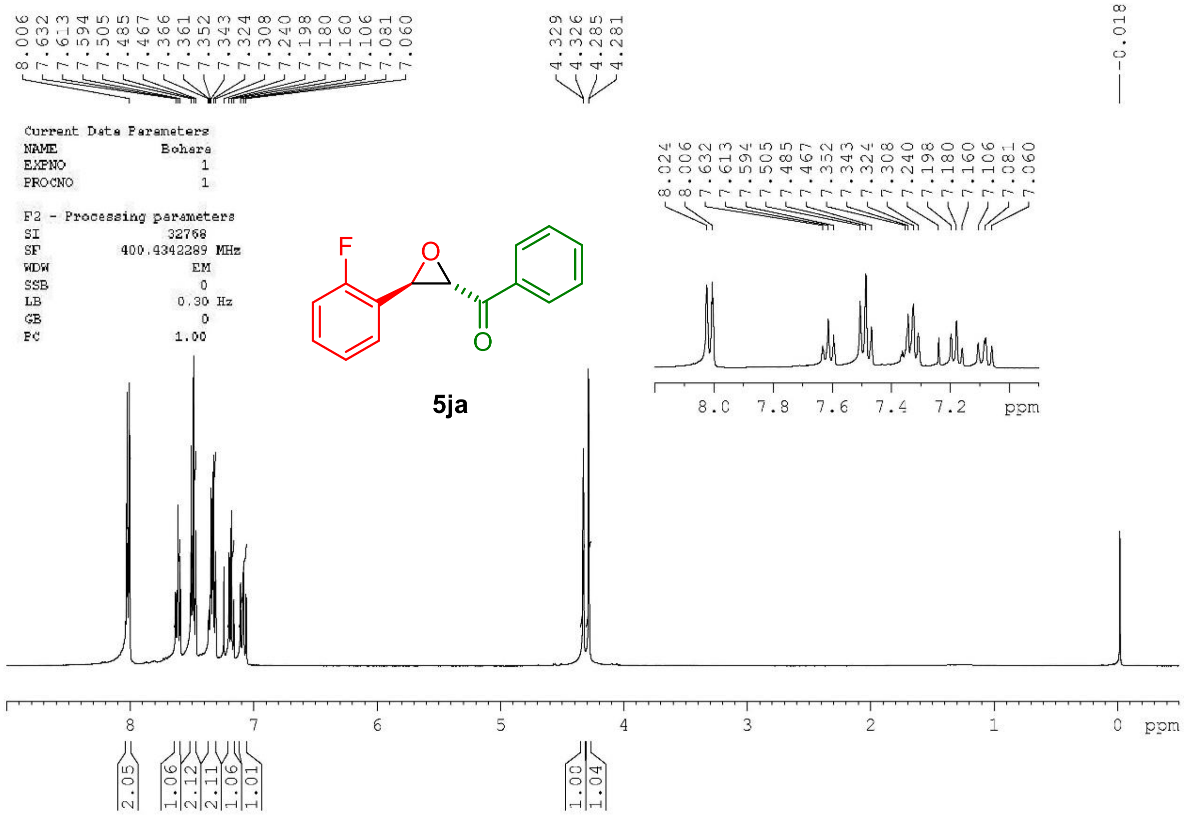

^{1}H NMR (400 MHz, $CDCl_3$) spectrum of compound **5ja**

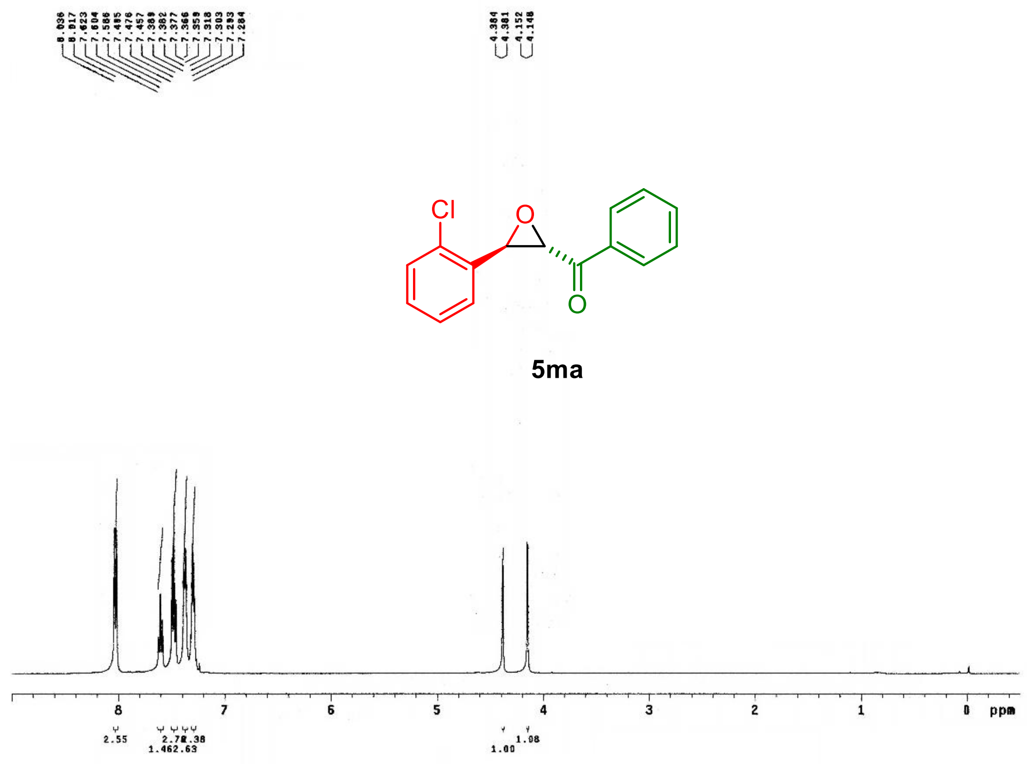

^{1}H NMR (400 MHz, $CDCl_3$) spectrum of compound **5ma**

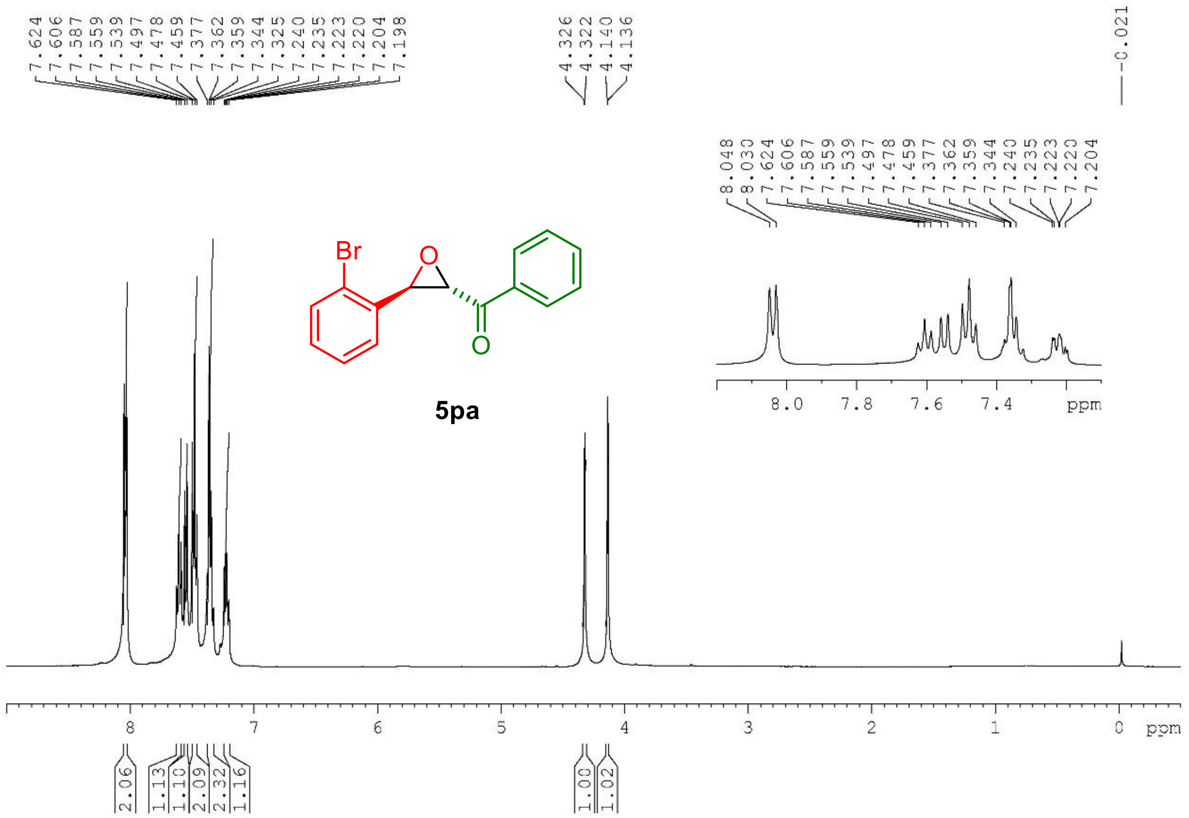

^{1}H NMR (400 MHz, $CDCl_3$) spectrum of compound **5pa**

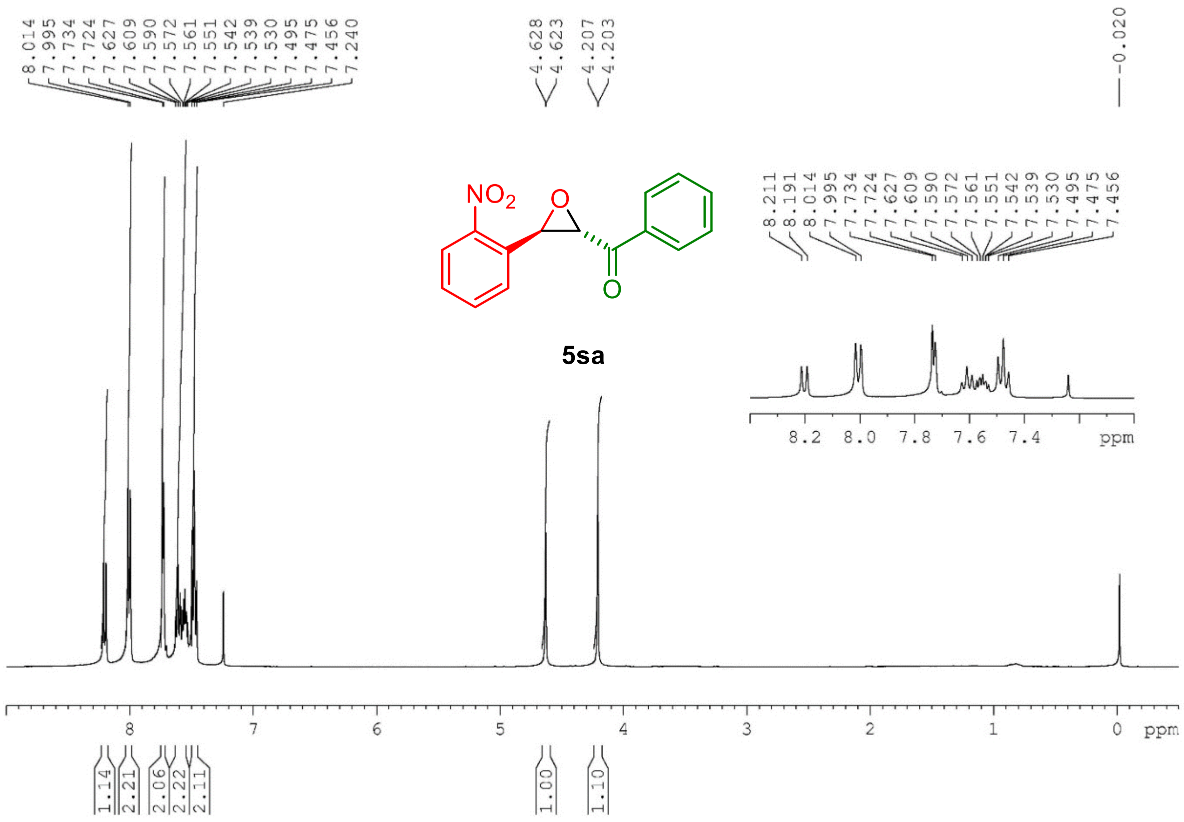

^{1}H NMR (400 MHz, $CDCl_3$) spectrum of compound **5sa**

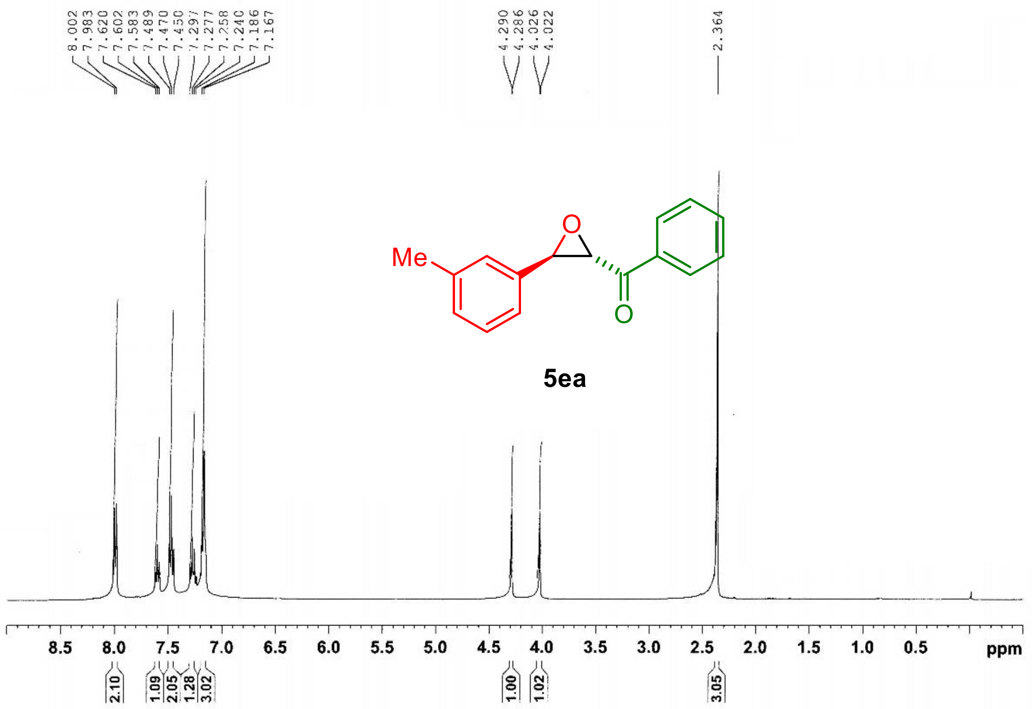

^{1}H NMR (400 MHz, $CDCl_3$) spectrum of compound **5ea**

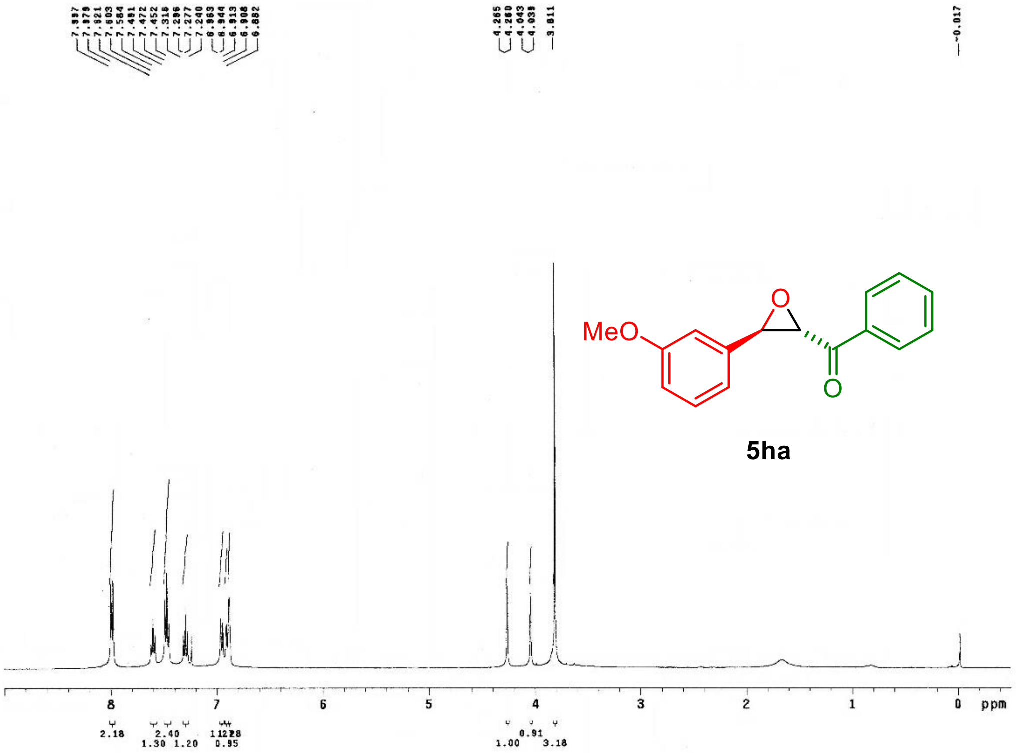

^{1}H NMR (400 MHz, $CDCl_3$) spectrum of compound **5ha**

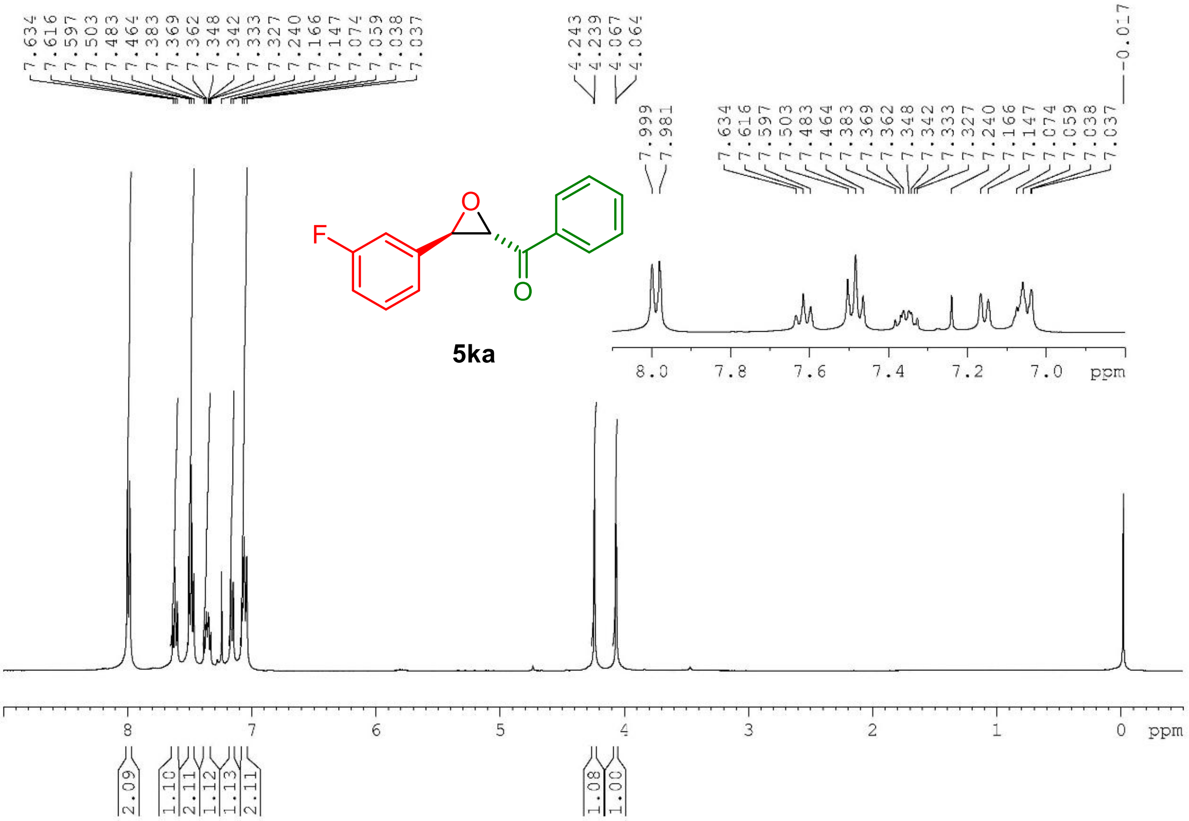

^{1}H NMR (400 MHz, $CDCl_3$) spectrum of compound **5ka**

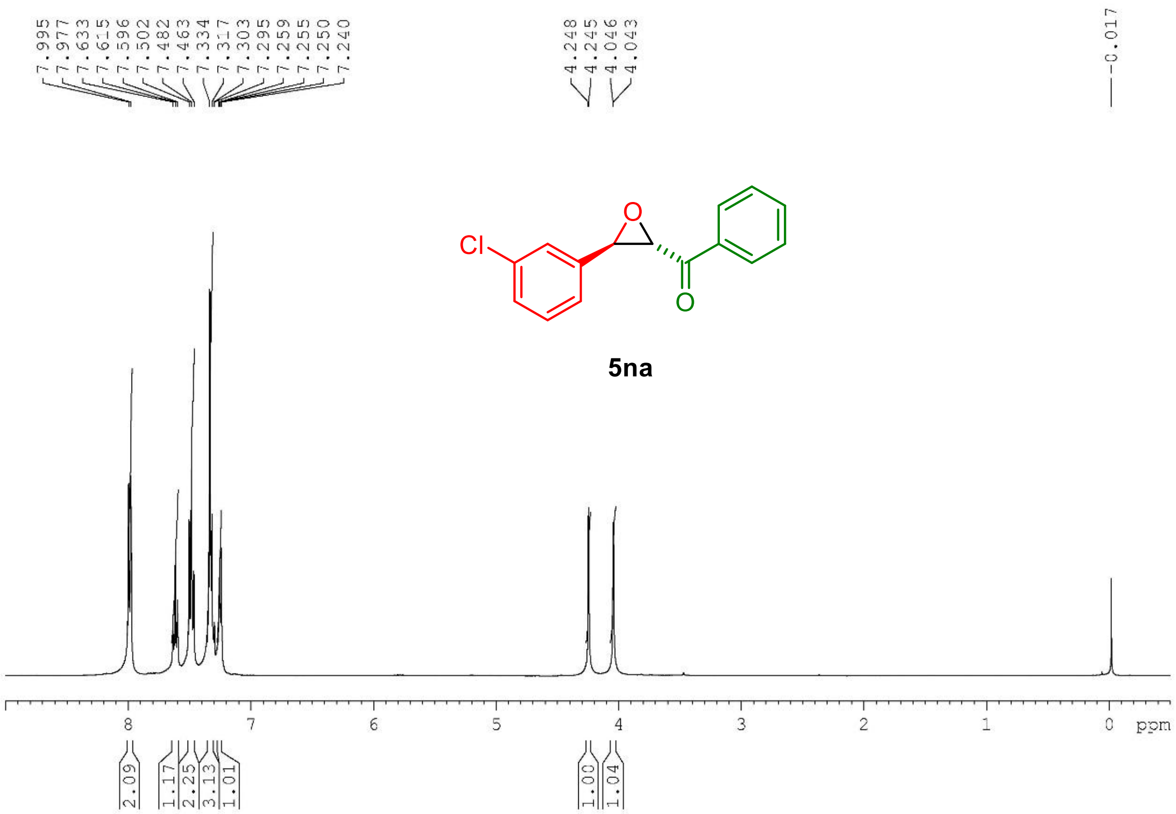

^{1}H NMR (400 MHz, $CDCl_3$) spectrum of compound **5na**

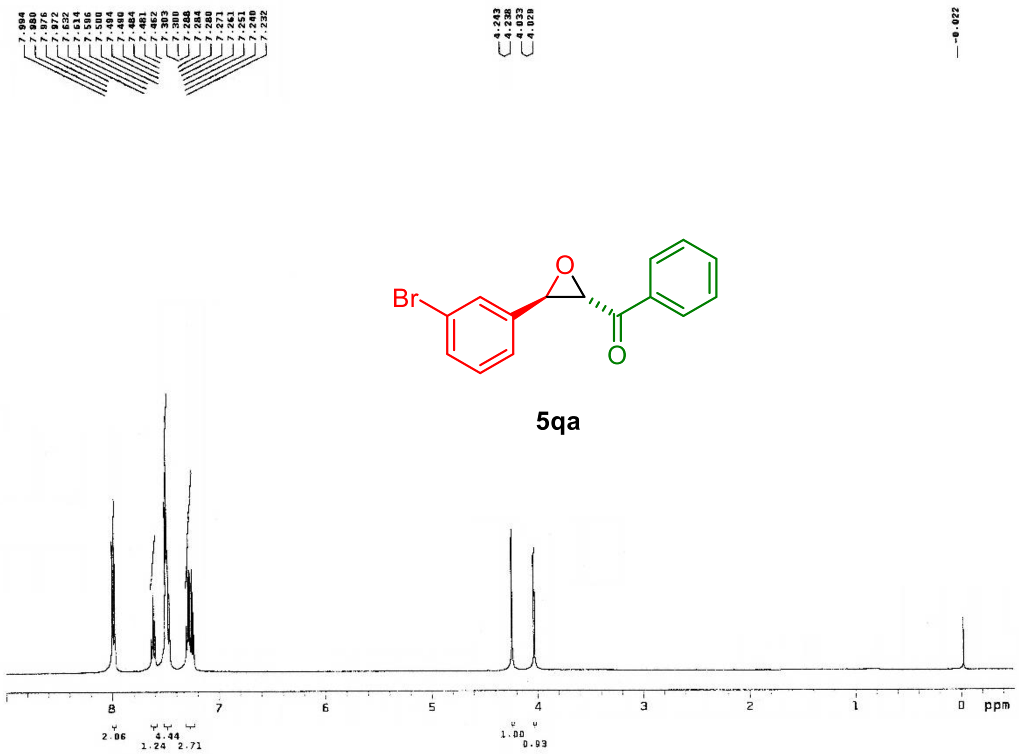

^{1}H NMR (400 MHz, $CDCl_3$) spectrum of compound **5qa**

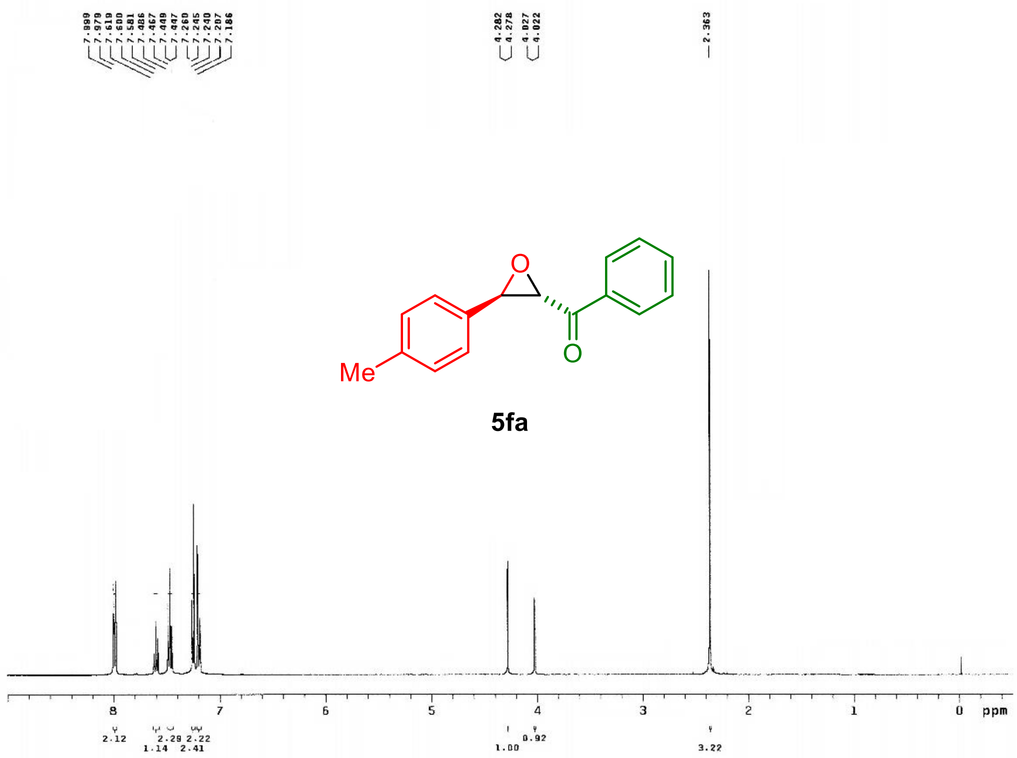

^{1}H NMR (400 MHz, $CDCl_3$) spectrum of compound **5fa**

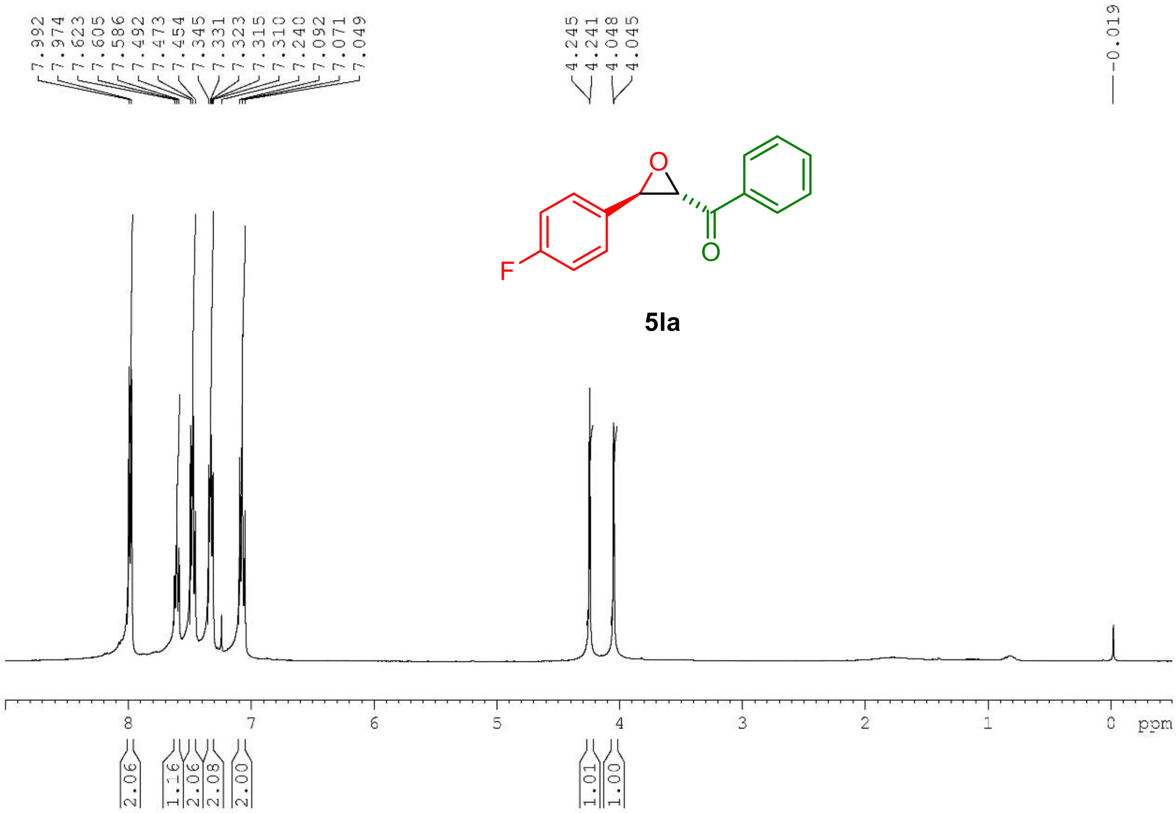

^{1}H NMR (400 MHz, $CDCl_3$) spectrum of compound **5la**

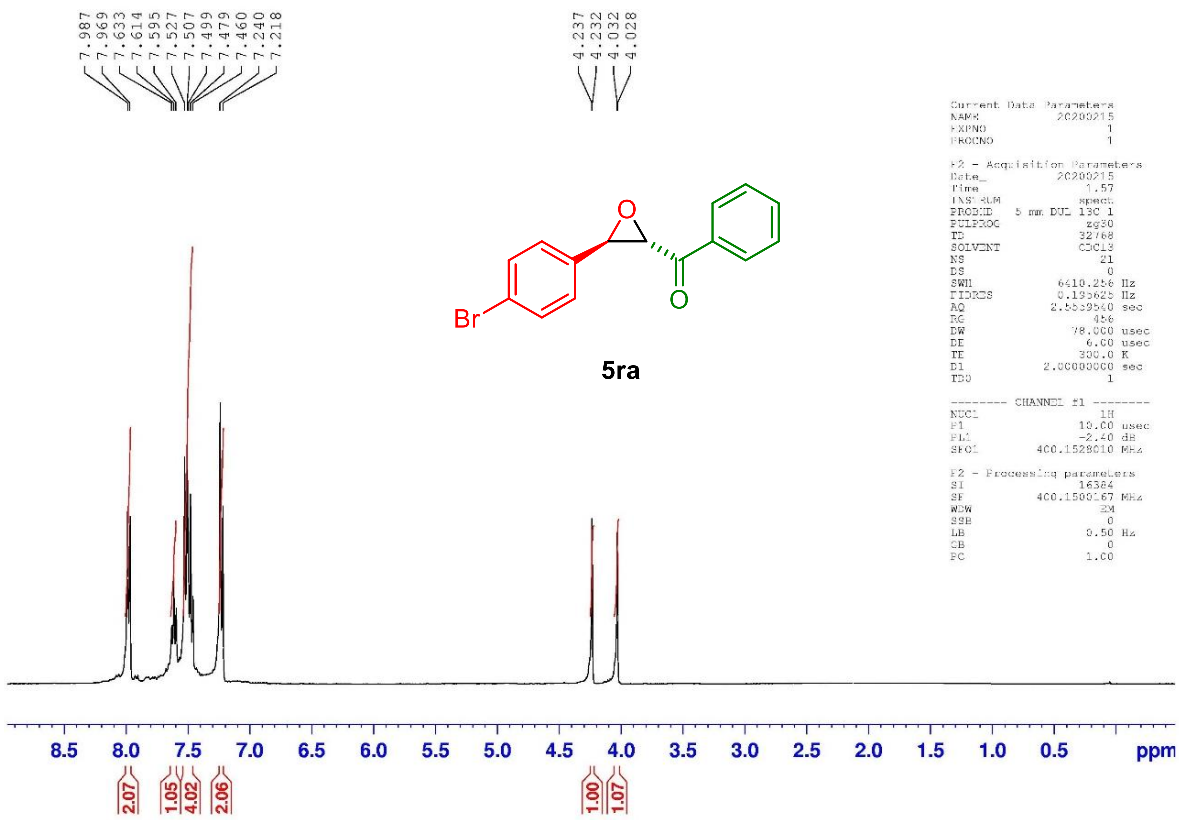

^1H NMR (400 MHz, $CDCl_3$) spectrum of compound **5ra**

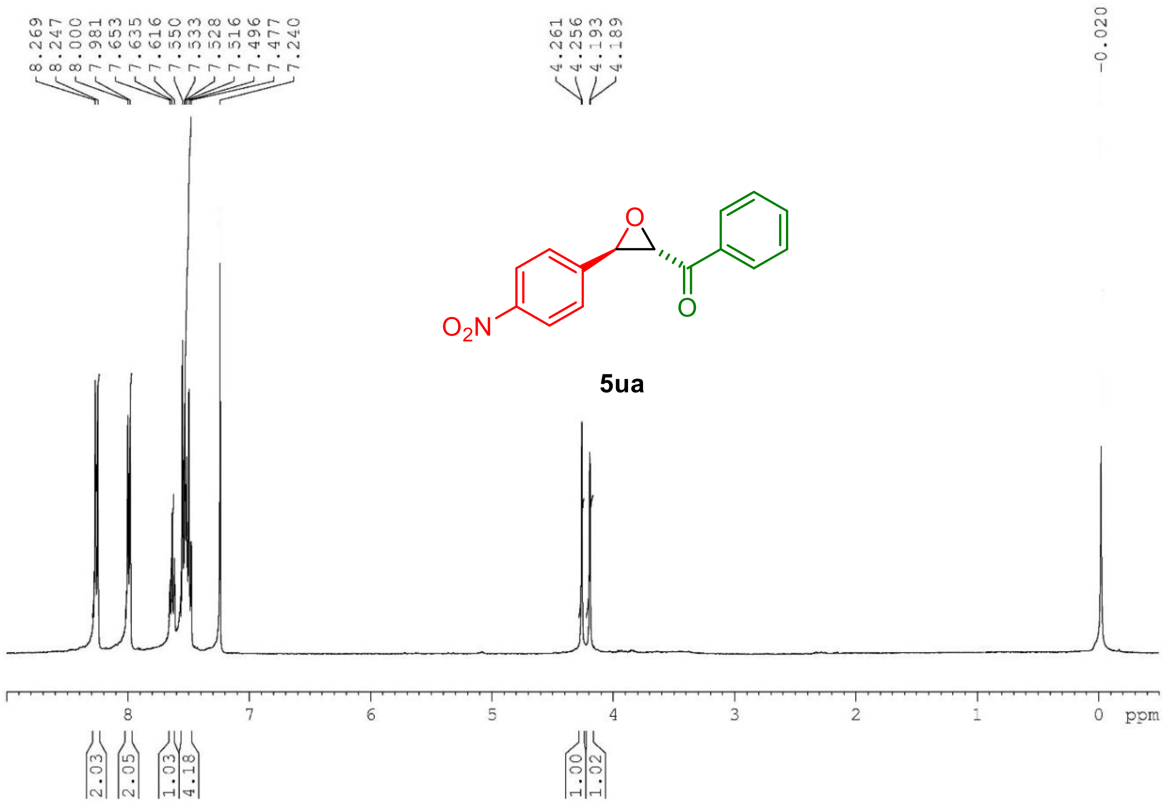

^1H NMR (400 MHz, $CDCl_3$) spectrum of compound **5ua**

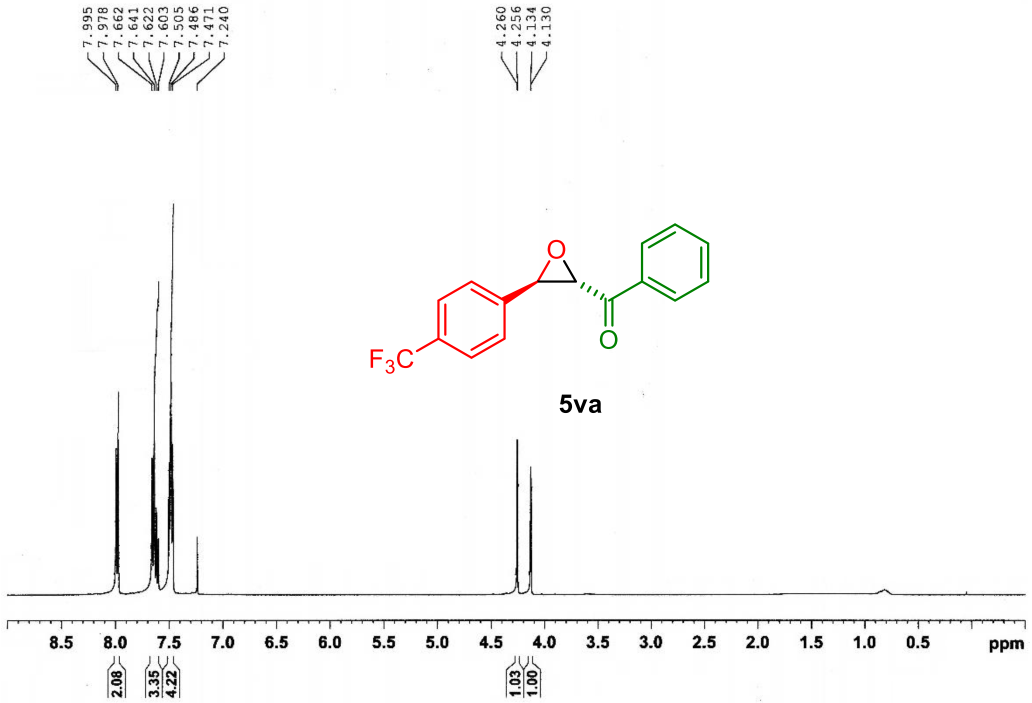

^{1}H NMR (400 MHz, CDCl3) spectrum of compound **5va**

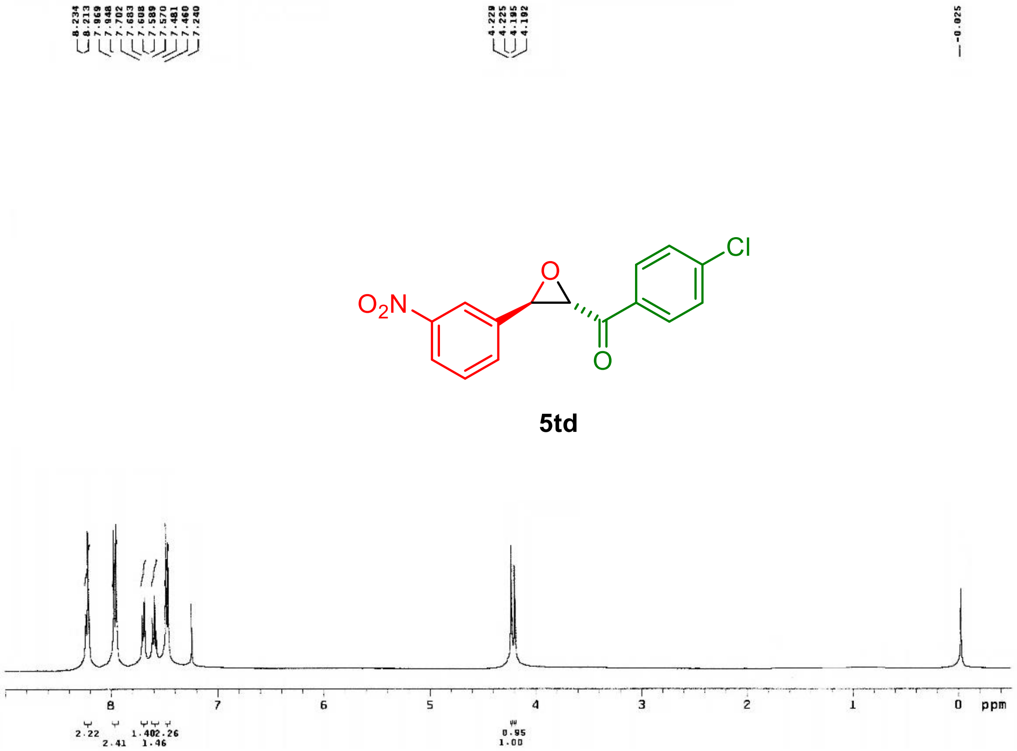

^{1}H NMR (400 MHz, CDCl3) spectrum of compound **5td**

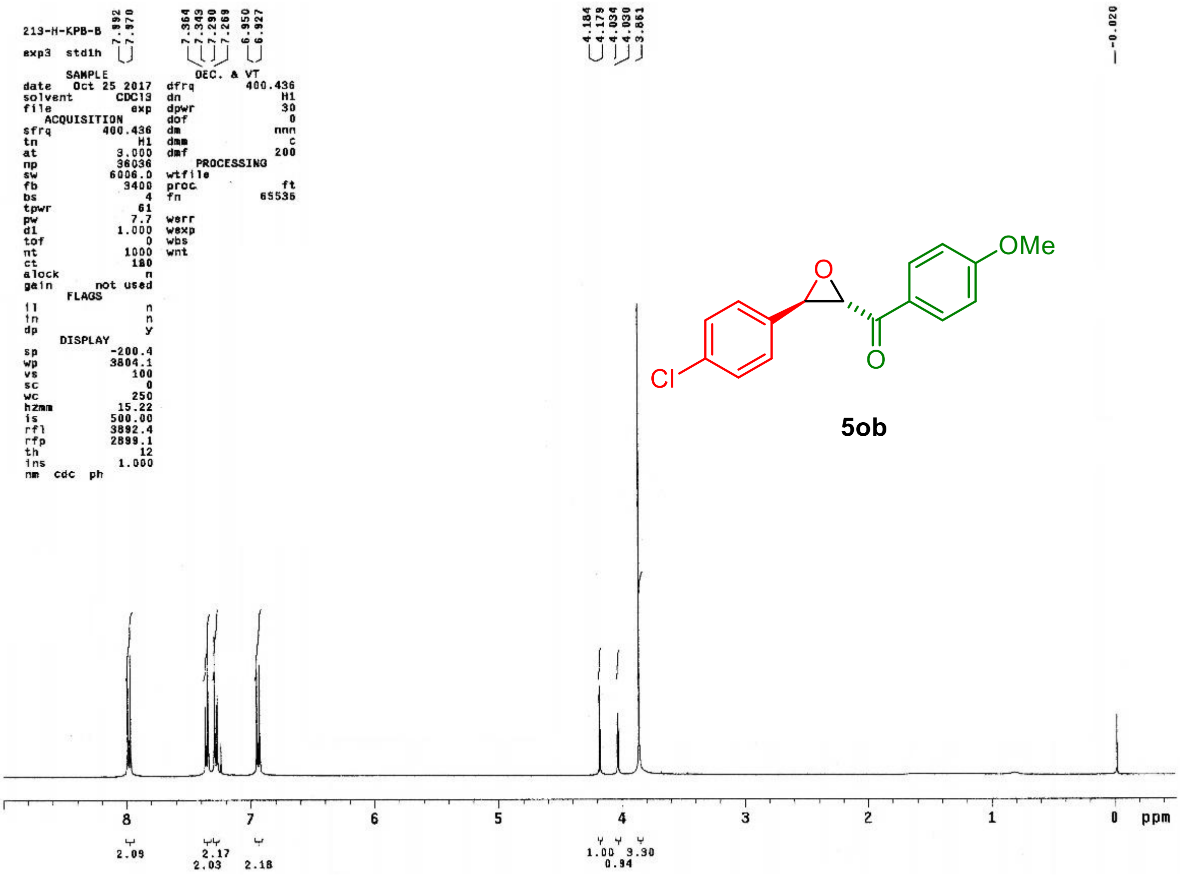

^{1}H NMR (400 MHz, $CDCl_3$) spectrum of compound **5ob**

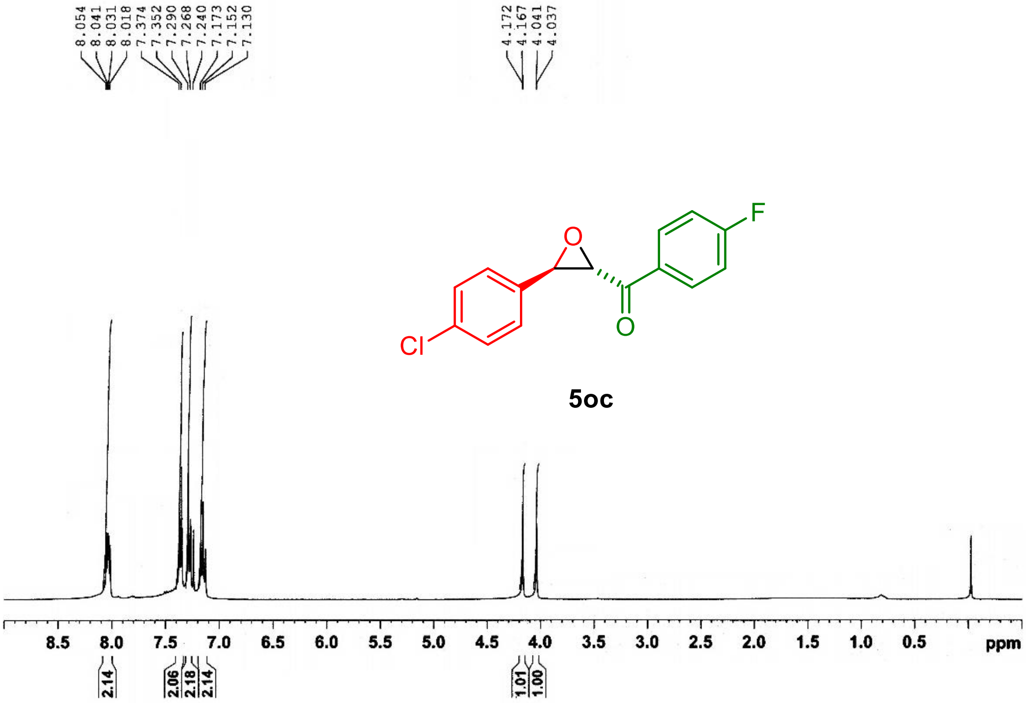

^{1}H NMR (400 MHz, $CDCl_3$) spectrum of compound **5oc**

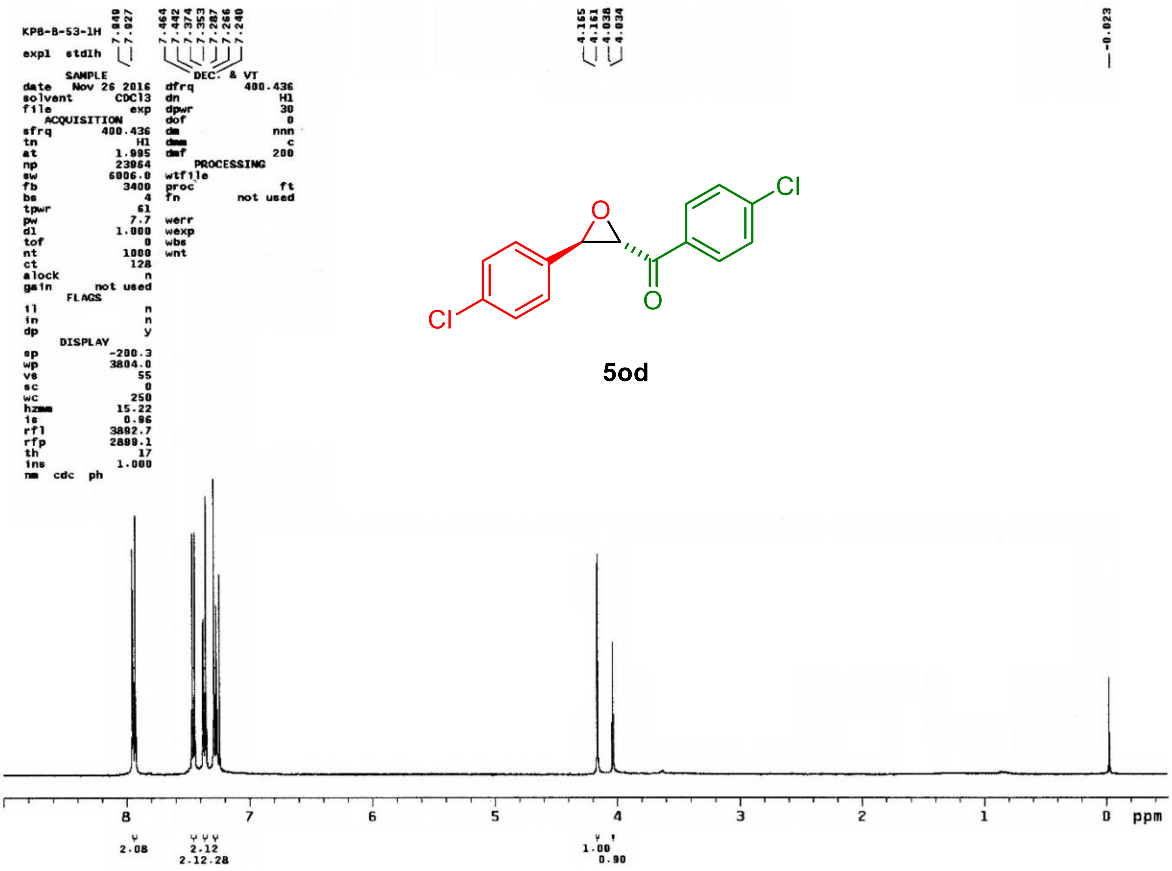

^{1}H NMR (400 MHz, $CDCl_3$) spectrum of compound **5od**

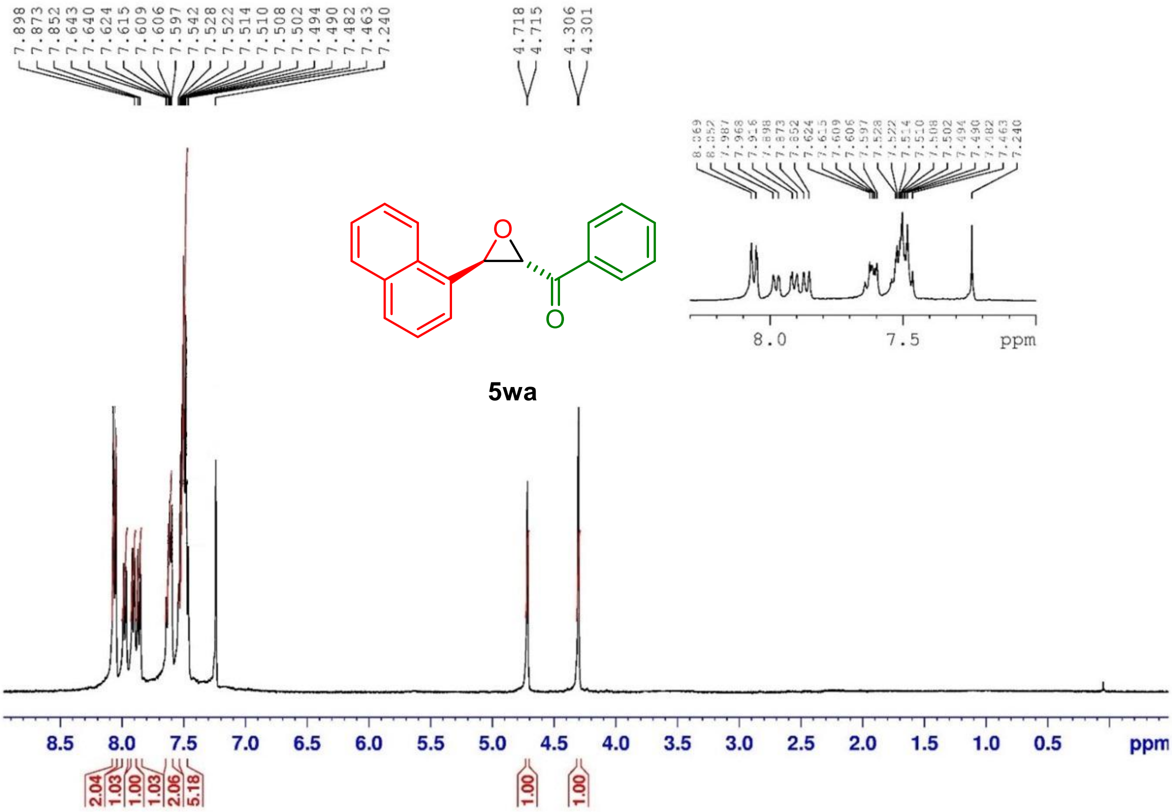

^{1}H NMR (400 MHz, $CDCl_3$) spectrum of compound **5wa**

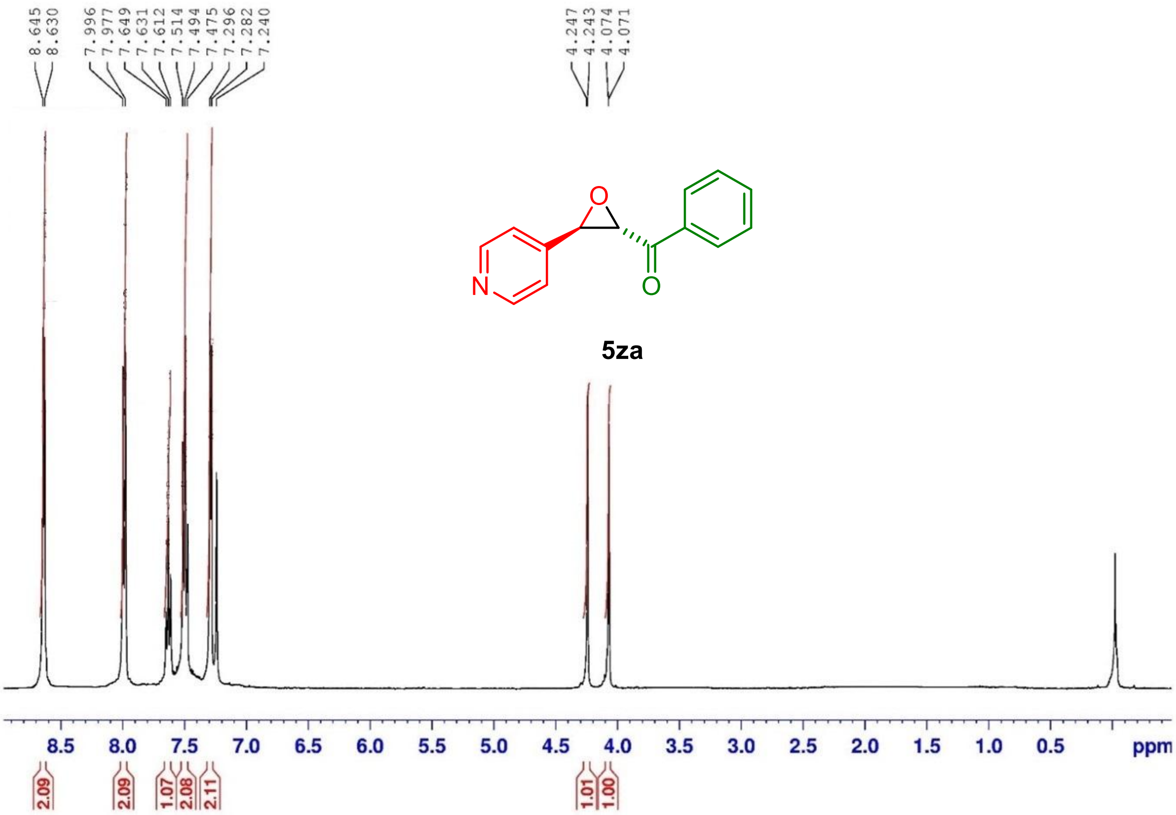

^{1}H NMR (400 MHz, CDCl$_3$) spectrum of compound **5za**

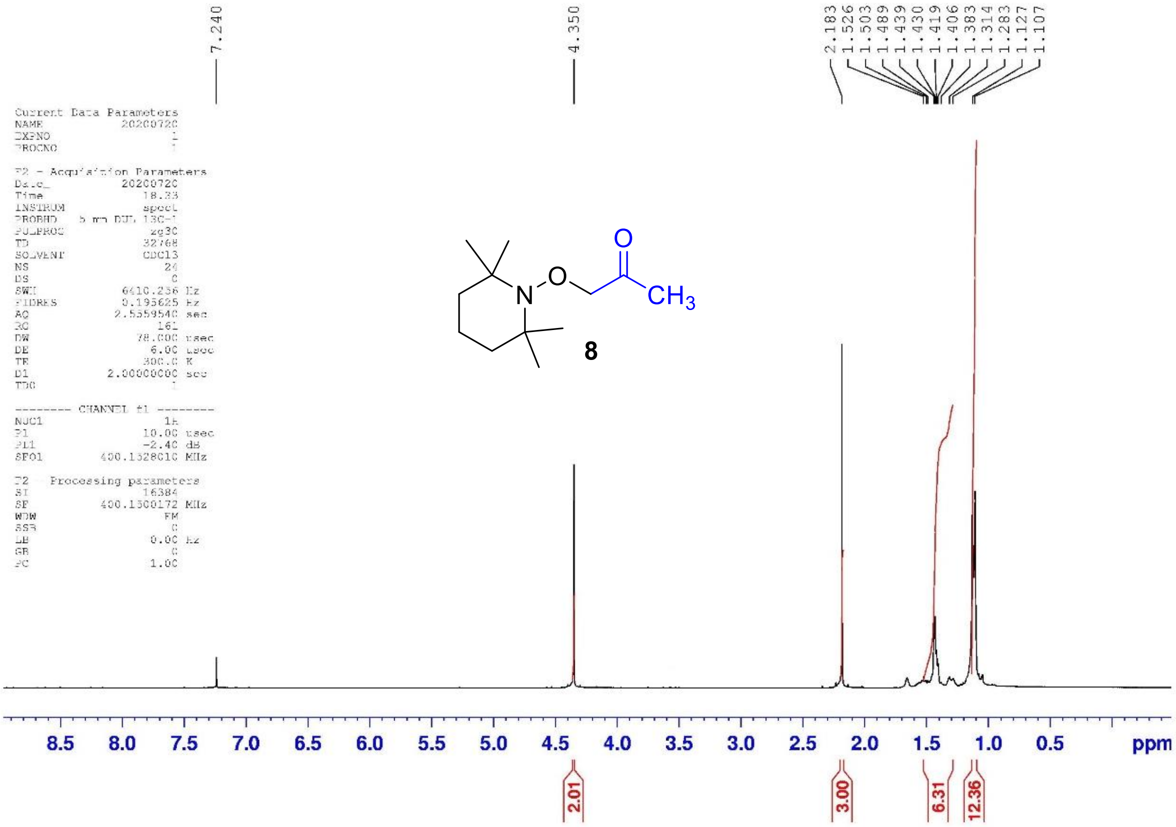

^{1}H NMR (400 MHz, CDCl$_3$) spectrum of compound **8**
